# Vitamin D supplementation in pregnancy and lactation to promote infant growth 

**DOI:** 10.1056/NEJMoa1800927

**Published:** 2018-08-09

**Authors:** Daniel E. Roth, Shaun K. Morris, Stanley Zlotkin, Alison D. Gernand, Tahmeed Ahmed, Shaila Sharmeen Shanta, Eszter Papp, Jill Korsiak, Joy Shi, M. Munirul Islam, Ishrat Jahan, Farhana Khanam Keya, Andrew R. Willan, Rosanna Weksberg, Minhazul Mohsin, Qazi Sadeq-ur Rahman, Prakesh S. Shah, Kellie E. Murphy, Jennifer Stimec, Lisa G. Pell, Huma Qamar, Abdullah Al Mahmud

**Affiliations:** Department of Pediatrics, University of Toronto and Centre for Global Child Health, The Hospital for Sick Children, Toronto, Canada; Department of Pediatrics, University of Toronto and Centre for Global Child Health, The Hospital for Sick Children, Toronto, Canada; Department of Pediatrics, University of Toronto and Centre for Global Child Health, The Hospital for Sick Children, Toronto, Canada; Department of Nutritional Sciences, Penn State University, University Park, Pennsylvania; Nutrition and Clinical Services Division, icddr,b, Dhaka, Bangladesh; Nutrition and Clinical Services Division, icddr,b, Dhaka, Bangladesh; Department of Pediatrics, University of Toronto and Centre for Global Child Health, The Hospital for Sick Children, Toronto, Canada; Department of Pediatrics, University of Toronto and Centre for Global Child Health, The Hospital for Sick Children, Toronto, Canada; Department of Pediatrics, University of Toronto and Centre for Global Child Health, The Hospital for Sick Children, Toronto, Canada; Nutrition and Clinical Services Division, icddr,b, Dhaka, Bangladesh; Maternal and Child Health Training Institute, Dhaka, Bangladesh; Nutrition and Clinical Services Division, icddr,b, Dhaka, Bangladesh; Ontario Child Health Support Unit at the Hospital for Sick Children Research Institute and Dalla Lana School of Public Health, University of Toronto, Toronto, Canada; Department of Pediatrics, University of Toronto and Centre for Global Child Health, The Hospital for Sick Children, Toronto, Canada; Nutrition and Clinical Services Division, icddr,b, Dhaka, Bangladesh; Nutrition and Clinical Services Division, icddr,b, Dhaka, Bangladesh; Department of Pediatrics, Mt. Sinai Hospital, Toronto, Canada; Department of Obstetrics and Gynecology, Sinai Hospital, Toronto, Canada; Department of Pediatrics, University of Toronto and Centre for Global Child Health, The Hospital for Sick Children, Toronto, Canada; Department of Pediatrics, University of Toronto and Centre for Global Child Health, The Hospital for Sick Children, Toronto, Canada; Department of Pediatrics, University of Toronto and Centre for Global Child Health, The Hospital for Sick Children, Toronto, Canada; Nutrition and Clinical Services Division, icddr,b, Dhaka, Bangladesh

## Abstract

**BACKGROUND:**

Causes of early infant growth restriction remain incompletely understood. Where vitamin D deficiency is common, vitamin D supplementation during pregnancy and lactation may improve fetal-infant growth and other birth outcomes.

**METHODS:**

We conducted a randomized, double-blind, placebo-controlled trial of maternal vitamin D supplementation from 17-24 weeks gestation until birth or 6 months postpartum. Participants were randomly allocated to five vitamin D and/or placebo supplementation groups: (A) 0 IU/week, (B) 4200 IU/week, (C) 16800 IU/week, or (D) 28000 IU/week in pregnancy, all with 0 IU/week postpartum; or, (E) 28000 IU/week in prenatal and postpartum periods. The primary outcome was length-for-age z-score at one year of age according to World Health Organization child growth standards.

**RESULTS:**

Among 1164 infants assessed at one year of age (90% of 1300 pregnancies), there were no differences across groups in length-for-age z-scores (mean ±standard deviation): A: -0.93 ±1.05, B: -1.11 ±1.12, C: -0.97 ±0.97, D: -1.06 ±1.07, E: -0.94 ±1.00 (p=0.23). Groups were similar with respect to other anthropometric measures, birth outcomes, and morbidity. Vitamin D had dose- dependent effects on maternal and infant serum 25-hydroxyvitamin D and calcium, maternal urinary calcium excretion, and maternal parathyroid hormone concentrations. No clinical adverse events were attributed to the vitamin D intervention.

**CONCLUSIONS:**

In a population with widespread prenatal vitamin D deficiency and fetal/infant growth restriction, maternal vitamin D supplementation from mid-pregnancy until birth or 6 months postpartum does not influence fetal or infant growth, and has no beneficial or harmful effects on numerous other birth and infant outcomes.

## BACKGROUND

Environmental and dietary regulation of fetal and infant growth remains inadequately understood. Observational studies have demonstrated associations of early-life exposures with later anthropometric outcomes^1,2^, but there is limited evidence of effects of prenatal nutritional interventions on childhood linear growth^3^. Small-for-gestational age (SGA) and postnatal linear growth faltering continue to be major public health problems in low- and middle-income countries^4,5^.

Vitamin D may influence fetal and postnatal growth through effects on calcium absorption^6^, parathyroid hormone (PTH) expression^7^, phosphate metabolism^8^, growth plate function^9,10^, and possible regulation of the insulin-like growth factor axis^11^. Meta-analyses of observational studies^12^ and clinical trials^13^ have suggested a possible beneficial effect of vitamin D on fetal growth, but most previous trials had important methodological limitations^13^. In a previous small trial in Bangladesh, we found that early postnatal linear growth was accelerated in infants born to vitamin D-supplemented mothers^14^.

In Bangladesh, 30% of newborns are SGA^4^ and 36% of children under 5-years of age are stunted (height-for-age z-score <-2)^15^. Vitamin D deficiency is common in Bangladeshi women of reproductive age^16^. In this Maternal Vitamin D for Infant Growth (MDIG) trial, we aimed to evaluate the dose-dependent effects of prenatal vitamin D supplementation, with and without postpartum supplementation, on infant growth and other maternal, newborn and infant outcomes in Dhaka, Bangladesh.

## METHODS

### Trial Design and Oversight

MDIG was a randomized, double-blind (participants and study personnel), placebo-controlled, dose-ranging parallel five-arm trial of maternal vitamin D supplementation^17^. The protocol and statistical analysis plan are available at NEJM.org. The study was overseen by a trial steering committee and an independent data and safety monitoring board. The protocol was approved by research ethics committees at The Hospital for Sick Children (Toronto, Canada; REB1000039072) and icddr,b (PR-13055). All authors contributed to finalizing the manuscript and attest to the completeness and accuracy of analyses and adherence to the protocol. The trial funder had no role in trial design, data collection, analysis, or interpretation of the results.

### Participants

Generally healthy women between 17 and 24 weeks of gestation were enrolled after providing written informed consent between March 2014 and September 2015 at the Maternal and Child Health Training Institute (MCHTI), a public hospital in Dhaka, Bangladesh. Inclusion and exclusion criteria are shown in Table S1 in the Supplementary Appendix.

### Interventions

Participants were randomly allocated at enrolment to one of five groups: 0 IU/week vitamin D from enrolment until delivery and 0 IU/week from 1 to 26 weeks postpartum (0;0 or ‘placebo’ group); 4200 IU/week prenatal and 0 IU/week postpartum (4200;0); 16800 IU/week prenatal and 0 IU/week postpartum (16800;0); 28000 IU/ prenatal and 0 IU/week postpartum (28000;0); or, 28000 IU/week prenatal and postpartum (28000;28000). A computer-generated simple randomization scheme was created independently by the trial statistician (A.R.W.). The master list linking unique participant identifiers to supplementation groups was held by the supplement manufacturer and not accessed by any study personnel until final unmasking. Allocation concealment was ensured by using pre-labeled sequentially-numbered and otherwise identical supplement vials assigned to participants according to the allocation sequence. Oral vitamin D3 and placebo tablets were manufactured by Toronto Institute for Pharmaceutical Technology (Toronto, Canada). Vitamin D content of each batch of tablets was verified in product testing^17^. Tablets of varying doses were identical in appearance and taste. Tablets were routinely administered under direct observation by study personnel; however, up to 4 consecutive doses could be unobserved when participants were unavailable for scheduled visits. Missed doses were administered up to 7 days late. Calcium (500 mg/day), iron (66 mg/day), and folic acid (350 μg/day) were provided to all participants throughout the intervention phase^17^. A mid-trial audit of self-reported calcium and iron-folic acid adherence revealed that >85% participants reported >85% adherence. If a participant reported non-study vitamin D or calcium supplement use for >1 week, study supplements were suspended until non-study supplement use was discontinued. Supplementation was discontinued in participants with confirmed hypercalcemia (see definition below), fetal or infant death, or a new condition or medication that could alter vitamin D metabolism. 

### Assessments

Study personnel contacted participants at weekly intervals from enrolment until 26 weeks postpartum, then every three months. Visits were conducted in the home or clinic, and included standardized questionnaires, point-of-care tests, anthropometry, and specimen collection (Table S2 in the Supplementary Appendix). Socioeconomic and household characteristics were collected at baseline. Weekly prenatal questionnaires included healthcare encounters and a clinical symptom checklist. Postnatal follow-ups included history of the infant’s health and feeding practices, and a basic physical exam of the infant. Maternal blood pressure was routinely measured at enrolment, 24 and 30 weeks gestation, and weekly from 36 weeks gestation to delivery. Study personnel tracked pregnancy outcomes, study physician encounters, hospitalizations and deaths. They attended all facility-based deliveries and home births when feasible. Participants were encouraged to seek medical attention from study physicians or to notify study personnel of any concerns. Free medical care was provided throughout the trial. Pregnancies were completed from June 2014 to February 2016; one-year postnatal visits were conducted from June 2015 to March 2017.

Infant crown-to-heel length (to the last completed 0.1 cm), head circumference (HC), upper arm length (UAL), mid-upper arm circumference (MUAC), and rump-to-knee length (RKL) – all to the last completed 0.1 cm – and weight (to the nearest 5 g up to 10 kg, and nearest 10 g for >10 kg) were measured according to standardized procedures by trained personnel, as previously described^17^ and adapted from Intergrowth-21st protocols^18^. Length, weight, HC, UAL, and RKL were measured at birth. Length, weight, and HC were measured at a randomly timed visit during the first 2 months, and then at 3, 6, 9 and 12 months of age. MUAC, UAL, and RKL were measured at 3, 6, and 12 months. Each parameter was measured independently by two study personnel; paired measurements were compared and repeated if the difference exceeded 7 mm for length, 5 mm for HC, UAL, MUAC, or RKL, and 50 g for weight. Means of the final pair of values were used in analysis. Missing, outlying or implausible values were identified and interrogated (Method 1 in the Supplementary Appendix)^19^. There was high inter-rater reliability and few measurements were dropped due to implausibility or temporal inconsistencies (Table S3 in the Supplementary Appendix). Length, weight, weight-for-length (WFL), body mass index (BMI), HC, and MUAC were expressed as sex- and age- (or gestational age-) standardized z-scores using Intergrowth-21st standards for newborn size^20^, postnatal growth standards for preterm infants to 64 weeks post-menstrual age^21^ (weight, length, HC only), or World Health Organization (WHO) child growth standards^22^. 

Serum calcium (sCa) and urinary calcium:creatinine ratio (uCa:Cr) were measured by routine methods at icddr,b (Dhaka, Bangladesh). Serum 25-hydroxyvitamin D (25(OH)D) and intact parathyroid hormone (iPTH) measurements were performed by the Analytical Facility for Bioactive Molecules, The Hospital for Sick Children (Toronto, Canada)(Method 2 in the Supplementary Appendix). Biochemical screening for rickets was scheduled at 6 months of age (Method 3 in the Supplementary Appendix). Radiological confirmation of rickets was based on interpretations of wrist and/or knee radiographs by a pediatric radiologist (J.S.) blinded to clinical or laboratory data. Classification of infant neurological disabilities and congenital anomalies, and clustering of study physician-assigned diagnostic codes for clinical encounters and hospitalizations, were done by investigators (D.E.R., S.Z., S.K.M., R.W.), post-hoc but masked to treatment allocation. 

### Outcomes

The primary outcome was length-for-age z-score (LAZ) at one year (364–420 days) of age. Secondary anthropometric outcomes included weight-for-age z-score, WFL z-score, BMI-for-age z-score, HC-for-age z-score, MUAC-for-age z-score, UAL, and RKL (Table S4 in Supplementary Appendix). Stunting was defined as LAZ<-2. For measurements within 48 hours of birth, SGA was weight-for-age<10th percentile (using Intergrowth-21st newborn standards^20^) and low birth weight (LBW) was <2500 g. Preterm birth was gestational age (GA) at birth <37 weeks based on last menstrual period and 2nd trimester ultrasound (Table S1 in the Supplementary Appendix). Vitamin D status was based on serum 25(OH)D^23^; deficiency was defined as 25(OH)D<30 nmol/L^24^. The C3-epimer fraction was included in sensitivity analyses (Method 2 in the Supplementary Appendix). The primary safety measure was maternal total sCa measured at baseline, 30 weeks of gestation, delivery, 3-months and 6-months postpartum, or when hospitalized due to illness if feasible. Possible hypercalcemia was any sCa>2.60 mmol/L and confirmed hypercalcemia (primary safety outcome) was defined when sCa>2.60 mmol/L on a repeat specimen or a single sCa>2.80 mmol/L. Secondary safety indicators included infant sCa at 3 and 6 months of age and maternal urinary Ca:Cr at delivery. Maternal possible hypercalciuria was a single uCa:Cr>1 mmol/mmol. Participants with uCa:Cr>1 on two consecutive specimens (confirmed hypercalciuria) and/or symptoms of renal colic underwent abdominal ultrasound for uro- or nephrolithiasis. Infant uCa:Cr was measured at 6 months of age. Secondary clinical outcomes included gestational hypertension, delivery characteristics, stillbirth, congenital anomalies, rickets, clinical encounters, hospitalization and death (Table S4 in the Supplementary Appendix). 

### Statistical Analysis

The primary analysis was a complete-case intention-to-treat analysis. Analysis of variance (ANOVA) was performed to compare LAZ at one year of age across all groups. To estimate the effect of prenatal vitamin D (IU/week), five pairwise comparisons were conducted using t-tests: 4200;0 versus placebo, 16800;0 versus placebo, 16800;0 versus 4200;0, 28000;0 versus placebo, and 28000;0 versus 16800;0. Statistical significance was tested at an overall alpha=0.05 (two-sided), applying the Holm test for multiple comparisons^25^. Sample size determination conservatively assumed that if each between-group comparison had a two-sided alpha=0.01 and 90% power, 220 analyzable participants per group would enable detection of a between-group difference in LAZ of at least 0.40^14^. To accommodate 15% attrition, we aimed to enroll 260 pregnant women in each arm.

The effect of postpartum vitamin D on LAZ at one year of age was assessed by the pairwise comparison of 28000;28000 versus 28000;0 (two-sided alpha=0.05) using a t-test. Secondary outcomes were compared across groups using ANOVA for continuous normally-distributed variables and Kruskal-Wallis tests for skewed distributions; Chi-square and Fischer’s exact tests were used for categorical variables. Zero-inflated negative binomial models were used to compare incidence rates of clinical encounters, hospitalizations, and other adverse events. Where a global test was significant at p<0.05, post-hoc pairwise comparisons were performed, applying the Holm test for multiplicity^25^. We conducted sensitivity and stratified analyses of the primary outcome, including multiple imputation by chain equations to account for missing length at one year of age (Method 4 in the Supplementary Appendix). Infant LAZ trajectories (and other anthropometric parameters) were estimated using restricted cubic regression spline models (Method 5 in the Supplementary Appendix). Per-protocol analyses were restricted to participants who consumed at least 90% of scheduled supplement doses and had no episodes of reported consumption of non-study vitamin D or calcium (Method 6 in the Supplementary Appendix). All analyses were performed using Stata version 13 (StataCorp, College Station, TX). 

## RESULTS

### Trial Population

**Figure 1: Trial flow diagram:**
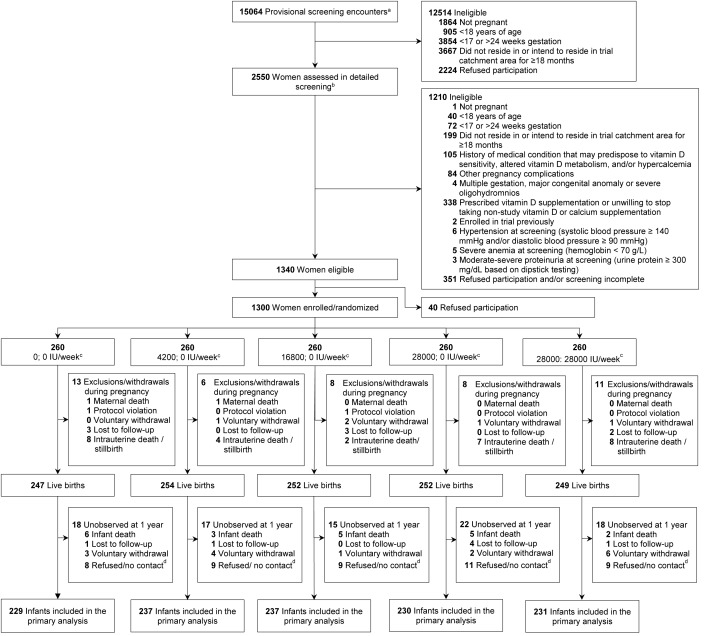
aProvisional screening refers to the initial eligibility assessment of pregnant women presenting for antenatal care at the Maternal and Child Health Training Institute; individual women may have been provisionally screened more than once during the trial enrolment period. 
bDetailed screening refers to the complete eligibility assessment supervised by a study physician.
cPrenatal; postpartum vitamin D dose.
dParticipants not formally lost to follow-up but for whom data at the 1-year visit were not obtained.

1,300 pregnant women were enrolled and randomized (Figure 1). Baseline characteristics including vitamin D status were similar across groups (Table 1; Table S5 and S6 in the Supplementary Appendix). Overall, 64% of women were vitamin D deficient. Groups did not differ by breast-feeding patterns or reported infant supplement use (Tables S7 and S8 in the Supplementary Appendix). Participants in primary analyses had higher average asset indices than those excluded but were otherwise similar (Table S9 in the Supplementary Appendix). Across all groups, ≥90% of scheduled doses were received by >90% of women in the prenatal period and >80% of women in postpartum periods (Table S10 in the Supplementary Appendix).

**Table 1:** Maternal characteristics at enrolment, by supplementation group

	Prenatal; Postpartum Vitamin D Dose (IU/Week)				
	0; 0	4200; 0	16800; 0	28000; 0	28000; 28000
Participants, N	259a	260	259a	260	260
Age, median (min, max)	23 (18, 38)	22.5 (18, 40)	22 (18, 35)	22 (18, 38)	23 (18, 38)
Gestational age (weeks), median (min, max)	20.4 (17, 24)	20.1 (17, 24)	20.3 (17, 24)	20.4 (17, 24)	20.1 (17, 24)
Married, n (%)b	255 (99.2)	259 (100)	254 (100)	257 (100)	256 (100)
Secondary schooling complete or higher, n (%)	52 (20.1)	70 (26.9)	51 (19.7)	58 (22.3)	55 (21.2)
Occupation outside the home, n (%)b	17 (6.6)	19 (7.3)	15 (5.9)	16 (6.2)	14 (5.5)
Asset index, median (min, max)c	-0.1 (-4.5, 4.1)	-0.2 (-3.2, 3.6)	0.0 (-4.5, 3.8)	-0.2 (-3.5, 4.9)	0.2 (-3.7, 4.5)
Gravidityd, median (min, max)	2 (1, 9)	2 (1, 6)	2 (1, 6)	2 (1, 7)	2 (1, 6)
Parity, median (min, max)	2 (0, 6)	2 (0, 5)	2 (0, 5)	2 (0, 5)	2 (0, 4)
Height (cm), mean ± SD	151.2 ± 5.4	150.9 ± 5.0	150.7 ± 5.5	150.2 ± 5.4	151.8 ± 5.5
Weight (kg), mean ± SD	54.5 ± 10.3	53.2 ± 10.1	53.8 ± 9.9	53.3 ± 9.1	55.2 ± 10.6
Serum 25(OH)D concentration (nmol/L), mean ± SDe	27.7 ± 13.8	27.4 ± 14.3	28.7 ± 14.0	27.0 ± 14.7	26.6 ± 13.2

a Participants found to be ineligible after randomization were excluded from analyses (one in each of the 0;0 and 16800;0 groups).

b N_0; 0_ = 257, N_4200; 0_ =259, N_16800; 0_ = 254, N_28000; 0_ = 257, N_28000; 28000_ = 256

c N_0; 0_ = 257, N_4200; 0_ = 258, N_16800; 0_ = 253, N_28000; 0_ = 256, N_28000; 28000_ = 256. Higher scores indicate greater household asset ownership relative to other participants. See Method 7 in the Supplementary Appendix for a description of the construction and interpretation of the asset index.

d Number of pregnancies, including the current pregnancy.

e N_0; 0_ = 253, N_4200; 0_ = 258, N_16800; 0_ = 258, N_28000; 0_ = 258, N_28000; 28000_ = 256. Vitamin D deficiency defined as 25(OH)D <30 nmol/L.

### Infant Growth Outcomes

Infant follow-up at 1 year of age was completed for 90% of pregnancies and 94% of infants alive at 1 year (Figure 1). Overall, mean LAZ at 1 year was -1.00 (SD 1.04) and the prevalence of stunting was 16%. Prenatal or postpartum maternal vitamin D supplementation did not increase or decrease infant length or other anthropometric outcomes by one year of age (Table 2; Figures S1-S5 and Tables S11 and S12 in the Supplementary Appendix). The lack of effect of prenatal vitamin D on length was evident from birth (Table 3) and was supported by sensitivity and stratified analyses (Tables S13-S22 in the Supplementary Appendix). Results of the multiple imputation analysis agreed closely with the complete case analysis (Table S18 in the Supplementary Appendix); therefore, the complete case analysis is shown in Table 2.

**Table 2:** Anthropometric outcomes of infants at 1 year of age, by supplementation group

	Prenatal; Postpartum Vitamin D Dose (IU/Week)					
	0; 0	4200; 0	16800; 0	28000; 0	28000; 28000	pa
Age at measurement (days), median (min, max)	364 (364, 419)	365 (364, 415)	365 (364, 418)	365 (364, 419)	365 (364, 416)	0.70
**Length**						
N	229	237	237	230	231	-
Length (cm), mean ± SD	72.62 ± 2.76	72.31 ± 2.84	72.56 ± 2.54	72.39 ± 2.80	72.67 ± 2.53	0.53
Length-for-age z-score, mean ± SD	-0.93 ± 1.05	-1.11 ± 1.12	-0.97 ± 0.97	-1.06 ± 1.07	-0.94 ± 1.00	0.23
Stuntedb, n (%)	36 (15.7)	46 (19.4)	36 (15.2)	39 (17.0)	31 (13.4)	0.49
**Other Anthropometric Indices**						
Weight-for-age z-score, mean ± SDc	-0.81 ± 1.12	-1.00 ± 1.14	-0.86 ± 1.09	-0.96 ± 1.09	-0.89 ± 1.04	0.34
Weight-for-length z-score, mean ± SDc	-0.47 ± 1.07	-0.60 ± 1.03	-0.52 ± 1.08	-0.58 ± 1.01	-0.59 ± 1.01	0.62
Body mass index-for-age z-score, mean ± SDc	-0.36 ± 1.05	-0.48 ± 1.00	-0.40 ± 1.07	-0.46 ± 0.99	-0.48 ± 1.00	0.66
Head circumference-for-age z-score, mean ± SDd	-1.11 ± 0.99	-1.25 ± 0.96	-1.21 ± 1.05	-1.22 ± 0.92	-1.22 ± 0.89	0.61
Mid-upper arm circumference-for-age z-score, mean ± SDe	-0.14 ± 0.97	-0.27 ± 0.92	-0.21 ± 0.93	-0.29 ± 0.88	-0.23 ± 0.86	0.42
Wastedf, n (%)c	14 (6.1)	22 (9.3)	18 (7.6)	18 (7.9)	21 (9.1)	0.72

a P-values for multiple group comparisons are from ANOVA or Kruskal-Wallis tests for continuous variables, and Chi-square tests for categorical variables.

b Length-for-age z-score <-2.

c N0; 0 = 228, N 4200; 0 = 236, N 16800; 0 = 237, N 28000; 0 =229, N 28000; 28000 = 231

d N0; 0 = 225, N 4200; 0 = 235, N 16800; 0 = 234, N 28000; 0 =229, N 28000; 28000 = 231

e Same sample size as length-for-age z-score.

f Weight-for-length z-score <-2.

**Table 3:** Delivery characteristics and pregnancy outcomes, by supplementation group

	Prenatal; Postpartum Vitamin D Dose (IU/Week)					
	0; 0	4200; 0	16800; 0	28000; 0	28000; 28000	pb
Characteristic/Outcomea	N = 259	N = 260	N = 259	N = 260	N = 260	
Live birth, n (%)	247 (95.4)	254 (97.7)	252 (97.3)	252 (96.9)	249 (95.8)	0.53
Gestational age at birth (weeks), median (min, max)	39.1 (32, 43)	39.1 (34, 42)	39.0 (26, 43)	39.1 (29, 43)	39.1 (30, 42)	0.62
Preterm (<37 weeks), n (%)	24 (9.7)	21 (8.3)	31 (12.3)	26 (10.3)	22 (8.8)	0.60
Caesarean section, n (%)	121 (49.0)	143 (56.3)	131 (52.0)	127 (50.4)	132 (53.0)	0.54
Facility (hospital or clinic) deliveryc, n (%)	211 (85.4)	216 (85.0)	216 (85.7)	212 (84.1)	207 (83.1)	0.93
Female infant, n (%)	129 (52.2)	117 (46.1)	132 (52.4)	124 (49.2)	121 (48.6)	0.58
Maternal serum 25(OH)D concentration at/near delivery (nmol/L), mean ± SDd	25.4 ± 21.0	69.0 ± 19.4	99.7 ± 23.7	110.0 ± 27.6	112.2 ± 28.8	<0.001e
Newborn anthropometryf, mean ± SD						
Birth weight (kg)g	2.72 ± 0.36	2.70 ± 0.39	2.72 ± 0.35	2.67 ± 0.34	2.76 ± 0.35	0.25
Length at birth (cm)h	47.4 ± 2.1	47.5 ± 1.9	47.4 ± 1.9	47.2 ± 2.1	47.5 ± 2.0	0.74
Head circumference at birth (cm)i	33.0 ± 1.3	33.0 ± 1.3	33.0 ± 1.1	32.9 ± 1.2	33.0 ± 1.1	0.73
Gestational age/sex-standardized growth parameterf, mean ± SD						
Weight-for-age z-score at birthg	-1.12 ± 0.83	-1.27 ± 0.89	-1.15 ± 0.90	-1.30 ± 0.82	-1.12 ± 0.85	0.16
Length-for-age z-score at birthh	-0.83 ± 1.04	-0.95 ± 1.00	-0.90 ± 1.05	-1.00 ± 1.02	-0.88 ± 0.95	0.61
Head circumference-for-age z-score at birthi	-0.58 ± 0.96	-0.66 ± 1.04	-0.57 ± 0.94	-0.72 ± 0.98	-0.58 ± 0.91	0.57
Low birth weightf,j, n (%)g	42 (25.3)	53 (31.0)	42 (25.0)	53 (32.9)	40 (23.7)	0.23
Small for gestational agef, k, n (%)g	72 (43.4)	88 (51.5)	77 (45.8)	84 (52.2)	76 (45.0)	0.38

a Except for maternal serum 25(OH)D concentration at/near delivery, all characteristics and outcomes presented are among live births only.

b P-values from ANOVA or Kruskal-Wallis tests for continuous variables, and Chi-square or Fischer’s tests for categorical variables.

c Two infants (in group 0; 0 and 16800; 0) were born at a location other than a hospital/clinic or home; all other deliveries were home births.

d N0; 0 = 125, N4200; 0 = 119, N16800; 0 = 127, N28000; 0 = 113, N28000; 28000 = 124. Median (IQR) gestational age (days) at timing of measurements was 275 (268-281) days.

e Post-hoc pairwise comparisons using t-tests showed significant pairwise differences between all groups except both groups who received a prenatal dose of 28000 IU/week, after adjusting for multiple comparisons using the Holm test.

f Limited to measurements obtained within 48 hours of birth.

g N0; 0 = 166, N4200; 0 = 171, N16800; 0 = 168, N28000; 0 =161, N28000; 28000 = 169

h N0; 0 = 164, N4200; 0 = 169, N16800; 0 = 166, N28000; 0 = 160, N28000; 28000 = 165

i N0; 0 = 167, N4200; 0 = 168, N16800; 0 = 169, N28000; 0 = 159, N28000; 28000 = 167

j Weight <2500 g.

k Weight-for-age z-score below the 10th percentile, based on the Intergrowth-21st Neonatal Standards.

### Biochemical effects of supplementation

**Figure 2: Study design and CONSORT diagram:**
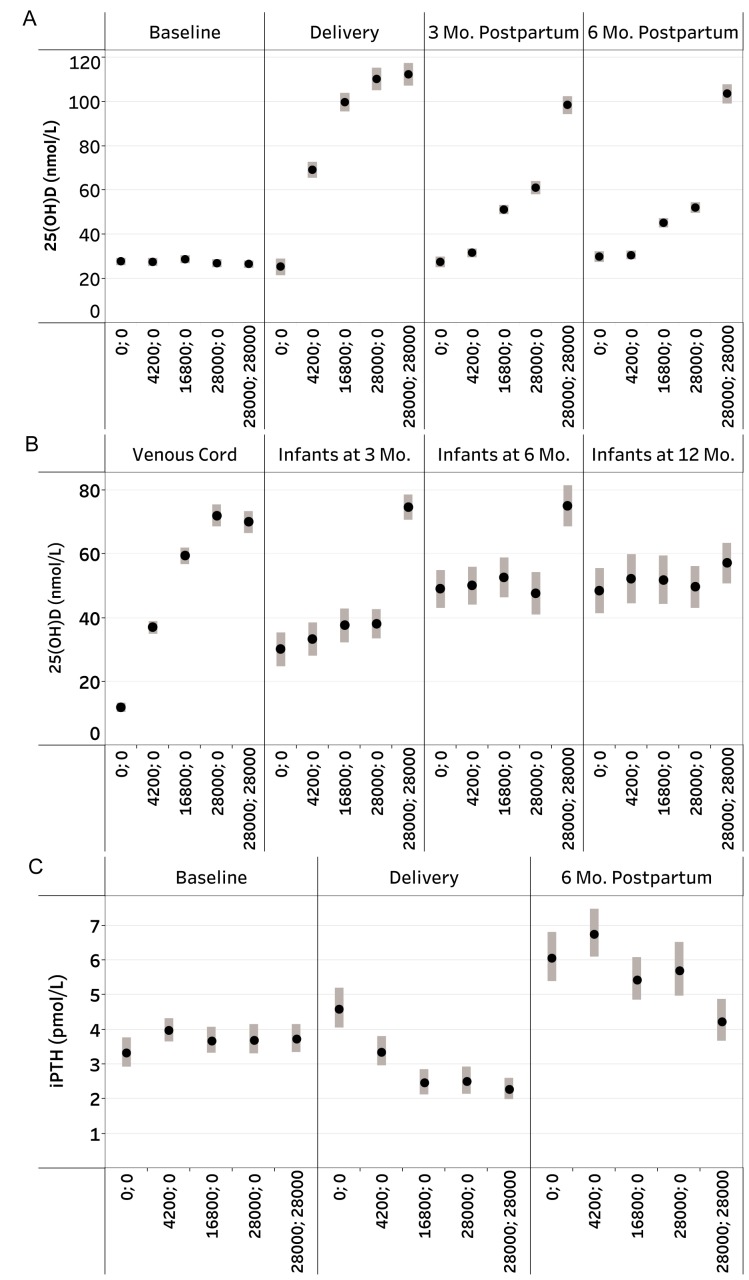
Maternal, venous cord, and infant 25-hydroxyvitamin D (25(OH)D) [nmol/L], and maternal iPTH [pmol/L] concentrations, by vitamin D supplementation group. (A) Mean maternal 25(OH)D at baseline (n=1285), delivery (n=656; delivery specimens were collected within -19 days to 11 days of delivery [median: 0 days]), 3 months postpartum (n=581), and 6 months postpartum (n=590). Bars denote 95% confidence intervals (CI); (B) Mean 25(OH)D concentrations in venous cord (n=507), infants at 3 months (n=345), infants at 6 months (n=254), and infants at 12 months (n=182). Bars denote 95% CI; (C) Geometric means of maternal iPTH concentrations [pmol/L] at baseline (n=608), delivery (n=551; delivery specimens were collected within -19 days to 11 days of delivery [median: 0 days]), and 6 months postpartum (n=588). Bars denote 95% CI.

Vitamin D had dose-dependent effects on maternal, cord blood and infant 25(OH)D (Table 3; Figure 2; Tables S23 and S24 in the Supplementary Appendix) and maternal iPTH at delivery; the 28000;28000 group continued to have significantly lower iPTH at 6-months postpartum versus other groups (Figure 2; Table S25 in the Supplementary Appendix).

### Safety outcomes

Episodes of confirmed hypercalcemia (all asymptomatic) occurred in 0 women in the prenatal period, 8 women (0.7%) postpartum (5 in the 28000;28000 group), and 6 infants (0.7%); however, the frequencies of maternal postpartum or infant confirmed or possible hypercalcemia did not differ significantly across groups (Table S25 in the Supplementary Appendix). Prenatal vitamin D supplementation modestly elevated maternal and fetal/infant mean sCa; differences were most pronounced for the 16800;0, 28000;0, and 28000;28000 versus 0;0 comparisons up to 3-months postpartum (Table S25, S26 in the Supplementary Appendix). At 6-months postpartum, groups were similar with respect to both maternal and infant sCa (Table S25 in the Supplementary Appendix), but the 28000;28000 group had higher maternal sCa versus 0;0 or 28000;0 in post-hoc comparisons (Table S26 in the Supplementary Appendix). Maternal uCa:Cr at delivery varied significantly across groups (lowest median value in the 0;0 group) and the risk of maternal possible hypercalciuria at delivery increased in a dose-dependent manner; however, only the 28000;28000 group differed significantly from 0;0 in pairwise comparisons after correcting for multiple testing (Table S25 in the Supplementary Appendix). There were two asymptomatic cases of maternal confirmed hypercalciuria, one each in the 0;0 and 28000;0 groups (Table S25 in the Supplementary Appendix), but no women with urinary tract stones (Table S27 in the Supplementary Appendix). None of the women with confirmed hypercalcemia or confirmed hypercalciuria experienced serious adverse events (hospitalizations or deaths). Two of the six infants with confirmed hypercalcemia had neonatal hospitalizations for acute illnesses, but these events were temporally and clinically unrelated to the later findings of asymptomatic hypercalcemia. There was one infant with confirmed hypercalciuria (4200;0 group) and no differences across groups in infant uCa:Cr at 6 months of age (Table S25 in the Supplementary Appendix). 

### Secondary Clinical Outcomes

Delivery characteristics, duration of gestation, preterm birth, SGA, and LBW were similar across groups (Table 3 and Table S28 in the Supplementary Appendix). There was no consistent evidence of beneficial or harmful effects of any vitamin D dose on maternal or infant morbidity (Tables S27, S29-S31 in the Supplementary Appendix), gestational hypertension (Tables S27 in the Supplementary Appendix) or maternal self-reported or caregiver-reported infant symptoms (Tables S32 and S33 in the Supplementary Appendix). Stillbirth (Table S28 in the Supplementary Appendix) and infant death rates (Table S27 in the Supplementary Appendix) did not differ significantly across groups. Of four infants with radiologically-confirmed rickets, three were in 0;0 and one was in the 4200;0 group (Table S27 in the Supplementary Appendix).

## DISCUSSION

In a setting of widespread vitamin D deficiency and fetal-infant growth restriction, vitamin D supplementation from mid-pregnancy to delivery or 6-months postpartum is safe but does not influence offspring growth patterns, and has no discernible effects on numerous pregnancy or infant clinical outcomes.

These findings do not support the hypothesis that prenatal vitamin D status in the 2nd half of pregnancy is a determinant of newborn size, contrary to the conclusions of prior meta-analyses of observational studies^12^ and mostly small trials^13^, but consistent with higher-quality trials in settings with lower prevalence of vitamin D deficiency or fetal growth restriction^26-29^. Our earlier trial in Bangladesh^14^ and a study in the United Kingdom^30^ found that prenatal vitamin D increased infant linear growth. However, they were small studies (n<135) with postnatal growth as a post-hoc outcome, and the between-group differences may have been due to chance. The present findings are broadly consistent with a meta-analysis of six trials of prenatal daily multiple micronutrient supplements (200 to 400 IU vitamin D) in low- and middle-income countries, which showed no effect on height at 2-8.5 years of age^3^. 

There was no clear evidence of other health benefits of improved vitamin D status in the latter half of pregnancy or early infancy. In particular, purported effects of vitamin D on preterm birth^31^ were not substantiated, consistent with a recent meta-analysis^13^. Published prenatal vitamin D trials in high-income countries have been limited by the inclusion of few (if any) women with vitamin D deficiency and the lack of a placebo (no vitamin D) group^26,27,32^. MDIG had several advantages: most participants were vitamin D deficient at enrolment; true placebo control group; excellent adherence; robust dose-response effect on vitamin D status across a wide dose range up to the tolerable upper intake level (4000 IU/day); rigorous anthropometric and clinical data collection; and, high retention rates.

The surprising lack of any demonstrable adverse effects of maternal vitamin D deficiency on infant outcomes underscored the uncertainties about vitamin D requirements in pregnancy and lactation. The dose equivalent to the Institute of Medicine recommended dietary allowance^24^ (4200 IU/week) was sufficient for eliminating maternal vitamin D deficiency (25(OH)D<30 nmol/L) without elevating 25(OH)D above a conservative long-term risk threshold (125 nmol/L)^24^. However, 16800 IU/week prevented maternal vitamin D deficiency at the <50 nmol/L cut-off, maximally suppressed maternal iPTH, and prevented cord 25(OH)D<30 nmol/L. Maternal postpartum supplementation (28000 IU/week) maintained infant 25(OH)D above 30 nmol/L up to 6 months of age, despite variability in feeding patterns. The occurrence of four cases of rickets in the placebo and 4200;0 groups was consistent with a plausible effect of 16800 IU/week or higher prenatally in the prevention of early rickets, but the incidence of x- ray-confirmed rickets was too low to be conclusive. Ongoing analyses of infant acute respiratory infections^33^ and other proposed follow-up studies of the MDIG cohort may provide additional insights into longer-term effects of maternal vitamin D supplementation on child health (e.g., asthma^27,32^, bone mass^26^). 

Although active clinical surveillance in the MDIG cohort did not reveal effects of vitamin D on maternal health measures including gestational hypertension, earlier initiation of supplementation and a larger sample size may be required to assess effects on hypertensive disorders of pregnancy or gestational diabetes. Some effects of vitamin D may have been masked by concomitant supplementation with calcium or other untreated nutrient deficiencies. Also, linear growth faltering was milder in participants than in national surveys^15^ suggesting that participants had relatively better baseline health and access to care. For example, participants had high rates of facility deliveries (85%, versus 37% nationally) and C-sections (51%, which is typical of local health facilities but more than double the national average)^15^. Consistent with WHO recommendations^34^, MDIG trial findings do not support routine vitamin D supplementation in pregnancy or lactation to improve birth outcomes or infant growth, even in communities with endemic vitamin D deficiency and fetal-infant growth restriction. 

## Supplementary Appendix

Supplementary Material
