# Supplementary material for: Vitamin D supplementation in pregnancy and lactation to promote infant growth
Source: N Engl J Med. Author manuscript; Available in PMC 2018 Aug 9. (PMC6004541; doi:10.1056/NEJMoa1800927)
Supplement: Supplementary Material [file NEJMoa1800927_Roth_Supplement.pdf]

Supplementary Appendix

Vitamin D supplementation in pregnancy and lactation to promote infant growth

Daniel E. Roth, M.D., Ph.D., Shaun K. Morris, M.D., Stanley Zlotkin, M.D., Ph.D., Alison D. Gernand, Ph.D., Tahmeed Ahmed, MBBS, Ph.D., Shaila Sharmeen Shanta, MBBS, Eszter Papp, Ph.D., Jill Korsiak, M.Sc., Joy Shi, M.Sc., M. Munirul Islam, MBBS, Ph.D., Ishrat Jahan, MBBS, MPH, Farhana Khanam Keya, MBBS, Andrew R. Willan, Ph.D., Rosanna Weksberg, M.D., Minhazul Mohsin, MBBS, Qazi Sadeq-ur Rahman, M.Sc., Prakesh S. Shah, M.D., Kellie E. Murphy, M.D., Jennifer Stimec, M.D., Lisa G. Pell, Ph.D., Huma Qamar, B.Sc., Abdullah Al Mahmud, MSS, MMSc.

Daniel Roth
Hospital for Sick Children
686 Bay Street, Toronto, Canada
daniel.roth@sickkids.ca

Contents

Additional Methods 1

Method 1. Detection of outliers and implausible anthropometric values 1

Data sources 1

Operational definitions 1

Statistical analysis 1

Method 2. Measurements of serum 25-hydroxyvitamin D concentrations 2

Description of assay 2

Specific details on lab techniques 2

Quality control 3

Statistical analysis 3

Method 3. Rickets screening and determination 4

Method 4. Sensitivity and stratified analyses 5

Data sources 5

Operational definitions 5

Statistical analysis 6

Method 5. Graphical representation of longitudinal anthropometric data 8

Method 6. Determination of supplementation adherence 9

Data sources 9

Operational definitions 9

Statistical analysis 10

Method 7. Development of asset index 11

Data source 11

Operational definition 11

Statistical analysis 11

Figures 12
Figure S1. Length-for-age z-scores from birth to 1 year of age 12
Figure S2. Weight-for-age z-score from birth to 1 year of age 13
Figure S3. Weight-for-length z-score from birth to 1 year of age 14
Figure S4. Body mass index-for-age z-score from birth to 1 year of age 15
Figure S5. Head circumference-for-age z-score from birth to 1 year of age 16
Tables 17
Table S1. Participant inclusion and exclusion criteria 17
Table S2. MDIG trial data collection procedures from enrolment to 1 year postpartum 18
Table S3. Quality control of anthropometric measures at each scheduled measurement time point 22
Table S4. List of secondary outcomes 24
Table S5. Additional maternal characteristics at enrolment, by supplementation group 27
Table S6. Maternal characteristics at enrolment for participants included in the complete case analysis, by supplementation group 28
Table S7. Infant feeding practices among participants included in the complete-case analysis during the first 6 months of age, by supplementation group 29
Table S8. Infant vitamin D consumption from birth to 1 year of age 30
Table S9. Comparison of maternal characteristics at enrolment between participants included in the complete case analysis and participants excluded from the complete case analysis 31
Table S10. Supplementation duration and adherence among participants included in the complete case analysis, by supplementation group 32
Table S11. Raw anthropometric parameters and additional anthropometric indicators for infants at 1 year of age, by supplementation group 33
Table S12. Pairwise between-group comparisons of the effect of different doses of prenatal and postpartum vitamin D supplementation on length-for-age z-score, weight-for-age z-score, weight-for-length z-score, body mass index-for-age z-score, head-circumference-for-age z-score, and mid-upper arm circumference-for-age z-score at 1 year of age 34
Table S13. Anthropometric outcomes of infants at 1 year of age by supplementation group, after correcting for gestational age at birth 36
Table S14. Per-protocol analysis of anthropometric outcomes of infants at 1 year of age, by supplementation group 37
Table S15. Length-for-age z-scores of infants at 1 year of age by supplementation group and stratified by gestational age at birth, infant sex, maternal height, maternal supplement adherence, or maternal baseline vitamin D status 38

Table S16. Overall differences in length-for-age z-score at 1 year of age across supplementation groups after adjustment for individual baseline characteristics 39
Table S17. Marginal effect of treatment allocation on length-for-age z-scores at 3-month time intervals from birth to 1 year of age 40
Table S18. Length-for-age z-scores of infants at 1 year of age by supplementation group, using multiple imputation to impute missing 1 year length-for-age z-scores 41
Table S19. Effect of prenatal and postpartum vitamin D on anthropometric outcomes at 1 year of age after correcting for gestational age at birth 42
Table S20. Per-protocol analysis of the effect of prenatal and postpartum vitamin D supplementation on anthropometric outcomes at 1 year of age. 43
Table S21. Effect of prenatal and postpartum vitamin D on length-for-age z-score at 1 year of age by gestational age at birth, infant sex, maternal height, maternal supplement adherence, and maternal baseline serum 25(OH)D concentration 45
Table S22. Effect of prenatal and postpartum vitamin D on length-for-age z-score at 1 year of age after adjustment for a single baseline characteristic 47
Table S23. Maternal, venous cord and infant 25-hydroxyvitamin D concentrations, by supplementation group 48
Table S24. Maternal, venous cord and infant C-3 epimer of 25-hydroxyvitamin D3 and total 25(OH)D3, by supplementation group 50
Table S25. Maternal and infant biochemical measures and adverse events, by supplementation group 52
Table S26. Serum calcium adjusted for differences at baseline or in cord blood for women and infants, respectively 54
Table S27. Clinical encounters and hospitalizations among women and infants, by supplementation group 56
Table S28. Additional delivery characteristics and pregnancy outcomes, by supplementation group 58
Table S29. Primary diagnoses at clinical encounters and primary discharge diagnoses for hospitalizations among women up to 6 months postpartum 59
Table S30. Primary diagnoses at clinical encounters and primary discharge diagnoses for hospitalizations among infants up to 6 months of age 60
Table S31. Primary diagnoses at clinical encounters and primary discharge diagnoses for hospitalizations among infants from 6-12 months of age 62
Table S32. Frequencies of reported symptoms among women based on weekly clinical monitoring during the prenatal and 6 month postpartum periods 64
Table S33. Frequencies of reported symptoms among infants during the neonatal (≤ 4 weeks of age) and postneonatal (>4 weeks of age) periods based on weekly clinical monitoring during the first 6 months of age 69
References 72

Additional Methods

Method 1. Detection of outliers and implausible anthropometric values

Data sources

Infant length, weight, head circumference, upper-arm length, mid-upper arm circumference, and rump-knee length measurements collected on a tri-monthly basis from birth until 2 years of age, with an additional measurement between 2-8 weeks of age.

Operational definitions

Outliers: (1) Biologically implausible z-scores based on the World Health Organization Anthro software (<-6 SD or >6 SD for LAZ; >5 SD or <-6 SD for WAZ; >5 SD or <-5 SD for HCAZ, WFL and MUACAZ); (2) Aberrations from individual growth trajectories identified through a 'residual method'[1], described briefly below.

Implausible values: Anthropometric measurements that were temporally inconsistent with previous measurements. Length, head circumference, upper-arm length and rump-knee length were assumed to be constantly increasing through serial measurements, while weight may fluctuate slightly between successive measurements. Any decreases in length, head circumference, upper-arm length and rump-knee length were identified as implausible measurements, whereas any declines in weight ≥10% between two successive measurements were considered implausible.

Statistical analysis

The purpose of identifying outlier was to prompt manual data review and reconciliation. Values were corrected if the review of data forms and study logs provided information that enabled such corrections. However, outliers that could not be reconciled remained in all analyses unless there was sufficient evidence to indicate they were a result of measurement or data entry error.

To identify outliers based on the residual method[1], linear regression was used to fit a straight line through an individual's age-standardized z-score (Z) as a function of age (t): $Z_{ij} = \beta_{0i} + \beta_i t_{ij} + \varepsilon_{ij}$; where "*i*" is the ith individual and "*j*" reflects the jth time point. Based on the model predicted for each individual, jackknife residuals were calculated, and measurements with an absolute jackknife residual ≥5 were flagged as outliers for length, weight, and head circumference, and measurements with an absolute jackknife residual ≥7 were flagged as outliers for mid-upper arm circumference. A similar procedure was applied to raw anthropometric data when z-scores were not available (rump-knee length, upper-arm length), except the raw anthropometric measurement (Y) was regressed on the square root of age ($t^{1/2}$): $Y_{ij} = \beta_{0i} + \beta_i t_{ij}^{½} + \varepsilon_{ij}$; where "*i*" reflects the ith individual and "*j*" reflects the jth time point. Based on the model predicted for each individual, jackknife residuals were calculated and measurements with an absolute jackknife residual ≥7 were flagged as outliers.

When implausible values due to temporal inconsistencies between two consecutive measurements were flagged, jackknife residuals for each of the two time points were compared and the measurement with the largest absolute jackknife residual was considered the incorrect measurement. Implausible values were dropped from analysis unless they could be reconciled and corrected through manual review.

Method 2. Measurements of serum 25-hydroxyvitamin D concentrations

Description of assay

Maternal (baseline, delivery, 3 months postpartum, 6 months postpartum), venous cord, and infant (3 months, 6 months, 12 months) 25-hydroxyvitamin D (25(OH)D) concentrations were measured using high performance liquid chromatography- tandem mass spectrometry (LC-MS/MS) following methodology previously described[2,3]. In addition to the advantage of high specificity and sensitivity of LC-MS/MS, the specific methodology employed allowed for separate quantification of 25(OH)D3, 25(OH)D2, and 25(OH)D3 C-3 epimer concentrations[2,3].

All serum 25(OH)D measurements were conducted at the Analytical Facility for Bioactive Molecules (AFBM) at the Hospital for Sick Children (Toronto, Canada). The AFBM participates in Vitamin D External Quality Assessment Scheme (DEQAS) certification.

Specific details on lab techniques

HPLC grade hexanes and methanol were purchased from Caledon Laboratories Ltd. (Georgetown, ON). Standards were obtained from Cerilliant Corporation (Round Rock, TX), and Sigma Aldrich (St. Louis, MO).

Serum samples of women at baseline, delivery, 3 months postpartum and 6 months postpartum; venous cord; and infants at 3 months, 6 months and 12 months of age were extracted by the AFBM via liquid-liquid using a Zinc Sulfate/Methanol/n-Hexane protocol. Standard curves were prepared in 4% BSA in PBS and the internal standard 25(OH) Vitamin D3-d6 was prepared in methanol.

Internal standard was added to silanized glass round-bottom tubes. Sample, standard, or NIST SRM972a quality control was then added, followed by 0.4M $ZnSo_4$.

After a brief vortex, methanol was added and tubes were vortexed again. Next, n-Hexanes were added and tubes were vortexed for 1 minute followed by a 30 second rest. The tubes were vortexed for 1 minute with 30 second rest a total of 3 times. Samples were then centrifuged at 800g for 10 minutes. Hexane supernatants were transferred to a clean conical silanized glass tube. Methanol was added to the original tubes and samples were vortexed thoroughly before a second addition of n-Hexanes. Samples were then vortexed and centrifuged as described above, and the supernatant was combined with the previously transferred hexane supernatant. Samples were taken to dryness under nitrogen and then reconstituted in equal parts water and methanol and transferred into inserts in autosampler vials.

Samples were analyzed by LC-MS/MS using an Aglient 1290 HPLC interfaced with an AB Sciex 5500 Q-Trap mass spectrometer. Samples were injected onto a Kinetex F5 2.6 um 100 x 3.0 mm column (Phenomenex). Chromatographic separation of 25(OH)D2, 25(OH)D3, and the 25(OH)D3 C-3 epimer occurred at retention times of 2.55, 2.40, and 2.54 min, respectively. Analytes were eluted from the Kinetex column at a flow rate of 700 µL/min over a gradient of mobile phases of water and methanol.

Data was acquired with a QTRAP 5500 triple-quadruple mass spectrometer (Sciex: Framingham, Massachusetts, USA) in positive APCI mode by MRM data acquisition. Transitions monitored for 25(OH)D2, 25(OH)D3, and the C3 epimer of 25(OH)D3 were 413.33/91 m/z, 401.32/365.3 m/z and 401.40/365.25 m/z, respectively.

Data were collected and analyzed using Sciex Analyst v1.6.3.

Quality control

To ensure quality of data, NISTSRM972a quality control and DEQAS standards (451, 452, 453, 454, 455) were run with every batch of specimens assayed. DEQAS standards were regularly monitored for accuracy by comparing calculated concentrations with the expected concentrations. In addition, a pooled plasma sample was run in at least duplicate with every batch assayed. Average inter-assay coefficient of variation (CV) was 10%, and average intra-assay CV was 5%.

Statistical analysis

Distributions of maternal serum 25(OH)D concentrations (nmol/L) at baseline, delivery, 3 months postpartum and 6 months postpartum; serum 25(OH)D concentration in cord blood; and infant serum 25(OH)D concentrations at 3 months, 6 months and 12 months of age, stratified by treatment group, were first visualized using boxplots and jittered scatterplots. In addition, graphs of mean 25(OH)D concentrations with 95% confidence interval bands were generated for each of the specified maternal and infant time points, stratified by treatment arm.

Means and standard deviations of maternal serum 25(OH)D concentrations (nmol/L) at baseline, delivery, 3 months postpartum and 6 months postpartum; serum 25(OH)D concentrations in venous cord blood; and infant serum 25(OH)D concentrations at 3 months, 6 months and 12 months of age, were calculated, stratified by treatment arm (Table S23). Comparisons of 25(OH)D concentrations across treatment groups at each of the specified time points were conducted using ANOVA. For those ANOVAs that were statistically significant ($p<0.05$), pairwise comparisons using t-tests were carried out between each of the treatment groups. The Holm test was used to account for multiple testing.

The number and proportion of women and infants at each of the specified time points with measured 25(OH)D concentrations <30 nmol/L; 25(OH)D concentrations ≥30 nmol/L and ≤50 nmol/L, and >125 nmol/L were also calculated, by treatment group (Table S23). Comparisons across treatment arms at each time point were carried out using Chi-square or Fischer's exact tests. For those tests that were statistically significant, pairwise comparisons were carried out between each of the treatment groups, adjusting for multiple comparisons by using the Holm test.

Primary analyses of 25(OH)D concentrations excluded the C-3 epimer of 25(OH)D3. Since 25(OH)D2 concentrations were undetectable or negligible in a large subset (n=1149) of representative samples that we checked, reported 25(OH)D values represent 25(OH)D3 only (Table S23). Concentrations of the C-3 epimer of 25(OH)D3, as well as total 25(OH)D concentrations (sum of 25(OH)D3 and C-3 epimer of 25(OH)D3) were reported separately as means with standard deviations, and proportions of women and infants at each specified time point, by treatment group (Table S24).

Method 3. Rickets screening and determination

Infants were scheduled for screening for rickets at 6-month clinical visits. Serum calcium, inorganic phosphorous, and alkaline phosphorous (ALP) were measured in venous blood, and results were reported to the study physician. X-rays of wrists and/or knees, additional blood work, and referral to a pediatrician for assessment and treatment were prompted if any of the following criteria were met: ALP>450 U/L; or, ALP>350 U/L and phosphorus <1.56 mmol/L; or, phosphorus <1.00 mmol/L; or, total calcium < 2.10 mmol/L. If infant blood samples were not collected at the 6-month clinical visit, blood collection for rickets screening was offered at a later date. Wrist and/or knee radiographs were interpreted by a pediatric radiologist (J.S.) blinded to clinical and laboratory data to determine the number of infants with x-ray-confirmed rickets.

Method 4. Sensitivity and stratified analyses

Sensitivity analyses included: (1) using z-scores assigned based on gestational age (GA)-corrected age instead of chronological age; (2) per-protocol analyses; (3) subgroup and interaction analyses; (4) adjustment for baseline characteristics; (5) marginal effects of supplementation on length-for-age z-score (LAZ) from birth to 1 year; (6) multiple imputation to impute missing length-for-age z-scores of infants at 1 year of age.

Data sources

Gestational age at birth was based on recalled last menstrual period and 2nd trimester ultrasound reports (see Table S1 in the Supplementary Appendix). Per-protocol analyses were based on adherence data, described in detail in Method 6 of the Supplementary Appendix. Data for subgroup/interaction and adjusted analyses were captured in study questionnaires from baseline and delivery, maternal anthropometry and biochemical measurements (25(OH)D, hemoglobin). Data for the marginal effects analyses used infant LAZ measurements from birth until 1 year of age. In addition to treatment allocation and length-for-age z-score at 1 year of age, infant anthropometric measurements at 6 and 9 months of age, maternal baseline characteristics (asset index, height) and infant sex were used for multiple imputation.

Operational definitions

GA-corrected age: GA-correction was only applied to preterm infants (<37 weeks), and was calculated using the following formula: GA-corrected age = Chronological age – (280 – Gestational age at birth), where gestational age at birth was the difference in days between the date of last menstrual period (LMP) and infant date of birth.

Per-protocol analyses: Per-protocol analyses were restricted to participants who received at least: (1) 90% of all scheduled study supplement doses and (2) had no consumption of non-study vitamin D or calcium from enrolment to 6-months postpartum for the overall test of difference across treatment groups.

For the five pairwise comparisons that assessed the prenatal effect of vitamin D supplementation (4200; 0 vs. 0; 0, 16800; 0 vs. 0; 0, 16800; 0 vs. 4200; 0, 28000; 0 vs. 0; 0, 28000; 0 vs. 16800; 0), per-protocol was considered to be consumption of at least 90% of prenatal doses and no consumption of non-study vitamin D or calcium in the prenatal period.

For the single pairwise comparison that assessed the effect of postpartum supplementation (28000; 28000 vs. 28000; 0), per-protocol was consumption of at least 90% of postpartum doses and no consumption of non-study vitamin D or calcium in the 0-6 month postpartum period.

Subgroup/interaction analyses:
The following variables were used to define subgroups: (1) gestational age at birth, dichotomized as preterm (<37 weeks gestational age) and term (≥37 weeks gestational age); (2) infant sex (male / female); (3) maternal baseline serum 25(OH)D concentration, dichotomized using two different cut-offs (<30 nmol/L and ≥30 nmol/L, and <20 nmol/L and ≥20 nmol/L); (4) maternal height, dichotomized at the median (<151 cm or ≥151 cm); (5) maternal supplement adherence, dichotomized as those who were per-protocol and not per-protocol. All subgroup analyses were pre-specified in the study protocol. Specific cut-offs for preterm birth, maternal baseline serum 25(OH)D concentrations (30 nmol/L and ≥30 nmol/L), and maternal height were decided before unmasking. Maternal 25(OH)D concentrations using the cut-off of <20 nmol/L and ≥20 nmol/L was decided post-hoc, after unmasking.

Statistical analysis

(1): GA-corrected age analyses: Among preterm infants, z-scores were assigned based on gestational age-corrected age, whereas for term infants z-scores were based on chronological age. The global test of difference across treatment groups using an ANOVA and the 6 pairwise comparisons that assessed the prenatal and postpartum effects of vitamin D supplementation on infant growth using t-tests (with adjustment for multiple comparisons using the Holm test in the prenatal period) were repeated using gestational-age corrected z-scores.

(2) Per-protocol analyses: The global test of difference across treatment groups using an ANOVA was repeated among participants classified as overall per-protocol. Five pairwise comparisons that assessed the effect of prenatal supplementation were repeated among participants classified as per-protocol in the prenatal period using t-tests and the Holm test to adjust for multiple comparisons. One pairwise comparison to assess the effect of postpartum supplementation was repeated among participants classified as per-protocol in the postpartum period using a t-test.

(3) Subgroup/interaction analyses: To test the overall effect of treatment assignment on LAZ at 1 year of age within each subgroup, ANOVA was used. A linear regression model with an interaction term between each treatment group and the respective subgroup was also generated, and contrasts were performed on the interaction term to assess the overall significance of this interaction effect across all treatment groups.

(4) Adjustment for baseline covariates: To adjust for baseline characteristics, separate linear regression models were used to assess the change in the primary inferences upon adjustment for each baseline characteristic. The coefficient estimate (and 95% CI) and the significance of the coefficient was presented for each covariate. Contrasts were conducted to test the difference in LAZ at 1-year of age across treatment groups after adjusting for the covariate included in the model. Two p-values for each covariate were presented: $p_{covariate}$ which reflects the association between LAZ at 1 year and the covariate of interest, and p_{group} which reflects the overall difference in LAZ at 1 year of age after adjusting for the single baseline characteristic.

(5) Marginal effects of supplementation on length-for-age z-score from birth to 1 year: The marginal effects of treatment group on LAZ were assessed using generalized estimating equations to model LAZ trajectories from birth until 1 year of age. A restricted cubic spline model was used, with knots set at 91, 182 and 273 days of age. Knots were selected to align with the data collection schedule. Interaction terms between treatment group and all age terms were included in the model to allow for differences in slopes between treatment arms. The marginal effects of treatment group on LAZ at birth, 3 months, 6 months, 9 months and 12 months of age, and on overall LAZ were assessed. The predicted mean LAZ was presented for the placebo group, and the mean difference (compared to the placebo group) in LAZ was presented for all other treatment groups.

(6) *Multiple imputation to impute missing length-for-age z-scores of infants at 1 year of age:* Multiple imputation by chained equations (MICE) was used to impute missing LAZ at 1 year of age. The imputation model included the main exposure (treatment assignment), outcome (LAZ at 1 year), and auxiliary variables that helped improve the performance of the missing data procedure (asset index, maternal height, LAZ at 6 months, LAZ at 9 months, and infant sex). Auxiliary variables were selected based on their relationship to missingness or LAZ at 1 year, using a liberal p-value of ≤0.1 or r^2>0.3 for inclusion in the imputation model, based on Chi-square or Fisher's tests, t-tests or Wilcoxon-rank sum tests, and correlation analyses depending on the nature of the data. Although additional LAZ measurements from different time points were also related to LAZ at 1 year, they were not included in the imputation model due to the high degree of collinearity.

The dataset was arranged in wide format, such that each participant had one row of data. Using a MICE approach, all variables included in the imputation model (treatment assignment, LAZ at 1 year, asset index, maternal height, LAZ at 6 months, LAZ at 9 months, infant sex) were imputed if data were missing. Continuous variables were imputed using linear regression and binary variables were imputed using logistic regression. Fifty imputed datasets were generated.

Missing data was imputed for: (1) All participants with missing LAZ at 1 year *except* two women who were identified as protocol violations, to generate complete datasets for 1298 mother-infant pairs; and (2) Participants with missing LAZ at 1 year, *among live births only* and *infants who did not die before 1 year of age*, and excluding two participants identified as protocol violations, generating complete datasets for 1233 mother-infant pairs.

The estimation model used linear regression to determine the association between treatment group and LAZ at 1 year, with a contrast to test the overall difference in mean LAZ at 1 year of age between treatment groups.

Method 5. Graphical representation of longitudinal anthropometric data

Population-average infant anthropometric trajectories (length-for-age z-scores, weight-for-age z-scores, weight-for-length z-scores, body mass index-for-age z-scores, head circumference-for-age z-scores) were depicted graphically based on predicted values from regression models of all available measurements (from birth to 1 year of age) as a function of age (Figure S1-S5 in the Supplementary Appendix). Restricted cubic spline models were used in which knots were set at 91, 182, and 273 days (3, 6 and 9 months). Interactions between treatment group and all age terms were included in the models to allow for differences in slopes among treatment arms. Generalized estimating equations were used to account for the within-child correlation of repeated measures. Overlaying the scatter plots of observed measurements (Figures S1-S5), coloured lines were used to distinguish predicted means for each treatment group. Missing data was not imputed for participants with an incomplete set of anthropometric measurements (birth, 3 months, 6 months, 9 months, 1 year). Measurements were not dropped from analysis on the basis of missing covariates, as age and treatment assignment were the only covariates in the models. The same models were used to generate estimated marginal effects of treatment group (Table S17).

Method 6. Determination of supplementation adherence

Data sources

To determine the actual number of supplements consumed by each participant, a manual review of supplementation logs by two independent reviewers was conducted. Any discrepancies in the count of supplements consumed between the two reviews were flagged and a third study team member reviewed the supplementation log to resolve the discrepancy.

To determine the number of scheduled doses in the prenatal and postpartum period for each participant, data from a number of study questionnaires was used, including participant enrolment and discharge dates, weekly prenatal interviews, and labour and delivery records.

Operational definitions

Adherence: Supplementation adherence was calculated from the number of doses received divided by the number of doses scheduled, multiplied by 100 to generate a percentage, stratified by the prenatal and postpartum period for each participant.

Doses administered: The number of directly observed and unobserved doses consumed.

Prenatal scheduled doses: For the majority of participants in which infant date of birth (DOB) was not missing (i.e., excluding participants who died, withdrew from the study, or were lost to follow up prior to giving birth), the number of scheduled doses in the prenatal period was calculated as the number of weeks between enrolment and infant DOB. A subset of infants were born on a day in which a regular prenatal visit was scheduled, such that the prenatal visit may not have occurred if the participant was already in labour. Participants who gave birth on the day of a scheduled prenatal visit and did not complete a prenatal visit on the same day were identified, and scheduled prenatal doses were calculated as the number of weeks between enrolment and infant DOB, minus 1 week. Among women who died or were lost to follow up prior to giving birth, the number of scheduled doses in the prenatal period was calculated as the number of weeks between enrolment and maternal death or date of last contact, respectively.

Postpartum scheduled doses: The number of scheduled doses in the postpartum period was set to 26 for all participants who were included in the complete case analysis. Among participants who exited the study prior to delivery, delivered a stillborn baby, or were lost to follow up and not successfully contacted at least once after delivery, the number of postpartum scheduled doses was set to 0. Among participants who were formally discharged between delivery and 6 months postpartum (due to infant death, loss to follow up), the number of postpartum scheduled doses was calculated as the number of weeks between infant DOB and the date the participant was discharged from the study. For participants who were successfully contacted at least once during the postpartum period but had not been officially discharged from the study before 6 months postpartum, the number of scheduled doses in the postpartum period was determined as the number of weeks between infant DOB and the date in which the participant was last successfully contacted for a weekly postpartum interview.

Total vitamin D administered: The product of the assigned dosage in the treatment group and the number of supplement doses administered, stratified by the prenatal and postpartum period for each participant.

Proportion of tablets consumed under direct observation: The number of home visits in which a supplement was consumed divided by the total supplementation doses administered.

Statistical analysis

Continuous non-normally distributed variables, including number of completed weekly monitoring visits, total supplementation doses administered, total vitamin D administered, adherence, and proportion of tablets consumed under direct observation, were reported using medians and interquartile ranges. Categorical variables were reported as frequencies and percentages and include the proportion of participants who received 100%, ≥90%, and ≥80% of scheduled doses. All variables were reported for the prenatal and postpartum period separately. To compare continuous non-normally distributed variables, the Kruskal-Wallis test was used, while Chi-square tests were used to compare categorical variables. No statistical test was used to compare total vitamin D administered between the five treatment groups.

Method 7. Development of asset index

Data source

An asset index was constructed using data collected from a baseline survey on household characteristics. Ownership (yes/no) of the following 19 items was self-reported by trial participants: private toilet, electricity, radio, TV, mobile phone, non-mobile phone, fridge, almirah/wardrobe, table, chair, electric fan, DVD/CD player, autobike, rickshaw/van, bicycle, motorcycle/motor scooter/tempo/CNG, livestock/herds/farm animals/poultry, homestead, and land.

Operational definition

Asset index: a single measure that enables relative comparisons among participants with respect to household wealth by summarizing information regarding ownership of selected items or property.

Statistical analysis

Principal component analysis was used to generate a summary measure of household wealth by reducing multiple variables into a smaller set of uncorrelated variables (or principal components)[4]. The first principal component, which accounts for the largest amount of variance between the original set of variables, is assumed to reflect relative wealth[5] and was used to assign each participant a wealth score. Lower scores reflect ownership of fewer items (i.e., less relative wealth) while higher scores are indicative of greater relative wealth. Using the distribution of asset scores in the study population, quintiles were formed and participants were categorized into 1 of 5 groups.

Figures

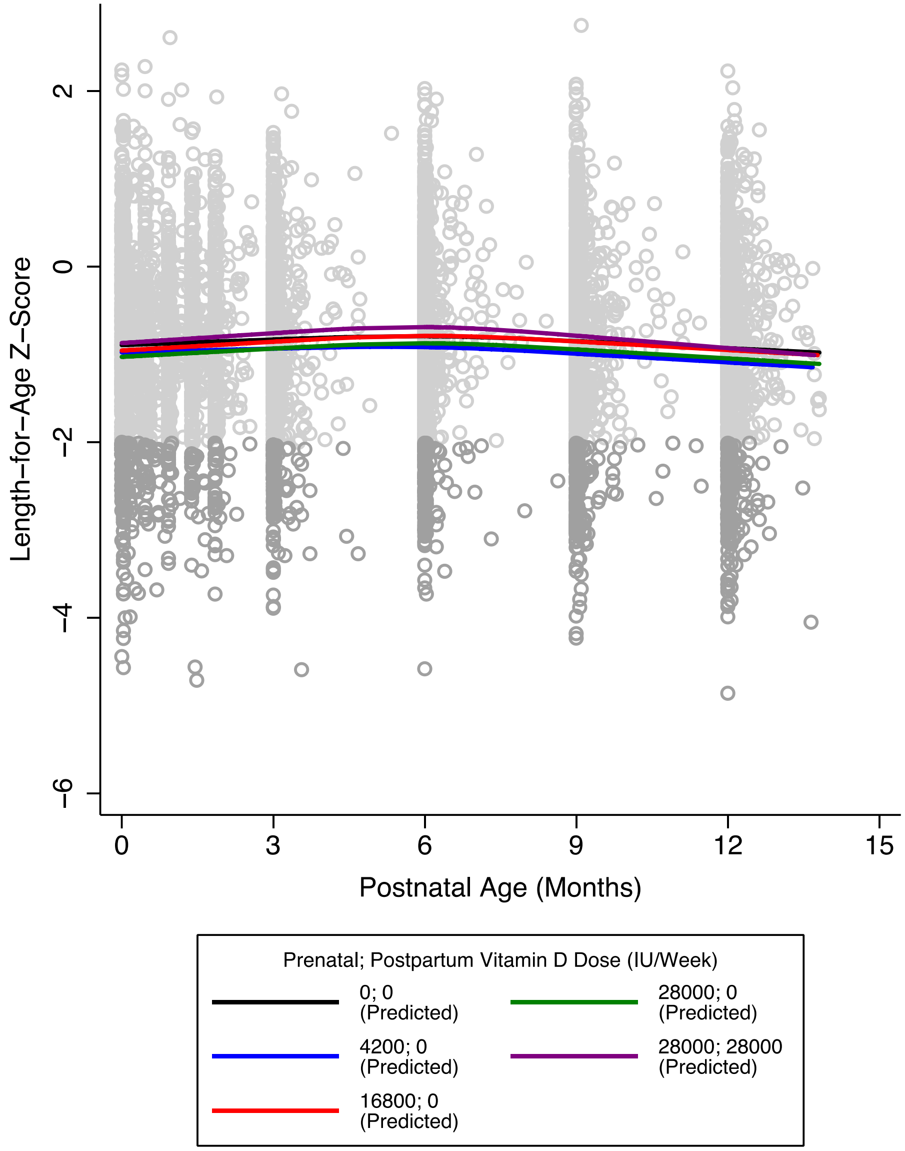

Figure S1. Length-for-age z-scores from birth to 1 year of age. Lines represent predicted means from generalized estimating equations with cubic knots at 91, 182, and 273 days and interactions between treatment groups and all age terms. Darker shading at <-2 LAZ indicates stunting.

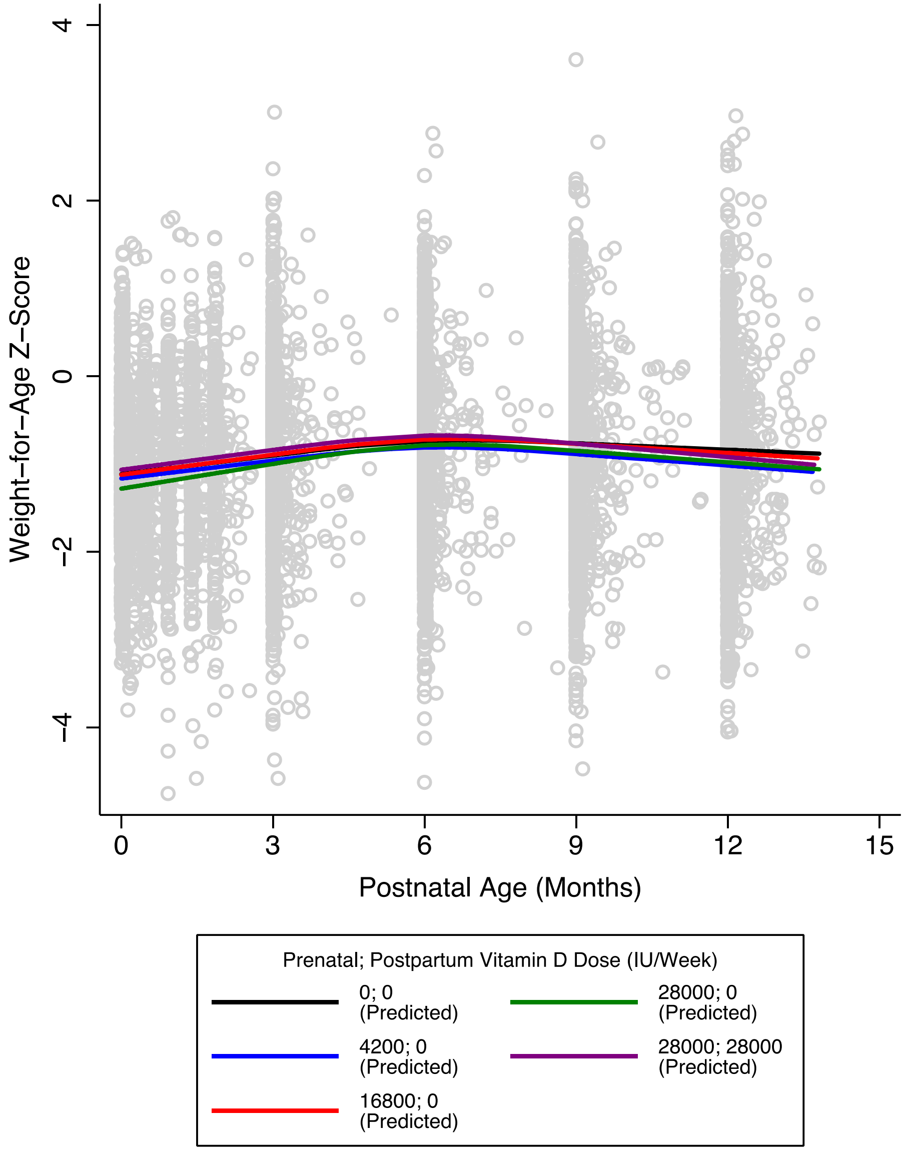

Figure S2. Weight-for-age z-scores from birth to 1 year of age. Lines represent predicted means from generalized estimating equations with cubic knots at 91, 182, and 273 days and interactions between treatment groups and all age terms.

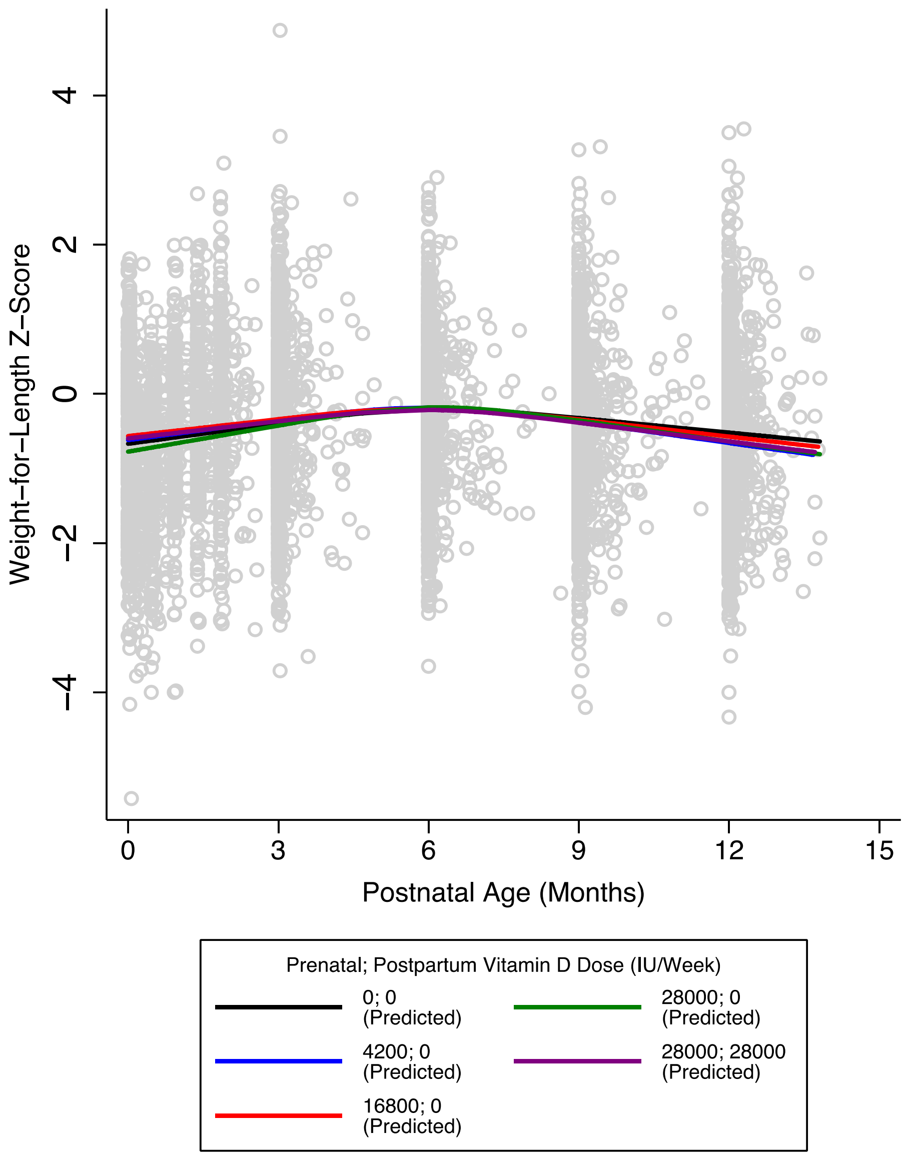

Figure S3. Weight-for-length z-scores from birth to 1 year of age. Lines represent predicted means from generalized estimating equations with cubic knots at 91, 182, and 273 days and interactions between treatment groups and all age terms.

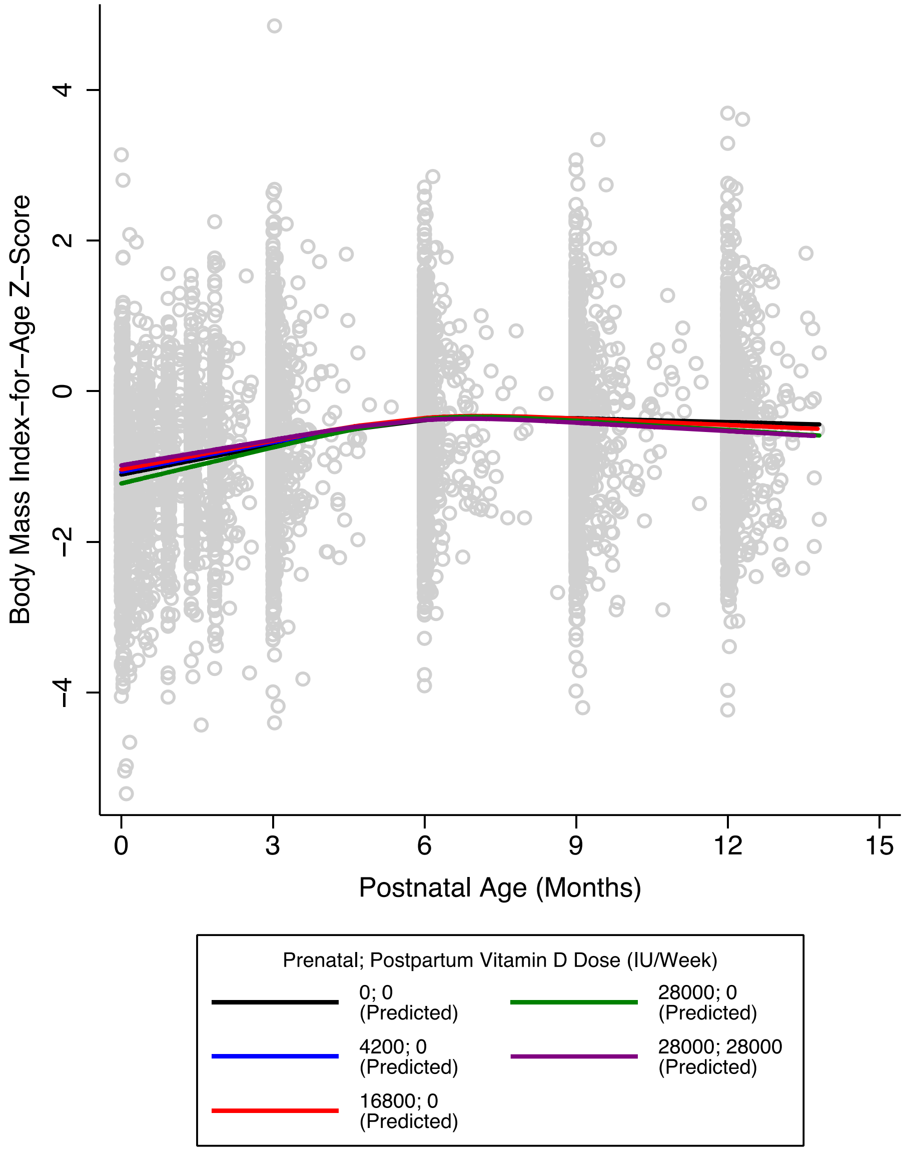

Figure S4. Body mass index-for-age z-scores from birth to 1 year of age. Lines represent predicted means from generalized estimating equations with cubic knots at 91, 182, and 273 days and interactions between treatment groups and all age terms.

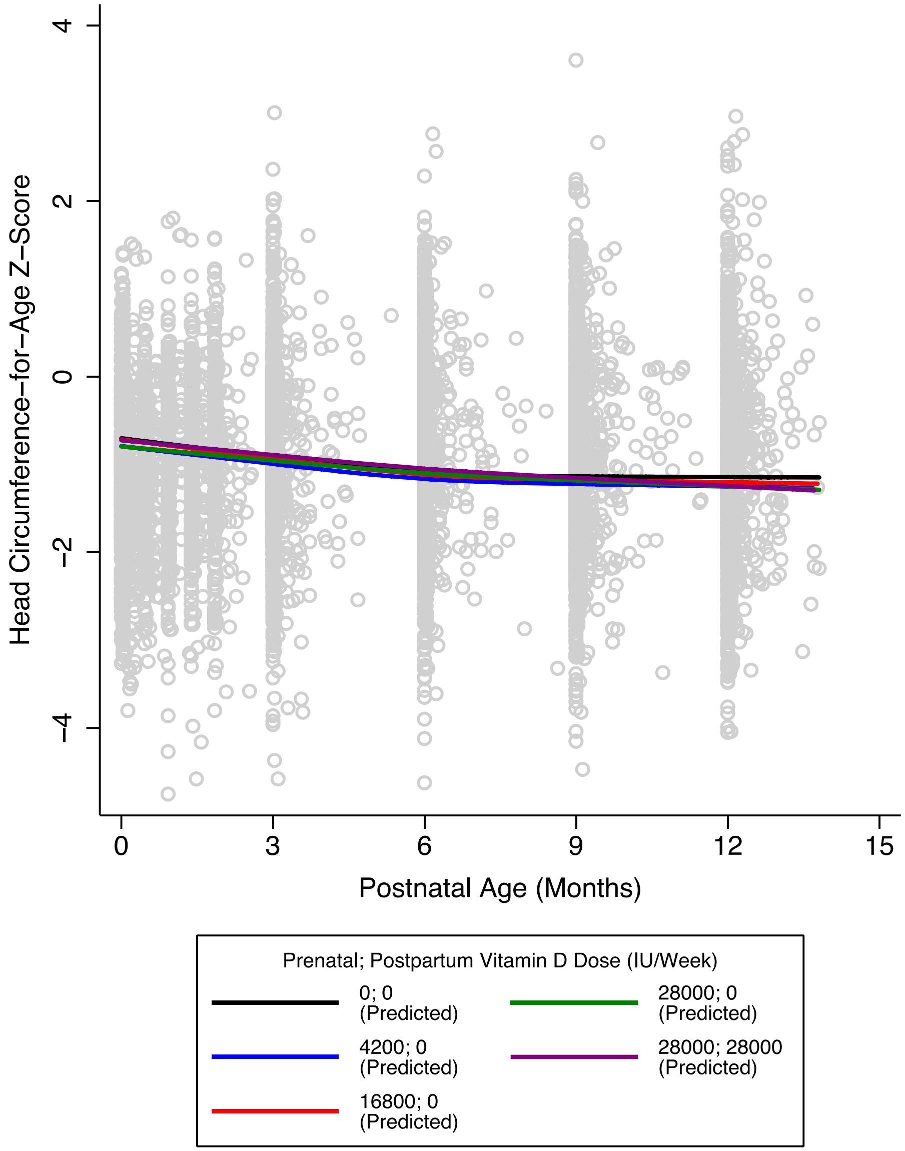

Figure S5. Head circumference-for-age z-scores from birth to 1 year of age. Lines represent predicted means from generalized estimating equations with cubic knots at 91, 182, and 273 days and interactions between treatment groups and all age terms.

Tables

Table S1. Participant inclusion and exclusion criteria

Inclusion criteria	Exclusion criteria
• Age 18 years and above;<br>• 17 to 24 completed weeks of gestation (i.e., 17 weeks +0 days to 24 weeks + 0 days, inclusive) based on recalled last menstrual period (LMP) and/or ultrasound;	• History of any medical condition or medications that may predispose to vitamin D sensitivity, altered vitamin D metabolism, and/or hypercalcemia, including active tuberculosis or current therapy for tuberculosis, sarcoidosis, history of renal/ureteral stones, parathyroid disease, renal or liver failure, or current use of anti-convulsants[a].
Rules for integrating information from recalled LMP and ultrasound:<br>• If there is a difference of >10 days between gestational age dated using the LMP and second trimester ultrasound, the estimated date of delivery will be adjusted as per the second trimester ultrasound (SOGC guidelines); otherwise (i.e., if the difference is ≤10 days), the GA date based on LMP will be used.<br>• If there is more than one ultrasound, GA estimation should be based on the earliest of the ultrasounds for which a written report is available. If the earliest ultrasound was performed in the 1st trimester, and there is a difference of >5 days between gestational age dated using the LMP and 1st trimester ultrasound, the estimated date of delivery will be adjusted as per the 1st trimester ultrasound (SOGC guidelines); otherwise (i.e., if the difference is ≤5 days), the GA date based on LMP will be used.	• High-risk pregnancy based on one or more of the following findings by point-of-care testing:<br>○ Severe anemia: hemoglobin <70 g/L assessed by Hemocue<br>○ Moderate-severe proteinuria: ≥300 mg/dl (3+ or 4+) based on urine dipstick<br>○ Hypertension: ≥1 systolic blood pressure reading ≥140 mm Hg and/or ≥1 diastolic blood pressure reading ≥90 mm Hg, in repeat measurements taken at least one minute apart<br>• High-risk pregnancy based on one or more of the following findings by maternal history and/or ultrasound:<br>○ Multiple gestation<br>○ Major congenital anomaly<br>○ Severe oligohydramnios<br>• Unwillingness to stop taking non-study vitamin D or calcium supplements or a multivitamin containing calcium and/or vitamin D.
• Intends to reside in the trial catchment area (including Hazaribag, Azimpur, Lalbag, and Kamrangirchar) for at least 18 months;	• Currently prescribed vitamin D supplements as part of a physician's treatment plan for vitamin D deficiency.
• Provides written informed consent.	• Previous enrolment in the trial during a previous pregnancy.

[a] Post-trial analysis of screening documents revealed that study physicians also occasionally excluded some women with medical conditions complicating their pregnancy that may have not directly altered vitamin D metabolism (n=84), but who were nonetheless deemed to be unlikely to enrol in the trial or complete trial procedures and follow-up.

Table S2. MDIG trial data collection procedures from enrolment to 1 year postpartum

Assessment type or procedure	Description[a]	Prenatal				Postpartum/Infant					
		Enrollment (17 to 24 weeks gestation)	30-weeks gestation	Weekly	Delivery	Weekly	2 to 8 weeks	3 months	6 months	9 months	12 months
**Maternal data and specimens**											
**Questionnaires**	Study questionnaires were completed through interview by trained study personnel. Questionnaires included items related to personal and household characteristics, behaviours, and clinical symptoms.	✓	✓	✓	✓	✓		✓	✓	✓	✓
**Blood pressure**	An automated digital blood pressure monitor was used to measure maternal systolic and diastolic blood pressure. Two consecutive readings were recorded and a third measurement was performed if either systolic or diastolic blood pressure differed by >10 mmHg.	✓	✓	✓[b]	✓						
**Urine dipstick test**	Participants urinated in a urine collection container and a test strip was immersed and quickly removed from the urine within 1 hour of collection. The test strip was compared to the coloured legend provided by the manufacturer to evaluate the amount of protein in urine.	✓			✓						
**Venous blood collection**	Blood was collected by a trained phlebotomist and centrifuged at a low speed for 15 minutes. Plasma or serum was transferred into 0.25 mL aliquots. One serum aliquot was stored at 2–8 °C for same- or	✓	✓		✓			✓	✓		

		Prenatal				Postpartum/Infant					
**Assessment type or procedure**	**Description[a]**	**Enrollment (17 to 24 weeks gestation)**	**30-weeks gestation**	**Weekly**	**Delivery**	**Weekly**	**2 to 8 weeks**	**3 months**	**6 months**	**9 months**	**12 months**
	next-day analysis of serum calcium. Additional aliquots were stored at ≤-70 °C for future analyses.										
**Urine collection for biochemistry**	Participants urinated in collection containers and samples were stored in 1.5 mL aliquots. One aliquot from the sample collected at delivery was stored at 2-8 °C for same or next-day analysis of urine calcium:creatinine ratio. Additional aliquots were stored at ≤-70 °C for future analysis.				✓						
**Height, weight**	Maternal height and weight were measured using a stadiometer and a digital floor scale. Two measurements were obtained for each parameter (height and weight) by one study worker, and third measurements were done if the difference in paired measurements was greater than 0.5 kg for weight or 2 cm for height.	✓	✓[c]		✓[c]						✓
**Infant data and specimen collection**											
**Questionnaires**	Study personnel administered questionnaires through oral interview with the infant's caregiver to collect a wide range of information related to infant behaviors and health.				✓	✓		✓	✓	✓	✓
**Height, weight, head circumference**	Infants were measured independently by two study personnel using standardized				✓		✓	✓	✓	✓	✓

Assessment type or procedure	Description[a]	Prenatal: Enrollment (17 to 24 weeks gestation)	Prenatal: 30-weeks gestation	Prenatal: Weekly	Delivery	Postpartum/Infant: Weekly	Postpartum/Infant: 2 to 8 weeks	Postpartum/Infant: 3 months	Postpartum/Infant: 6 months	Postpartum/Infant: 9 months	Postpartum/Infant: 12 months
	techniques. If paired measurements differed by more than 7 mm for length, 5 mm for head circumference, and 50 g for weight, a second set of measurements was performed. If the second set of measurements differed by more than the specified thresholds, measurements were repeated a third time.										
**Mid-upper arm circumference, upper-arm length, rump-knee length**	Infants were measured independently by two study personnel using standardized techniques. If paired measurements differed by more than 5 mm, a second set of measurements was performed. If the second set of measurements differed by more than 5 mm, measurements were repeated a third time.				✓[d]			✓	✓		✓
**Venous blood collection**	Blood was collected by a trained phlebotomist and centrifuged at a low speed for 15 minutes. Plasma or serum was then transferred in 0.25 mL aliquots into microfuge tubes. One serum aliquot was stored at 2–8 °C for same- or next-day analysis of serum calcium. The aliquot at 6 months of age was also analyzed for serum phosphate and alkaline phosphatase. Additional aliquots were stored at ≤-70 °C for future analyses.				✓ (cord blood)			✓	✓		✓

		Prenatal				Postpartum/Infant					
**Assessment type or procedure**	**Description[a]**	**Enrollment (17 to 24 weeks gestation)**	**30-weeks gestation**	**Weekly**	**Delivery**	**Weekly**	**2 to 8 weeks**	**3 months**	**6 months**	**9 months**	**12 months**
**Urine collection**	Infant urine was collected using urine collection bags that adhered to the perineum. One aliquot was stored at 2-8 °C for same or next-day analysis of urine calcium:creatinine ratio. Other 1.5 mL aliquots were stored at ≤-70 °C for future analysis.								✓		

[a] Methods are described in detail elsewhere[6].

[b] Blood pressure measured at 24 and 30 weeks gestation, and weekly from 36 weeks gestation to delivery

[c] Only weight was measured.

[d] Only upper-arm length and rump-knee-length were measured.

Table S3. Quality control of anthropometric measures at each scheduled measurement time point

	Birth<br>N = 1090	2-8 Weeks<br>N = 1101	3 Months<br>N = 1133	6 Months<br>N = 1133	9 Months<br>N = 1128	12 Months<br>N = 1164
**Crown-to-heel length**						
Technical error of measurement of first set of paired measurements (cm)[a]	0.22	0.32	0.25	0.26	0.29	0.21
Number of instances in which…						
Difference between first set of paired measurements was greater than 0.7 cm	9	17	18	8	7	4
Difference between final set of paired measurements was greater than 0.7 cm	1	4	1	0	2	1
Only one measurement was taken	2	0	0	0	0	0
Measurement was flagged based on WHO criteria (length-for-age z-score >6 or <-6)	0	2	0	0	0	0
Measurement was flagged as an outlier based on residual method[b]	54	18	14	10	4	9
Measurement was inconsistent with a measurement at a previous or later time point	2	9	6	0	0	0
**Weight**						
Technical error of measurement of first set of paired measurements (g)[a]	16.2	17.7	27.1	17.5	14.5	9.9
Number of instances in which…						
Difference between first set of paired measurements was greater than 50 g	2	13	7	12	9	12
Difference between final set of paired measurements was greater than 50 g	2	3	1	1	2	1
Only one measurement was taken	2	2	2	0	0	0
Measurement was flagged based on WHO criteria (weight-for-age z-score >5 or <-6)	0	0	0	0	0	1
Measurement was flagged as an outlier based on residual method[b]	90	9	7	9	5	7
Measurement was inconsistent with a measurement at a previous or later time point	0	1	0	0	1	3
**Head circumference**						
Technical error of measurement of first set of paired measurements (cm)[a]	0.26	0.23	0.21	0.21	0.19	0.21
Number of instances in which…						
Difference between first set of paired measurements was greater than 0.5 cm	17	22	14	12	7	6
Difference between final set of paired measurements was greater than 0.5 cm	1	4	2	1	1	1
Only one measurement was taken	2	1	3	0	0	0
Measurement was flagged based on WHO criteria (head circumference-for-age z-score >5 or <-5)	0	1	2	3	4	2
Measurement was flagged as an outlier based on residual method[b]	66	22	11	10	12	3
Measurement was inconsistent with a measurement at a previous or later time point	7	6	2	4	17	10
**Upper-arm length**						
Technical error of measurement of first set of paired measurements (cm)[a]	0.21	-	0.22	0.21	-	0.2
Number of instances in which…						
Difference between first set of paired measurements was greater than 0.5 cm	17	-	10	7	-	3
Difference between final set of paired measurements was greater than 0.5 cm	0	-	0	2	-	0
Only one measurement was taken	0	-	1	0	-	0

	Birth	2-8 Weeks	3 Months	6 Months	9 Months	12 Months
	N = 1090	N = 1101	N = 1133	N = 1133	N = 1128	N = 1164
Measurement was flagged as an outlier based on residual method[c]	53	-	65	76	-	68
Measurement was inconsistent with a measurement at a previous or later time point	1	-	2	5	-	0
**Mid-upper arm circumference**						
Technical error of measurement of first set of paired measurements (cm)[a]	-	-	0.18	0.19	-	0.19
Number of instances in which…						
Difference between first set of paired measurements was greater than 0.5 cm	-	-	4	5	-	0
Difference between final set of paired measurements was greater than 0.5 cm	-	-	1	0	-	0
Only one measurement was taken	-	-	1	0	-	0
Measurement was flagged based on WHO criteria (mid-upper arm circumference-for-age z-score >5 or <-5)	-	-	0	0	-	0
Measurement was flagged as an outlier based on residual method[b]	-	-	54	45	-	50
**Rump-knee length**						
Technical error of measurement of first set of paired measurements (cm)[a]	0.17	-	0.18	0.18	-	0.18
Number of instances in which…						
Difference between first set of paired measurements was greater than 0.5 cm	5	-	8	5	-	6
Difference between final set of paired measurements was greater than 0.5 cm	2	-	1	1	-	0
Only one measurement was taken	0	-	0	0	-	0
Measurement was flagged as an outlier based on residual method[c]	84	-	54	43	-	69
Measurement was inconsistent with a measurement at a previous or later time point	1	-	5	6	-	3

[a] Technical error of measurement = $\sqrt{\frac{\sum d^2}{2n}}$, where *d* is the difference between paired measurements, and *n* is the number of observations at a given measurement time point.

[b] Linear regression was used to fit a straight line through an individual's age-standardized z-score (Z) as a function of age (t): $Z_{ij} = \beta_{0i} + \beta_i t_{ij} + \varepsilon_{ij}$; where "*i*" is the ith individual and "*j*" is the jth time point. Based on the model predicted for each individual, jackknife residuals were calculated, and measurements with an absolute jackknife residual ≥5 were flagged as outliers for length, weight, and head circumference, and measurements with an absolute jackknife residual ≥7 were flagged as outliers for mid-upper arm circumference.

[c] An individual's raw anthropometric measurement (Y) was regressed on the square root of age ($t^{1/2}$) to model a curvilinear relationship: $Y_{ij} = \beta_{0i} + \beta_i t_{ij}^{½} + \varepsilon_{ij}$; where "*i*" is the ith individual and "*j*" is the jth time point. Based on the model predicted for each individual, jackknife residuals were calculated and measurements with an absolute jackknife residual ≥7 were flagged as outliers.

Table S4. List of secondary outcomes

Secondary outcome	Pre-specified in protocol or post-hoc
**Anthropometric outcomes**[a]	
Length-for-age z-score<-2 (stunting) at 1 year	Pre-specified in protocol
Weight-for-age z-score at 1 year	Pre-specified in protocol
Weight-for-length z-score at 1 year	Pre-specified in protocol
Body mass index-for-age z-score at 1 year	Post-hoc
Head circumference for age z-score at 1 year	Pre-specified in protocol
Mid-upper arm circumference-for-age z-score at 1 year	Post-hoc
Weight-for-length z-score<-2 (wasting) at 1 year	Pre-specified in protocol
Head circumference-for-age z-score <-2 at 1 year	Pre-specified in protocol
Weight-for-age z-score <-2 at 1 year	Pre-specified in protocol
Weight-for-age z-score at birth	Pre-specified in protocol
Length-for-age z-score at birth	Pre-specified in protocol
Head circumference for age z-score at birth	Pre-specified in protocol
Length-for-age z-score <-2 at birth	Pre-specified in protocol
Weight-for-age z-score <-2 at birth	Pre-specified in protocol
Head circumference-for-age z-score <-2 at birth	Pre-specified in protocol
Small-for-gestational age	Pre-specified in protocol
Low birth weight	Pre-specified in protocol
Term low birth weight	Post-hoc
Placental weight	Pre-specified in protocol
Length-for-age z-score, weight-for-age z-score, weight-for-length z-score, body mass index-for-age z-score, and head circumference for age z-score trajectories from birth to 1 year	Pre-specified in protocol for length; post-hoc for other standardized anthropometric parameters[b]
Raw anthropometric measurements at 1 year (length, weight, head circumference, upper arm length, mid-upper arm circumference, rump-to-knee length)	Pre-specified in protocol[c]
Raw length, head circumference, and weight at birth	Pre-specified in protocol
**Biochemical outcomes**	
Serum 25(OH)D concentrations<br>• Maternal 25(OH)D at baseline, delivery, 3 and 6 months postpartum<br>• Venous cord 25(OH)D<br>• Infant 25(OH)D at 3, 6, and 12 months of age<br>• Reported as means and n (%) above/below cut-offs.	Pre-specified in protocol (except for maternal 25(OH)D at 3 months and infant 25(OH)D at 12 months)[d]<br>25(OH)D thresholds for categorization were defined post-hoc.
Serum C3-epi-25(OH)D and total 25(OH)D concentrations<br>• Same time points as identified above for 25(OH)D)	Post-hoc

Serum calcium concentration<br>• Reported as means and n (%) above cut-offs<br>• Maternal in pregnancy and up to 6 months postpartum<br>• Infant, up to 6 months of age	Pre-specified in protocol[e]
Urinary calcium:creatinine ratio<br>• Reported as medians and n (%) with hypercalciuria<br>• Maternal, at delivery<br>• Infant, at 6 months of age	Post-hoc[f]
Intact parathyroid hormone plasma concentration<br>• Maternal, at delivery and 6 months postpartum	Post-hoc[f]
**Pregnancy/delivery and other clinical outcomes**	
Live birth or stillbirth	Pre-specified in protocol
Gestational age at birth and preterm birth	Pre-specified in protocol[g]
Mode of delivery (C-section or vaginal birth)	Post-hoc
Location of delivery	Post-hoc
Congenital anomalies	Post-hoc
Maternal and infant clinical encounters	Post-hoc
Infant and maternal primary diagnoses at clinical encounters and primary discharge diagnoses for hospitalizations	Pre-specified in protocol[h]
Infant and maternal reported symptoms	Post-hoc
Maternal urolithiasis/nephrolithiasis	Pre-specified in protocol
Maternal and infant hospitalizations	Pre-specified in protocol
Gestational hypertension	Post-hoc
Participant (woman) death at any time and from any cause	Pre-specified in protocol
Maternal death (related to the pregnancy)	Pre-specified in protocol
Maternal referral to a specialist or hospital up to one-month postpartum	Pre-specified in protocol[i]
Rickets (x-ray confirmed)	Post-hoc
Infant neurological disabilities	Post-hoc
Infant death<br>• Any time point, and stratified by the neonatal period, 29 days-< 6 months, and 6-12 months	Pre-specified in protocol

[a] The protocol referred to anthropometric outcomes up to 2 years of age; however, reported measurements are those obtained up to and including the one-year visit (at which the primary outcome was ascertained).
[b] The protocol stated that “linear growth velocity” would be reported but the analytical approach was not pre-specified; therefore, longitudinal trajectories were modeled using regression cubic splines to reflect between-group differences in interval growth. Data beyond 1 year are not reported.
[c] The protocol stated that raw weight, length, limb length, mid-upper arm circumference, and head circumference would be reported at all discrete measurement time points; however, given the null findings at one year, all raw measurements are only summarized at the 1 year time point. Raw weight, length and head circumference are also reported at birth.

[d] The protocol stated that the 25(OH)D "change in concentration during pregnancy" would also be reported. This is depicted visually in Figure 2, and mean differences can be derived from the Table S23.

[e] Only maternal serum calcium was pre-specified as a safety endpoint; however, summaries of infant serum calcium data are also reported.

[f] These biochemical measurements were described in the Protocol, but were not pre-specified as secondary outcomes.

[g] The protocol specified mean gestational age at delivery would be reported. Median (min, max) gestational age at delivery was instead reported because this variable is not normally distributed. The protocol referred to the sub-category of "early preterm" as < 34 weeks; however, this category was defined as <32 weeks in the main analyses, in which a new category of moderate to late preterm (≥32 weeks to <37 weeks) was also generated. Additional analyses were performed using <34 weeks to define early preterm births.

[h] Only infant morbidity was pre-specified in the protocol. However, summaries of all maternal and infant clinical encounters with physicians and hospitalizations are reported. Also, the protocol specified the "occurrence of episodes of skin infection, sepsis, diarrhea or acute respiratory infection (ARI)" in infants. These outcomes are reported in the primary diagnoses and discharge diagnoses tables; however, the term "skin rash" is used instead of "skin infection" as it was unfeasible to determine whether skin conditions were caused by infections based on the data collected.

[i] The protocol referred to "maternal referral for obstetric care" in the "prenatal period, and up to 1 month postpartum." The reported outcome includes all referrals of women in this time frame, since criteria for defining an "obstetric complication" were unspecified.

Table S5. Additional maternal characteristics at enrolment, by supplementation group

	Prenatal; Postpartum Vitamin D Dose (IU/Week)				
	**0; 0**	**4200; 0**	**16800; 0**	**28000; 0**	**28000; 28000**
**Enrolled participants, N**	**259[a]**	**260**	**259[a]**	**260**	**260**
Level of education, n (%)					
No schooling	10 (3.9)	12 (4.6)	12 (4.6)	14 (5.4)	10 (3.8)
Primary incomplete	57 (22.0)	50 (19.2)	57 (22.0)	54 (20.8)	59 (22.7)
Primary complete	35 (13.5)	38 (14.6)	25 (9.7)	36 (13.8)	45 (17.3)
Secondary incomplete	105 (40.5)	90 (34.6)	114 (44.0)	98 (37.7)	91 (35.0)
Secondary complete or higher	52 (20.1)	70 (26.9)	51 (19.7)	58 (22.3)	55 (21.2)
Asset index quintile, n (%)[b]					
1 (lowest)	55 (21.4)	59 (22.9)	38 (15.0)	61 (23.8)	48 (18.8)
2	49 (19.1)	54 (20.9)	57 (22.5)	45 (17.6)	46 (18.0)
3	58 (22.6)	41 (15.9)	54 (21.3)	51 (19.9)	52 (20.3)
4	49 (19.1)	44 (17.1)	56 (22.1)	53 (20.7)	55 (21.5)
5 (highest)	46 (17.9)	60 (23.3)	48 (19.0)	46 (18.0)	55 (21.5)
Month of enrolment, n (%)					
March-May	94 (36.3)	89 (34.2)	102 (39.4)	93 (35.8)	87 (33.5)
June-August	86 (33.2)	79 (30.4)	73 (28.2)	91 (35.0)	83 (31.9)
September-November	40 (15.4)	49 (18.8)	46 (17.8)	41 (15.8)	49 (18.8)
December-February	39 (15.1)	43 (16.5)	38 (14.7)	35 (13.5)	41 (15.8)
Hemoglobin (g/L), mean ± SD	105.6 ± 11.3	106.3 ± 12.0	106.0 ± 11.1	106.1 ± 10.9	106.8 ± 11.8

[a] One participant excluded due to a protocol violation.

[b] $N_{0;\,0}$ = 257, $N_{4200;\,0}$ = 258, $N_{16800;\,0}$ = 253, $N_{28000;\,0}$ = 256, $N_{28000;\,28000}$ = 256. Higher scores indicate greater household asset ownership relative to other participants. See Method 7 in the Supplementary Appendix for a description of the construction and interpretation of the asset index.

Table S6. Maternal characteristics at enrolment for participants included in the complete case analysis, by supplementation group

	Prenatal; Postpartum Vitamin D Dose (IU/Week)				
**Characteristic**	**0; 0**	**4200; 0**	**16800; 0**	**28000; 0**	**28000; 28000**
**Enrolled participants, N**	**229**	**237**	**237**	**230**	**231**
Age (years), median (min, min)	23 (18, 38)	23 (18, 40)	22 (18, 35)	22 (18, 38)	23 (18, 38)
Gestational age (weeks), median (min, max)	20.6 (17, 24)	20.3 (17, 24)	20.3 (17, 24)	20.4 (17, 24)	20.1 (17, 24)
Marital status, n (%)[a]					
Married	227 (99.1)	237 (100.0)	237 (100.0)	228 (100.0)	230 (100.0)
Not Married	2 (0.9)	0 (0.0)	0 (0.0)	0 (0.0)	0 (0.0)
Level of education, n (%)					
No schooling	9 (3.9)	11 (4.6)	10 (4.2)	11 (4.8)	7 (3.0)
Primary incomplete	52 (22.7)	45 (19.0)	50 (21.1)	50 (21.7)	50 (21.6)
Primary complete	33 (14.4)	34 (14.3)	23 (9.7)	34 (14.8)	39 (16.9)
Secondary incomplete	88 (38.4)	83 (35.0)	105 (44.3)	86 (37.4)	84 (36.4)
Secondary complete or higher	47 (20.5)	64 (27.0)	49 (20.7)	49 (21.3)	51 (22.1)
Primary occupation, n (%)[a]					
Homemaker	214 (93.4)	221 (93.2)	222 (93.7)	213 (93.4)	216 (93.9)
Other	15 (6.6)	16 (6.8)	15 (6.3)	15 (6.6)	14 (6.1)
Asset index quintiles, n (%)[b]					
1 (lowest)	49 (21.4)	53 (22.5)	32 (13.6)	51 (22.5)	42 (18.3)
2	43 (18.8)	46 (19.5)	56 (23.7)	38 (16.7)	39 (17.0)
3	56 (24.5)	39 (16.5)	49 (20.8)	46 (20.3)	48 (20.9)
4	41 (17.9)	40 (16.9)	53 (22.5)	51 (22.5)	50 (21.7)
5 (highest)	40 (17.5)	58 (24.6)	46 (19.5)	41 (18.1)	51 (22.2)
Gravidity[c], median (min, min)	2 (1, 9)	2 (1, 6)	2 (1, 6)	2 (1, 7)	2 (1, 6)
Parity, median (min, min)	2 (0, 6)	2 (0, 5)	2 (0, 5)	2 (0, 5)	2 (0, 4)
Height (cm), mean ± SD	151.1 ± 5.5	151.0 ± 5.0	150.7 ± 5.4	150.0 ± 5.4	151.7 ± 5.5
Weight (kg), mean ± SD	54.6 ± 10.2	53.4 ± 10.0	53.9 ± 10.0	53.2 ± 9.0	55.0 ± 10.3
Month of enrolment, n (%)					
March-May	84 (36.7)	79 (33.3)	95 (40.1)	85 (37.0)	77 (33.3)
June-August	75 (32.8)	73 (30.8)	68 (28.7)	79 (34.3)	74 (32.0)
September-November	37 (16.2)	47 (19.8)	39 (16.5)	34 (14.8)	46 (19.9)
December-February	33 (14.4)	38 (16.0)	35 (14.8)	32 (13.9)	34 (14.7)
Hemoglobin (g/L), mean ± SD	105.5 ± 11.0	106.4 ± 11.9	106.6 ± 10.8	105.6 ± 10.8	106.8 ± 11.8
Serum 25(OH)D concentration (nmol/L), mean ± SD[d]	27.6 ± 14.0	27.7 ± 14.3	28.5 ± 14.2	27.2 ± 15.0	26.4 ± 13.3

[a] $N_{0;\,0}$ = 229, $N_{4200;\,0}$ = 237, $N_{16800;\,0}$ = 237, $N_{28000;\,0}$ = 228, $N_{28000;\,28000}$ = 230

[b] $N_{0;\,0}$ = 229, $N_{4200;\,0}$ = 236, $N_{16800;\,0}$ = 236, $N_{28000;\,0}$ = 227, $N_{28000;\,28000}$ = 230

[c] Number of pregnancies, including the current pregnancy.

[d] $N_{0;\,0}$ = 226, $N_{4200;\,0}$ = 235, $N_{16800;\,0}$ = 236, $N_{28000;\,0}$ = 228, $N_{28000;\,28000}$ = 227

Table S7. Infant feeding practices among participants included in the complete-case analysis during the first 6 months of age, by supplementation group

Infant Feeding Variable	0; 0<br>N = 229	4200; 0<br>N = 237	16800; 0<br>N = 237	28000; 0<br>N = 230	28000; 28000<br>N = 231	p[a]
	Prenatal; Postpartum Vitamin D Dose (IU/Week)					
Initiated breastfeeding within 1h of birth, n (%)[b]	38 (18.2)	30 (14.0)	37 (16.7)	34 (16.6)	27 (13.0)	0.569
Duration of exclusive breastfeeding (weeks)[c], median (min, max)	12.5 (0, 26)	14 (0, 26)	12 (0, 26)	13 (0, 26)	14 (0, 26)	0.321
Feeding patterns to 3 months of age[d], n (%)						0.492
Exclusive breastfeeding	105 (45.9)	121 (51.1)	108 (45.6)	111 (48.3)	119 (51.5)	
Predominant breastfeeding	18 (7.9)	27 (11.4)	20 (8.4)	22 (9.6)	25 (10.8)	
Partial breastfeeding	79 (34.5)	70 (29.5)	85 (35.9)	80 (34.8)	65 (28.1)	
No breastfeeding	15 (6.6)	6 (2.5)	10 (4.2)	5 (2.2)	7 (3.0)	
Unable to classify	12 (5.2)	13 (5.5)	14 (5.9)	12 (5.2)	15 (6.5)	
Feeding patterns to 6 months of age[e], n (%)						0.515
Exclusive breastfeeding	30 (13.1)	36 (15.2)	32 (13.5)	31 (13.5)	28 (12.1)	
Predominant breastfeeding	21 (9.2)	13 (5.5)	15 (6.3)	11 (4.8)	19 (8.2)	
Partial breastfeeding	144 (62.9)	158 (66.7)	159 (67.1)	155 (67.4)	155 (67.1)	
No breastfeeding	26 (11.4)	20 (8.4)	22 (9.3)	17 (7.4)	14 (6.1)	
Unable to classify	8 (3.5)	10 (4.2)	9 (3.8)	16 (7.0)	15 (6.5)	
Infant formula ever given[f], n (%)[g]	139 (62.3)	144 (62.9)	153 (66.5)	138 (62.7)	139 (62.3)	0.869

[a] P-value from Kruskal-Wallis tests for continuous variables and Chi-square or Fischer's tests for categorical variables.

[b] $N_{0;\,0}$ = 209, $N_{4200;\,0}$ = 215, $N_{16800;\,0}$ = 221, $N_{28000;\,0}$ = 205, $N_{28000;\,28000}$ = 208

[c] The number of continuous weeks from birth in which an infant was classified as exclusively breastfed (breastmilk only), excluding the first week of life.

[d] 3 months of age refers to feeding patterns from birth to 13 weeks of age. Each child-week was classified as exclusively breastfed (breastmilk only); predominantly breastfed (breast milk with water, sugar water, honey, or other non-milk, non-formula liquid); partially breastfed (breast milk with animal, powdered or condensed milk, and solid or semi-solid foods); and not breastfed. Classification of each infant was defined according to the lowest breastfeeding category achieved in any observed week from birth to 3 months of age. Infants with missing data for weeks 12 and 13 were deemed not able to be classified.

[e] 6 months of age refers to feeding patterns from birth to 27 weeks of age. Feeding patterns were classified using the same method used in the birth to 3 month interval, except infants with missing data for weeks 25 and 26 were deemed not able to be classified.

[f] A participant must have had at least 13 weekly visits completed in order to be classified as never having been given infant formula during the first six months of age. Minimum data requirements did not apply if the participant was ever given infant formula.

[g] $N_{0;\,0}$ = 223, $N_{4200;\,0}$ = 229, $N_{16800;\,0}$ = 230, $N_{28000;\,0}$= 220, $N_{28000;\,28000}$ = 223

Table S8. Infant vitamin D consumption from birth to 1 year of age

	Prenatal; Postpartum Vitamin D Dose (IU/Week)					
	0; 0<br>N= 247	4200; 0<br>N=254	16800; 0<br>N=252	28000; 0<br>N=252	28000; 28000<br>N=249	p[a]
Infant age (days) when first vitamin/supplement containing or possibly containing vitamin D was consumed[b], median (IQR)	85 (39, 155)	105 (14, 141)	60 (25, 150)	70 (28, 119)	63 (35, 133)	0.829
Ever consumed a vitamin/supplement containing or possibly containing vitamin D from birth to 1 year[b], n (%)	40 (16.2)	25 (9.8)	40 (15.9)	31 (12.3)	34 (13.6)	0.203
Ever consumed a vitamin D-only supplement from birth to 1 year, n (%)	2 (0.8)	2 (0.8)	1 (0.4)	0 (0.0)	0 (0.0)	0.429
Ever consumed a multivitamin/supplement known to contain vitamin D from birth to 1 year, n (%)	18 (7.3)	14 (5.5)	24 (9.5)	14 (5.6)	13 (5.2)	0.251
Ever consumed a multivitamin or supplement of unknown formulation from birth to 1 year, n (%)	26 (10.5)	16 (6.3)	25 (9.9)	24 (9.5)	26 (10.4)	0.455
Number of weeks a vitamin/supplement containing or possibly containing vitamin D was consumed from birth to 6 months of age[b,c], n (%)						
0	211 (85.4)	233 (91.8)	215 (85.3)	222 (88.1)	217 (87.1)	0.434
1	25 (10.1)	10 (3.9)	19 (7.5)	17 (6.7)	20 (8.0)	
2-3	8 (3.2)	7 (2.8)	14 (5.6)	10 (4.0)	10 (4.0)	
4-5	2 (0.8)	3 (1.2)	4 (1.6)	1 (0.4)	2 (0.8)	
6+	1 (0.4)	1 (0.4)	0 (0)	2 (0.8)	0 (0)	
Number of weeks a supplement containing or possibly containing vitamin D was consumed among infants with at least 1 week of reported consumption from birth to 6 months of age[b,c], median (IQR)	1 (1, 2)	2 (1, 3)	1 (1, 2)	1 (1, 3)	1 (1, 2)	0.465
Treatment for rickets with vitamin D[d]						
Ever treated for rickets from birth to 1 year, n (%)	3 (1.8)	1 (0.6)	1 (0.6)	2 (1.3)	1 (0.7)	0.850
Assessed by physician for possible rickets but prescribed only prophylactic doses of vitamin D from birth to 1 year[e], n (%)	1 (0.6)	0 (0.0)	1 (0.6)	0 (0.0)	1 (0.7)	0.752

[a] P-value from Kruskal-Wallis test for continuous variables and Chi-square or Fisher's test for categorical variables.

[b] Includes vitamin D supplements containing only vitamin D, multivitamins/supplements known to contain vitamin D, and multivitamins or other supplements of unknown formulation, from birth to 1 year of age.

[c] From birth to 6 months of age, after which weekly visits stopped.

[d] Denominator is number of infants screened for rickets within 1 year of age. $N_{0; 0}$ = 165, $N_{4200; 0}$ = 161, $N_{16800; 0}$ = 154, $N_{28000; 0}$ =155, $N_{28000; 28000}$ = 152

[e] Prophylactic doses were 400 or 800 IU vitamin D/day.

Table S9. Comparison of maternal characteristics at enrolment between participants included in the complete case analysis and participants excluded from the complete case analysis

	Participants excluded from the complete case analysis	Participants included in the complete case analysis	p[a]
**Enrolled participants, N**	**136**	**1164**	
Age (years), median (min, min)	22 (18.0, 36.0)	23 (18.0, 40.0)	0.120
Gestational age (weeks), median (min, max)	20.1 (17.1, 24.0)	20.3 (17.0, 24.0)	0.732
Marital status, n (%)[b]			0.999
Married	124 (100.0)	1158 (99.8)	
Not Married	0 (0.0)	2 (0.2)	
Level of education, n (%)			0.411
No schooling	10 (7.4)	48 (4.1)	
Primary incomplete	30 (22.1)	247 (21.2)	
Primary complete	16 (11.8)	163 (14.0)	
Secondary incomplete	54 (39.7)	446 (38.3)	
Secondary complete or higher	26 (19.1)	260 (22.3)	
Primary occupation, n (%)[b]			0.480
Homemaker	118 (95.2)	1086 (93.5)	
Other	6 (4.8)	75 (6.5)	
Asset index quintiles, n (%)[c]			0.077
1 (lowest)	35 (28.2)	227 (19.6)	
2	29 (23.4)	222 (19.2)	
3	19 (15.3)	238 (20.6)	
4	22 (17.7)	235 (20.3)	
5 (highest)	19 (15.3)	236 (20.4)	
Gravidity[d], median (min, min)	2 (1.0, 6.0)	2 (1.0, 9.0)	0.966
Parity, median (min, min)	1 (0.0, 5.0)	2 (0.0, 6.0)	0.216
Height (cm), mean ± SD	151.2 ± 5.5	150.9 ± 5.4	0.592
Weight (kg), mean ± SD	53.9 ± 10.9	54.0 ± 9.9	0.876
Month of enrolment, n (%)			0.809
March-May	46 (33.8)	420 (36.1)	
June-August	44 (32.4)	369 (31.7)	
September-November	22 (16.2)	203 (17.4)	
December-February	24 (17.6)	172 (14.8)	
Hemoglobin (g/L), mean ± SD	105.5 ± 12.5	106.2 ± 11.3	0.498
Serum 25(OH)D concentration (nmol/L), mean ± SD[e]	27.3 ± 12.8	27.5 ± 14.2	0.915

[a] P-value from ANOVA or Kruskal-Wallis tests for continuous variables and Chi-square or Fisher's tests for categorical variables.
[b] N = 124 and N = 1161
[c] N = 124 and N= 1158 for those excluded from and included in the complete case analysis.
[d] Number of pregnancies, including the current pregnancy.
[e] N = 133 and N = 1152 for those excluded from and included in the complete case analysis.

Table S10. Supplementation duration and adherence among participants included in the complete case analysis, by supplementation group

	Prenatal; Postpartum Vitamin D Dose (IU/Week)					p[a]
	0; 0<br>N = 229	4200; 0<br>N = 237	16800; 0<br>N = 237	28000; 0<br>N = 230	28000; 28000<br>N = 231	
**Prenatal adherence**						
Completed weekly monitoring visits, median (IQR)	17 (16, 19)	17 (16, 19)	18 (16, 19)	18 (16, 19)	18 (16, 20)	0.414
Total supplement doses administered, median (IQR)	18 (17, 20)	19 (17, 21)	19 (17, 21)	19 (17, 21)	19 (17, 21)	0.193
Total vitamin D administered (× 1,000 IU), median (IQR)	0 (0,0)	80 (71, 88)	319 (286, 353)	532 (476, 588)	532 (476, 588)	-
Adherence (%)[b], median (IQR)	100 (100, 100)	100 (100, 100)	100 (100, 100)	100 (100, 100)	100 (100, 100)	0.255
Participants who received						
100% of scheduled doses, n (%)	201 (87.8)	209 (88.2)	206 (86.9)	194 (84.3)	210 (90.9)	0.313
≥90% of scheduled doses, n (%)	221 (96.5)	227 (95.8)	223 (94.1)	209 (90.9)	225 (97.4)	0.013[c]
≥80% of scheduled doses, n (%)	223 (97.4)	230 (97.0)	228 (96.2)	214 (93.0)	227 (98.3)	0.030[c]
Proportion of tablets consumed under direct observation, median (IQR)	91 (88, 95)	90 (86, 95)	93 (87, 95)	91 (86, 95)	91 (88, 95)	0.671
**Postpartum adherence**						
Completed weekly monitoring visits, median (IQR)	24 (23, 25)	24 (22, 25)	24 (22, 25)	24 (23, 25)	24 (23, 25)	0.712
Total supplement doses administered, median (IQR)	26 (26, 26)	26 (25, 26)	26 (25, 26)	26 (25, 26)	26 (25, 26)	0.179
Total vitamin D administered (× 1,000 IU), median (IQR)	0 (0,0)	0 (0, 0)	0 (0, 0)	0 (0, 0)	728 (700, 728)	-
Adherence (%)[b], median (IQR)	100 (100, 100)	100 (96, 100)	100 (96, 100)	100 (96, 100)	100 (96, 100)	0.179
Participants who received						
100% of scheduled doses, n (%)	176 (76.9)	168 (70.9)	177 (74.7)	156 (67.8)	172 (74.5)	0.202
≥90% of scheduled doses, n (%)	202 (88.2)	192 (81.0)	202 (85.2)	187 (81.3)	202 (87.4)	0.089
≥80% of scheduled doses, n (%)	207 (90.4)	204 (86.1)	211 (89.0)	200 (87.0)	210 (90.9)	0.391
Proportion of tablets consumed under direct observation, median (IQR)	96 (88, 96)	92 (88, 96)	92 (88, 96)	96 (88, 100)	92 (88, 96)	0.186

[a] P-value from Kruskal-Wallis tests for continuous variables and Chi-square tests for categorical variables.

[b] Proportion of scheduled doses that were received.

[c] Post-hoc pairwise comparisons using Wilcoxon rank-sum tests showed no significant pairwise differences between groups after adjusting for multiple comparisons using the Holm test.

Table S11. Raw anthropometric parameters and additional anthropometric indicators for infants at 1 year of age, by supplementation group

	Prenatal; Postpartum Vitamin D Dose (IU/Week)										
	0; 0		4200; 0		16800; 0		28000; 0		28000; 28000		
	N	Mean ± SD/ n (%)	N	Mean ± SD/ n (%)	N	Mean ± SD/ n (%)	N	Mean ± SD/ n (%)	N	Mean ± SD/ n (%)	p[a]
**Raw anthropometric parameters**											
Crown-to-heel length, cm											
Boys	110	73.52 ± 2.66	129	72.92 ± 2.78	115	73.33 ± 2.51	119	73.26 ± 2.69	115	73.29 ± 2.39	0.504
Girls	119	71.79 ± 2.59	108	71.57 ± 2.75	122	71.83 ± 2.36	111	71.45 ± 2.62	116	72.07 ± 2.52	0.418
Weight, kg											
Boys	110	8.93 ± 1.09	129	8.63 ± 1.10	115	8.83 ± 1.13	118	8.74 ± 1.13	115	8.73 ± 1.04	0.286
Girls	118	8.19 ± 1.16	107	8.15 ± 1.14	122	8.19 ± 1.06	111	8.09 ± 1.06	116	8.21 ± 0.96	0.918
Head circumference, cm											
Boys	109	44.50 ± 1.33	128	44.43 ± 1.35	112	44.46 ± 1.25	119	44.56 ± 1.28	115	44.53 ± 1.27	0.934
Girls	116	43.56 ± 1.27	107	43.29 ± 1.15	122	43.35 ± 1.54	110	43.22 ± 1.16	116	43.25 ± 1.09	0.247
Upper-arm length, cm											
Boys	110	18.19 ± 0.86	129	17.91 ± 0.88	115	18.13 ± 0.90	119	18.00 ± 0.90	115	18.00 ± 0.84	0.095
Girls	119	17.67 ± 0.82	108	17.69 ± 0.95	122	17.60 ± 0.76	111	17.51 ± 0.75	116	17.53 ± 0.86	0.387
Mid-upper arm circumference, cm											
Boys	110	14.57 ± 1.07	129	14.32 ± 1.04	115	14.45 ± 1.07	119	14.32 ± 0.99	115	14.35 ± 0.98	0.288
Girls	119	14.06 ± 1.18	108	14.01 ± 1.03	122	14.01 ± 1.06	111	13.96 ± 1.00	116	14.06 ± 0.95	0.935
Rump-to-knee length, cm											
Boys	110	20.95 ± 1.15	129	20.68 ± 1.14	114	20.84 ± 1.10	118	20.66 ± 1.08	115	20.78 ± 0.91	0.236
Girls	117	20.69 ± 1.06	108	20.71 ± 1.18	122	20.55 ± 1.07	111	20.56 ± 0.97	116	20.62 ± 1.07	0.696
**Anthropometric indicators**											
Weight-for-age z-score <-2, n (%)	228	36 (15.8)	236	45 (19.1)	237	34 (14.3)	229	40 (17.5)	231	33 (14.3)	0.566
Head circumference-for-age z-score <-2, n (%)	225	35 (15.6)	235	49 (20.9)	234	41 (17.5)	229	46 (20.1)	231	48 (20.8)	0.518

[a] P-value from ANOVA test for continuous variables or Chi-square test for categorical variables.

Table S12. Pairwise between-group comparisons of the effect of different doses of prenatal and postpartum vitamin D supplementation on length-for-age z-score, weight-for-age z-score, weight-for-length z-score, body mass index-for-age z-score, head-circumference-for-age z-score, and mid-upper arm circumference-for-age z-score at 1 year of age

Anthropometric Index	Between-Group Comparisons		N_A	N_B	Mean difference, dose A vs. B (95% CI)	p[a]
	Dose A (IU/Week)	Dose B (IU/Week)				
Length-for-age z-score	4200; 0	0; 0	237	229	-0.18 (-0.38, 0.01)	0.069
	16800; 0	0; 0	237	229	-0.04 (-0.23, 0.14)	0.648
	16800; 0	4200; 0	237	237	0.14 (-0.05, 0.33)	0.145
	28000; 0	0; 0	230	229	-0.13 (-0.32, 0.07)	0.192
	28000; 0	16800; 0	230	237	-0.09 (-0.27, 0.10)	0.359
	28000; 28000	28000; 0	231	230	0.12 (-0.07, 0.31)	0.198
Weight-for-age z-score	4200; 0	0; 0	236	228	-0.19 (-0.40, 0.01)	0.068
	16800; 0	0; 0	237	228	-0.05 (-0.25, 0.15)	0.636
	16800; 0	4200; 0	237	236	0.14 (-0.06, 0.35)	0.162
	28000; 0	0; 0	229	228	-0.15 (-0.35, 0.05)	0.152
	28000; 0	16800; 0	229	237	-0.10 (-0.30, 0.10)	0.322
	28000; 28000	28000; 0	231	229	0.07 (-0.13, 0.26)	0.500
Weight-for-length z-score	4200; 0	0; 0	236	228	-0.13 (-0.32, 0.06)	0.178
	16800; 0	0; 0	237	228	-0.05 (-0.25, 0.15)	0.612
	16800; 0	4200; 0	237	236	0.08 (-0.11, 0.27)	0.405
	28000; 0	0; 0	229	228	-0.11 (-0.30, 0.08)	0.255
	28000; 0	16800; 0	229	237	-0.06 (-0.25, 0.13)	0.533
	28000; 28000	28000; 0	231	229	-0.01 (-0.19, 0.18)	0.944
Body mass index-for-age z-score	4200; 0	0; 0	236	228	-0.12 (-0.30, 0.07)	0.222
	16800; 0	0; 0	237	228	-0.04 (-0.24, 0.15)	0.649
	16800; 0	4200; 0	237	236	0.07 (-0.12, 0.26)	0.452
	28000; 0	0; 0	229	228	-0.10 (-0.29, 0.09)	0.301
	28000; 0	16800; 0	229	237	-0.05 (-0.24, 0.13)	0.571
	28000; 28000	28000; 0	231	229	-0.02 (-0.20, 0.16)	0.820
Head-circumference-for-age z-score	4200; 0	0; 0	235	225	-0.14 (-0.32, 0.04)	0.133
	16800; 0	0; 0	234	225	-0.10 (-0.28, 0.09)	0.306
	16800; 0	4200; 0	234	235	0.04 (-0.14, 0.22)	0.674
	28000; 0	0; 0	229	225	-0.11 (-0.28, 0.07)	0.239
	28000; 0	16800; 0	229	234	-0.01 (-0.19, 0.17)	0.929
	28000; 28000	28000; 0	231	229	-0.00 (-0.17, 0.16)	0.957
Mid-upper arm circumference-for-age z-score	4200; 0	0; 0	237	229	-0.13 (-0.31, 0.04)	0.124
	16800; 0	0; 0	237	229	-0.07 (-0.24, 0.10)	0.422
	16800; 0	4200; 0	237	237	0.06 (-0.10, 0.23)	0.452
	28000; 0	0; 0	230	229	-0.15 (-0.32, 0.02)	0.084

Anthropometric Index	Between-Group Comparisons		N_A	N_B	Mean difference, dose A vs. B (95% CI)	p[a]
	Dose A (IU/Week)	Dose B (IU/Week)				
	28000; 0	16800; 0	230	237	-0.08 (-0.24, 0.09)	0.348
	28000; 28000	28000; 0	231	230	0.06 (-0.10, 0.22)	0.445

[a] P-value from t-test.

Table S13. Anthropometric outcomes of infants at 1 year of age by supplementation group, after correcting for gestational age at birth

	Prenatal; Postpartum Vitamin D Dose (IU/Week)										
	N	0; 0	N	4200; 0	N	16800; 0	N	28000; 0	N	28000; 28000	p[a]
Gestational age-corrected age at measurement (days), median (min, max)	229	364 (311, 419)	237	364 (322, 415)	237	364 (321, 418)	230	364 (320, 419)	231	364 (317, 412)	0.776
Anthropometric indices, mean ± SD											
Length-for-age z-score	229	-0.88 ± 1.06	237	-1.08 ± 1.11	237	-0.93 ± 0.97	230	-1.02 ± 1.07	231	-0.90 ± 0.98	0.175
Weight-for-age z-score	229	-0.83 ± 1.26	237	-0.98 ± 1.14	237	-0.83 ± 1.09	230	-0.94 ± 1.09	231	-0.87 ± 1.03	0.506
Body mass index-for-age z-score	229	-0.42 ± 1.34	237	-0.48 ± 1.00	237	-0.41 ± 1.07	230	-0.48 ± 1.00	231	-0.49 ± 1.00	0.920
Head circumference-for-age z-score	229	-1.10 ± 0.99	237	-1.23 ± 0.96	237	-1.19 ± 1.04	230	-1.20 ± 0.92	231	-1.20 ± 0.89	0.641
Mid-upper arm circumference-for-age z-score	229	-0.13 ± 0.97	237	-0.27 ± 0.92	237	-0.21 ± 0.93	230	-0.28 ± 0.88	231	-0.22 ± 0.86	0.418
Stunted[b], n (%)		35 (15.3)		44 (18.6)		31 (13.1)		36 (15.7)		24 (10.4)	0.136

[a] P-value from ANOVA or Kruskal-Wallis tests for continuous variables and Chi-square test for categorical variables.

[b] Length-for-age z-score <-2. Denominator is number of infants with length-for-age z-scores.

Table S14. Per-protocol analysis of anthropometric outcomes of infants at 1 year of age, by supplementation group[a]

	Prenatal; Postpartum Vitamin D Dose (IU/Week)										
	N	0; 0	N	4200; 0	N	16800; 0	N	28000; 0	N	28000; 28000	p[b]
Age at measurement (days), median (min, max)	172	364 (364, 400)	161	365 (364, 415)	176	365 (364, 396)	151	364 (364, 419)	165	364 (364, 412)	0.422
**Raw anthropometric parameter, mean ± SD**											
Crown-to-heel length, cm											
Boys	85	73.5 ± 2.8	82	72.9 ± 2.8	88	73.2 ± 2.5	77	73.1 ± 2.6	83	73.0 ± 2.5	0.605
Girls	87	71.9 ± 2.6	79	71.5 ± 2.7	88	71.7 ± 2.5	74	71.3 ± 2.7	82	71.9 ± 2.5	0.547
Weight, kg											
Boys	85	8.94 ± 1.10	82	8.72 ± 1.16	88	8.80 ± 1.10	77	8.66 ± 1.14	83	8.65 ± 1.05	0.417
Girls	86	8.30 ± 1.23	78	8.15 ± 1.17	88	8.18 ± 1.08	74	8.01 ± 0.99	82	8.19 ± 0.87	0.562
Head circumference, cm											
Boys	84	44.5 ± 1.3	81	44.4 ± 1.4	86	44.5 ± 1.2	77	44.5 ± 1.2	83	44.5 ± 1.3	0.992
Girls	85	43.6 ± 1.2	78	43.3 ± 1.2	88	43.3 ± 1.6	74	43.1 ± 1.1	82	43.3 ± 1.1	0.141
Upper-arm length, cm											
Boys	85	18.2 ± 0.9	82	17.9 ± 1.0	88	18.1 ± 0.9	77	18.0 ± 0.9	83	17.9 ± 0.9	0.119
Girls	87	17.7 ± 0.8	79	17.7 ± 1.0	88	17.6 ± 0.8	74	17.5 ± 0.8	82	17.5 ± 0.9	0.394
Mid-upper arm circumference, cm											
Boys	85	14.6 ± 1.1	82	14.5 ± 1.1	88	14.4 ± 1.1	77	14.2 ± 1.0	83	14.3 ± 1.0	0.196
Girls	87	14.2 ± 1.2	79	13.9 ± 1.0	88	14.0 ± 1.0	74	14.0 ± 0.9	82	14.1 ± 0.9	0.663
Rump-to-knee length, cm											
Boys	85	21.0 ± 1.1	82	20.7 ± 1.2	88	20.8 ± 1.1	76	20.6 ± 1.1	83	20.7 ± 0.9	0.277
Girls	86	20.8 ± 1.1	79	20.7 ± 1.2	88	20.6 ± 1.0	74	20.5 ± 1.0	82	20.6 ± 1.0	0.495
**Anthropometric indices**											
Length-for-age z-score	172	-0.89 ± 1.08	161	-1.10 ± 1.10	176	-1.00 ± 1.00	151	-1.09 ± 1.06	165	-1.02 ± 1.02	0.392
Weight-for-age z-score	171	-0.75 ± 1.14	160	-0.94 ± 1.17	176	-0.87 ± 1.08	151	-1.03 ± 1.10	165	-0.94 ± 1.02	0.201
Weight-for-length z-score	171	-0.41 ± 1.08	160	-0.54 ± 1.06	176	-0.52 ± 1.05	151	-0.67 ± 1.01	165	-0.59 ± 1.00	0.241
Body mass index-for-age z-score	171	-0.30 ± 1.06	160	-0.41 ± 1.02	176	-0.40 ± 1.03	151	-0.55 ± 0.99	165	-0.48 ± 1.00	0.274
Head circumference-for-age z-score	169	-1.12 ± 0.98	159	-1.24 ± 1.01	174	-1.19 ± 1.07	151	-1.30 ± 0.89	165	-1.23 ± 0.92	0.550
Mid-upper arm circumference-for-age z-score	172	-0.08 ± 0.98	161	-0.24 ± 0.93	176	-0.22 ± 0.93	151	-0.32 ± 0.89	165	-0.26 ± 0.87	0.202
**Anthropometric indicators, n (%)**											
Stunted[c]		28 (16.3)		30 (18.6)		29 (16.5)		26 (17.2)		25 (15.2)	0.942
Wasted[d]		9 (5.3)		15 (9.4)		11 (6.3)		16 (10.6)		13 (7.9)	0.364

[a] Overall adherence ≥90% and non-study vitamin D/calcium supplement was not consumed.

[b] P-value from ANOVA or Kruskal-Wallis tests for continuous variables and Chi-square test for categorical variables.

[c] Length-for-age z-score <-2. Denominator is number of infants with length-for-age z-scores.

[d] Weight-for-length z-score <-2. Denominator is number of infants with weight-for-length z-scores.

Table S15. Length-for-age z-scores of infants at 1 year of age by supplementation group and stratified by gestational age at birth, infant sex, maternal height, maternal supplement adherence, or maternal baseline vitamin D status

	Prenatal; Postpartum Vitamin D Dose (IU/Week)											
	0; 0		4200; 0		16800; 0		28000; 0		28000; 28000			
	N	Mean ± SD	N	Mean ± SD	N	Mean ± SD	N	Mean ± SD	N	Mean ± SD	p_{group}[a]	p_{int}[b]
**Strata**												
Gestational age at birth												0.020
Preterm (<37 weeks)	21	-1.07 ± 1.19	18	-1.94 ± 1.20	25	-1.12 ± 1.19	24	-1.13 ± 1.25	19	-1.73 ± 1.29	0.074	
Term (≥37 weeks)	208	-0.92 ± 1.04	219	-1.05 ± 1.09	212	-0.96 ± 0.94	206	-1.05 ± 1.05	212	-0.87 ± 0.94	0.243	
Infant sex												0.739
Male	110	-0.98 ± 1.11	129	-1.23 ± 1.16	115	-1.06 ± 1.03	119	-1.09 ± 1.12	115	-1.08 ± 1.00	0.528	
Female	119	-0.88 ± 1.01	108	-0.98 ± 1.06	122	-0.89 ± 0.91	111	-1.03 ± 1.01	116	-0.79 ± 0.97	0.416	
Maternal height[c]												0.082
<151 cm	111	-1.20 ± 1.02	112	-1.31 ± 1.08	118	-1.21 ± 0.92	138	-1.33 ± 1.03	103	-1.41 ± 0.93	0.474	
≥151 cm	118	-0.68 ± 1.03	125	-0.94 ± 1.12	119	-0.74 ± 0.96	92	-0.66 ± 1.01	128	-0.55 ± 0.88	0.043	
Maternal supplement adherence												0.311
Not per-protocol[d]	57	-1.04 ± 0.98	76	-1.15 ± 1.16	61	-0.91 ± 0.88	79	-1.00 ± 1.09	66	-0.73 ± 0.91	0.153	
Per-protocol[e]	172	-0.89 ± 1.08	161	-1.10 ± 1.10	176	-1.00 ± 1.00	151	-1.09 ± 1.06	165	-1.02 ± 1.02	0.392	
Maternal baseline serum 25(OH)D concentration[f]												
<30 nmol/L cut-off												0.244
<30 nmol/L	137	-0.97 ± 1.03	145	-1.14 ± 1.12	146	-1.09 ± 0.99	150	-1.07 ± 0.98	159	-0.90 ± 1.04	0.263	
≥30 nmol/L	89	-0.88 ± 1.11	90	-1.06 ± 1.12	90	-0.78 ± 0.91	78	-1.02 ± 1.22	68	-1.07 ± 0.88	0.300	
<20 nmol/L cut-off												0.685
<20 nmol/L	72	-1.09 ± 1.08	83	-1.14 ± 1.04	76	-0.97 ± 1.03	80	-1.02 ± 0.96	83	-1.05 ± 1.03	0.876	
≥20 nmol/L	154	-0.86 ± 1.04	152	-1.09 ± 1.16	160	-0.97 ± 0.94	148	-1.07 ± 1.12	144	-0.90 ± 0.97	0.231	

[a] P-value for group differences within the stratum using ANOVA test.

[b] P-value for interaction between supplementation group and stratum using a linear regression model.

[c] Maternal height split at the median.

[d] Overall adherence <90 % and/or non-study vitamin D/calcium supplement was consumed.

[e] Overall adherence ≥90% and non-study vitamin D/calcium supplement was not consumed.

[f] No significant interaction between supplementation group and baseline maternal 25(OH)D concentration as a continuous variable (P = 0.682).

Table S16. Overall differences in length-for-age z-score at 1 year of age across supplementation groups after adjustment for individual baseline characteristics

Covariate	N	Mean difference in LAZ (95% CI)	$p_{covariate}$[a]	p_{group}[b]
None (unadjusted)	1164	-	-	0.230
Maternal age, year	1164	0.016 (0.002, 0.031)[c]	0.027	0.243
Gestational age at enrolment, weeks	1164	-0.030 (-0.061, 0.001)[c]	0.058	0.212
Marital status	1161			0.276
Married	1159	(ref)		
Not married	2	0.571 (-0.882, 2.025)	0.441	
Level of education	1164			0.172
No schooling	48	(ref)		
Primary incomplete	247	0.181 (-0.139, 0.501)	0.268	
Primary complete	163	0.069 (-0.264, 0.403)	0.684	
Secondary incomplete	446	0.238 (-0.070, 0.547)	0.130	
Secondary complete or higher	260	0.498 (0.179, 0.817)	0.002	
Primary occupation	1161			0.263
Homemaker	1086	(ref)		
Other	75	-0.058 (-0.302, 0.186)	0.641	
Asset index	1158			0.287
1 (lowest)	227	(ref)		
2	222	0.060 (-0.131, 0.251)	0.539	
3	238	0.216 (0.028, 0.403)	0.024	
4	235	0.350 (0.161, 0.538)	<0.001	
5 (highest)	236	0.492 (0.304, 0.680)	<0.001	
Gravidity	1164	0.024 (-0.033, 0.080)[c]	0.414	0.250
Parity	1164	-0.009 (-0.059, 0.041)[c]	0.723	0.225
Maternal height, cm	1164	0.067 (0.056, 0.077)[c]	<0.001	0.343
Maternal weight, kg	1164	0.023 (0.017, 0.029)[c]	<0.001	0.412
Month of enrolment	1164			0.226
March-May	420	(ref)		
June-August	369	0.085 (-0.061, 0.231)	0.255	
September-November	203	0.062 (-0.113, 0.238)	0.485	
December-February	172	0.049 (-0.137, 0.234)	0.605	
Hemoglobin, g/L	1164	0.001 (-0.004, 0.006)[c]	0.677	0.231
Baseline serum 25(OH)D concentration, nmol/L	1152	0.003 (-0.002, 0.007)[c]	0.248	0.318

[a] P-value for association between length-for-age z-score and the listed covariate from a linear regression model.

[b] P-value for overall differences in length-for-age z-scores across supplementation groups after adjustment for the single listed covariate from a linear regression model.

[c] Estimates represent mean difference in length-for-age z-score for a 1-unit increase in the covariate.

Table S17. Marginal effect of treatment allocation on length-for-age z-scores at 3-month time intervals from birth to 1 year of age[a]

Prenatal; Postpartum Vitamin D Dose (IU/Week)	Mean LAZ (95% CI)	Mean difference in LAZ (95% CI), vs. placebo (0; 0)				$p_{Overall}$[b]
	0; 0	4200; 0	16800; 0	28000; 0	28000; 28000	
Age						
Birth	-0.89 (-1.01, -0.77)	-0.08 (-0.26, 0.09)	-0.06 (-0.24, 0.11)	-0.13 (-0.31, 0.04)	0.02 (-0.15, 0.19)	0.385
3 Months	-0.83 (-0.94, -0.72)	-0.10 (-0.27, 0.06)	-0.02 (-0.18, 0.13)	-0.10 (-0.26, 0.05)	0.07 (-0.09, 0.23)	0.152
6 Months	-0.79 (-0.92, -0.66)	-0.12 (-0.31, 0.06)	0.00 (-0.17, 0.18)	-0.08 (-0.26, 0.10)	0.10 (-0.07, 0.28)	0.109
9 Months	-0.85 (-0.98, -0.72)	-0.14 (-0.33, 0.04)	-0.00 (-0.18, 0.17)	-0.09 (-0.27, 0.09)	0.06 (-0.12, 0.24)	0.174
12 Months	-0.93 (-1.06, -0.79)	-0.16 (-0.36, 0.04)	-0.02 (-0.20, 0.16)	-0.12 (-0.31, 0.08)	0.00 (-0.19, 0.19)	0.332
Overall	-0.86 (-0.97, -0.75)	-0.12 (-0.28, 0.04)	-0.03 (-0.18, 0.13)	-0.11 (-0.27, 0.05)	0.05 (-0.11, 0.21)	0.173

[a] Generalized estimating equations were used to model length-for-age z-score as a function of age. A restricted cubic spline model was used, in which knots were set at 91, 182 and 273 days of age (corresponding to 3, 6 and 9 months of age). Interaction terms between treatment group and all age terms were included in the model to allow for differences in slopes between treatment groups, and the marginal effects of treatment group on length-for-age z-scores at specified time points, as well as on overall length-for-age z-scores were assessed. N= 1211 infants contributed at least 1 observation to the model.

[b] Test of overall difference in means across treatment groups.

Table S18. Length-for-age z-scores of infants at 1 year of age by supplementation group, using multiple imputation to impute missing 1 year length-for-age z-scores[a]

Prenatal; Postpartum Vitamin D Dose (IU/Week)	Mean (95% CI)	Mean difference in LAZ (95% CI), vs. placebo (0; 0)				$p_{Overall}$[b]
	0; 0	4200; 0	16800; 0	28000; 0	28000; 28000	
Among all participants[c]	-0.95 (-1.08, -0.81)	-0.16 (-0.35, 0.03)	-0.02 (-0.21, 0.17)	-0.10 (-0.29, 0.09)	0.02 (-0.17, 0.21)	0.283
Among infants alive at 1 year of age[d]	-0.95 (-1.08, -0.81)	-0.16 (-0.35, 0.03)	-0.02 (-0.21, 0.17)	-0.10 (-0.29, 0.08)	0.01 (-0.18, 0.20)	0.297

[a] Auxiliary variables included in the imputation model were asset index, maternal height, infant LAZ at 6 months of age, infant LAZ at 9 months of age, and infant sex.

[b] Test of overall difference in means across treatment groups.

[c] 1298 complete datasets were generated.

[d] 1233 complete datasets were generated.

Table S19. Effect of prenatal and postpartum vitamin D on anthropometric outcomes at 1 year of age after correcting for gestational age at birth

Anthropometric Index	Between-Group Comparisons					
	Dose A (IU/Week)	Dose B (IU/Week)	N_A	N_B	Mean difference, dose A vs. B (95% CI)	p[a]
Length-for-age z-score	4200; 0	0; 0	237	229	-0.20 (-0.40, -0.00)	0.047
	16800; 0	0; 0	237	229	-0.04 (-0.23, 0.14)	0.658
	16800; 0	4200; 0	237	237	0.16 (-0.03, 0.35)	0.098
	28000; 0	0; 0	230	229	-0.13 (-0.33, 0.06)	0.187
	28000; 0	16800; 0	230	237	-0.09 (-0.28, 0.10)	0.343
	28000; 28000	28000; 0	231	230	0.12 (-0.07, 0.31)	0.219
Weight-for-age z-score	4200; 0	0; 0	237	229	-0.15 (-0.37, 0.06)	0.167
	16800; 0	0; 0	237	229	-0.01 (-0.22, 0.20)	0.931
	16800; 0	4200; 0	237	237	0.14 (-0.06, 0.35)	0.158
	28000; 0	0; 0	230	229	-0.11 (-0.33, 0.10)	0.302
	28000; 0	16800; 0	230	237	-0.10 (-0.30, 0.09)	0.302
	28000; 28000	28000; 0	231	230	0.07 (-0.13, 0.26)	0.498
Body mass index-for-age z-score	4200; 0	0; 0	237	229	-0.05 (-0.27, 0.16)	0.632
	16800; 0	0; 0	237	229	0.01 (-0.21, 0.23)	0.930
	16800; 0	4200; 0	237	237	0.06 (-0.12, 0.25)	0.513
	28000; 0	0; 0	230	229	-0.06 (-0.27, 0.16)	0.616
	28000; 0	16800; 0	230	237	-0.07 (-0.25, 0.12)	0.496
	28000; 28000	28000; 0	231	230	-0.01 (-0.19, 0.18)	0.931
Head-circumference-for-age z-score	4200; 0	0; 0	237	229	-0.13 (-0.31, 0.04)	0.141
	16800; 0	0; 0	237	229	-0.09 (-0.27, 0.10)	0.360
	16800; 0	4200; 0	237	237	0.05 (-0.13, 0.23)	0.608
	28000; 0	0; 0	230	229	-0.10 (-0.27, 0.08)	0.268
	28000; 0	16800; 0	230	237	-0.01 (-0.19, 0.17)	0.888
	28000; 28000	28000; 0	231	230	-0.00 (-0.17, 0.16)	0.953
Mid-upper arm circumference-for-age z-score	4200; 0	0; 0	237	229	-0.14 (-0.31, 0.04)	0.121
	16800; 0	0; 0	237	229	-0.07 (-0.24, 0.10)	0.423
	16800; 0	4200; 0	237	237	0.07 (-0.10, 0.23)	0.444
	28000; 0	0; 0	230	229	-0.15 (-0.32, 0.02)	0.084
	28000; 0	16800; 0	230	237	-0.08 (-0.24, 0.09)	0.347
	28000; 28000	28000; 0	231	230	0.06 (-0.10, 0.22)	0.448

[a] P-value from t-test.

Table S20. Per-protocol analysis of the effect of prenatal and postpartum vitamin D supplementation on anthropometric outcomes at 1 year of age[a]

Anthropometric Index	Between-Group Comparisons		N_A	N_B	Mean difference, dose A vs. B (95% CI)	p[b]
	Dose A (IU/Week)	Dose B (IU/Week)				
Length-for-age z-score	4200; 0	0; 0	210	215	-0.18 (-0.38, 0.03)	0.093
	16800; 0	0; 0	214	215	-0.06 (-0.25, 0.14)	0.553
	16800; 0	4200; 0	214	210	0.12 (-0.08, 0.32)	0.245
	28000; 0	0; 0	201	215	-0.13 (-0.33, 0.08)	0.215
	28000; 0	16800; 0	201	214	-0.07 (-0.27, 0.13)	0.483
	28000; 28000	28000; 0	174	154	0.10 (-0.12, 0.32)	0.378
Weight-for-age z-score	4200; 0	0; 0	209	214	-0.21 (-0.42, 0.01)	0.056
	16800; 0	0; 0	214	214	-0.08 (-0.29, 0.13)	0.438
	16800; 0	4200; 0	214	209	0.13 (-0.09, 0.34)	0.248
	28000; 0	0; 0	200	214	-0.17 (-0.38, 0.05)	0.123
	28000; 0	16800; 0	200	214	-0.08 (-0.30, 0.13)	0.437
	28000; 28000	28000; 0	174	154	0.11 (-0.12, 0.34)	0.340
Weight-for-length z-score	4200; 0	0; 0	209	214	-0.16 (-0.35, 0.04)	0.111
	16800; 0	0; 0	214	214	-0.09 (-0.29, 0.12)	0.399
	16800; 0	4200; 0	214	209	0.07 (-0.13, 0.27)	0.480
	28000; 0	0; 0	200	214	-0.14 (-0.34, 0.06)	0.169
	28000; 0	16800; 0	200	214	-0.05 (-0.26, 0.15)	0.617
	28000; 28000	28000; 0	174	154	0.08 (-0.14, 0.30)	0.484
Body mass index-for-age z-score	4200; 0	0; 0	209	214	-0.15 (-0.34, 0.05)	0.135
	16800; 0	0; 0	214	214	-0.08 (-0.28, 0.12)	0.421
	16800; 0	4200; 0	214	209	0.06 (-0.13, 0.26)	0.525
	28000; 0	0; 0	200	214	-0.13 (-0.32, 0.07)	0.197
	28000; 0	16800; 0	200	214	-0.05 (-0.25, 0.15)	0.652
	28000; 28000	28000; 0	174	154	0.07 (-0.15, 0.28)	0.545
Head-circumference-for-age z-score	4200; 0	0; 0	208	212	-0.16 (-0.35, 0.03)	0.098
	16800; 0	0; 0	211	212	-0.12 (-0.31, 0.08)	0.242
	16800; 0	4200; 0	211	208	0.04 (-0.15, 0.24)	0.667
	28000; 0	0; 0	201	212	-0.14 (-0.32, 0.05)	0.143
	28000; 0	16800; 0	201	211	-0.02 (-0.21, 0.17)	0.819
	28000; 28000	28000; 0	174	154	0.07 (-0.13, 0.27)	0.497
Mid-upper arm circumference-for-age z-score	4200; 0	0; 0	210	215	-0.17 (-0.35, 0.01)	0.066
	16800; 0	0; 0	214	215	-0.10 (-0.28, 0.09)	0.301
	16800; 0	4200; 0	214	210	0.07 (-0.11, 0.25)	0.426
	28000; 0	0; 0	201	215	-0.16 (-0.33, 0.02)	0.079
	28000; 0	16800; 0	201	214	-0.06 (-0.24, 0.11)	0.482
	28000; 28000	28000; 0	174	154	0.10 (-0.09, 0.29)	0.287

[a] Adherence ≥90% and non-study vitamin D/calcium supplement was not consumed. Defined in the prenatal period for comparisons looking at the effect of prenatal vitamin D supplementation (4200; 0 vs. 0; 0; 16800; 0 vs. 0; 0; 16800; 0 vs. 4200; 0; 28000; 0 vs. 0; 0; 28000; 0 vs. 16800; 0), and in the postpartum period for comparison looking at the effect of postpartum supplementation (28000; 28000 vs. 28000; 0).
[b] P-value from t-test.

Table S21. Effect of prenatal and postpartum vitamin D on length-for-age z-score at 1 year of age by gestational age at birth, infant sex, maternal height, maternal supplement adherence, and maternal baseline serum 25(OH)D concentration

Strata	Between-Group Comparison		N_A	N_B	Mean difference in LAZ (95% CI)	N_A	N_B	Mean difference in LAZ (95% CI)	p_{int} [a]
	Dose A	Dose B							
**Gestational age at birth**					**Preterm**			**Term**	
	4200; 0	0; 0	18	21	-0.87 (-1.65, -0.10)	219	208	-0.13 (-0.33, 0.07)	0.040
	16800; 0	0; 0	25	21	-0.05 (-0.76, 0.66)	212	208	-0.04 (-0.23, 0.15)	0.974
	16800; 0	4200; 0	25	18	0.82 (0.08, 1.57)	212	219	0.09 (-0.10, 0.28)	0.029
	28000; 0	0; 0	24	21	-0.06 (-0.80, 0.68)	206	208	-0.14 (-0.34, 0.07)	0.826
	28000; 0	16800; 0	24	25	-0.01 (-0.72, 0.69)	206	212	-0.10 (-0.29, 0.10)	0.787
	28000; 28000	28000; 0	19	24	-0.60 (-1.39, 0.19)	212	206	0.19 (-0.00, 0.38)	0.017
**Infant sex**					**Males**			**Females**	
	4200; 0	0; 0	129	110	-0.24 (-0.53, 0.05)	108	119	-0.10 (-0.37, 0.17)	0.470
	16800; 0	0; 0	115	110	-0.08 (-0.36, 0.20)	122	119	-0.01 (-0.25, 0.24)	0.705
	16800; 0	4200; 0	115	129	0.16 (-0.11, 0.44)	122	108	0.09 (-0.17, 0.35)	0.698
	28000; 0	0; 0	119	110	-0.10 (-0.39, 0.19)	111	119	-0.15 (-0.41, 0.11)	0.823
	28000; 0	16800; 0	119	115	-0.03 (-0.30, 0.25)	111	122	-0.14 (-0.39, 0.11)	0.541
	28000; 28000	28000; 0	115	119	0.01 (-0.27, 0.28)	116	111	0.24 (-0.02, 0.50)	0.231
**Maternal height**					**<151 cm**			**≥151 cm**	
	4200; 0	0; 0	112	111	-0.11 (-0.39, 0.16)	125	118	-0.26 (-0.53, 0.02)	0.468
	16800; 0	0; 0	118	111	-0.01 (-0.26, 0.24)	119	118	-0.06 (-0.32, 0.19)	0.770
	16800; 0	4200; 0	118	112	0.10 (-0.16, 0.37)	119	125	0.19 (-0.07, 0.46)	0.633
	28000; 0	0; 0	138	111	-0.13 (-0.39, 0.13)	92	118	0.02 (-0.26, 0.30)	0.437
	28000; 0	16800; 0	138	118	-0.12 (-0.36, 0.12)	92	119	0.08 (-0.19, 0.35)	0.269
	28000; 28000	28000; 0	103	138	-0.08 (-0.34, 0.17)	128	92	0.11 (-0.15, 0.36)	0.297
**Maternal supplement adherence**[b]					**Not per-protocol**			**Per-protocol**	
	4200; 0	0; 0	27	14	-0.22 (-0.98, 0.54)	210	215	-0.18 (-0.38, 0.03)	0.907
	16800; 0	0; 0	23	14	0.11 (-0.51, 0.74)	214	215	-0.06 (-0.25, 0.14)	0.634
	16800; 0	4200; 0	23	27	0.33 (-0.26, 0.93)	214	210	0.12 (-0.08, 0.32)	0.497
	28000; 0	0; 0	29	14	-0.12 (-0.85, 0.61)	201	215	-0.13 (-0.33, 0.08)	0.984
	28000; 0	16800; 0	29	23	-0.23 (-0.80, 0.34)	201	214	-0.07 (-0.27, 0.13)	0.590
	28000; 28000	28000; 0	57	76	0.21 (-0.16, 0.57)	174	154	0.10 (-0.12, 0.32)	0.617
**Maternal baseline vitamin D status**					**<30 nmol/L**			**≥30 nmol/L**	
	4200; 0	0; 0	145	137	-0.18 (-0.43, 0.08)	90	89	-0.17 (-0.50, 0.16)	0.989
	16800; 0	0; 0	146	137	-0.12 (-0.36, 0.11)	90	89	0.10 (-0.19, 0.40)	0.242
	16800; 0	4200; 0	146	145	0.05 (-0.19, 0.30)	90	90	0.28 (-0.02, 0.58)	0.259
	28000; 0	0; 0	150	137	-0.10 (-0.33, 0.13)	78	89	-0.14 (-0.50, 0.21)	0.840

Strata	Between-Group Comparison		N_A	N_B	Mean difference in LAZ (95% CI)	N_A	N_B	Mean difference in LAZ (95% CI)	p_{int} [a]
	Dose A	Dose B							
	28000; 0	16800; 0	150	146	0.02 (-0.20, 0.25)	78	90	-0.25 (-0.57, 0.08)	0.173
	28000; 28000	28000; 0	159	150	0.16 (-0.06, 0.39)	68	78	-0.05 (-0.40, 0.31)	0.314

[a] P-value for interaction between supplementation group and stratum using a linear regression model.

[b] Adherence ≥90% and non-study vitamin D/calcium supplement was not consumed. Defined in the prenatal period for comparisons looking at the effect of prenatal vitamin D supplementation (4200; 0 vs. 0; 0; 16800; 0 vs. 0; 0; 16800; 0 vs. 4200; 0; 28000; 0 vs. 0; 0; 28000; 0 vs. 16800; 0), and in the postpartum period for comparison looking at the effect of postpartum supplementation (28000; 28000 vs. 28000; 0).

Table S22. Effect of prenatal and postpartum vitamin D on length-for-age z-score at 1 year of age after adjustment for a single baseline characteristic

	Prenatal Vitamin D Dose (IU/Week)						Postpartum Vitamin D Dose (IU/Week)	
	4200; 0 vs. 0; 0		16800; 0 vs. 0; 0		28000; 0 vs. 0; 0		28000; 28000 vs. 28000; 0	
**Covariate**	**Mean difference in LAZ (95% CI)**	**p[a]**	**Mean difference in LAZ (95% CI)**	**p[a]**	**Mean difference in LAZ (95% CI)**	**p[a]**	**Mean difference in LAZ (95% CI)**	**p[a]**
None (unadjusted)	-0.18 (-0.38, 0.01)	0.069	-0.04 (-0.23, 0.14)	0.648	-0.13 (-0.32, 0.07)	0.192	0.12 (-0.07, 0.31)	0.198
Maternal age	-0.18 (-0.37, 0.02)	0.082	-0.03 (-0.22, 0.15)	0.728	-0.12 (-0.32, 0.07)	0.213	0.12 (-0.07, 0.31)	0.198
Gestational age at enrolment	-0.20 (-0.39, 0.00)	0.053	-0.05 (-0.23, 0.14)	0.632	-0.13 (-0.33, 0.06)	0.180	0.12 (-0.07, 0.31)	0.199
Marital status	-0.18 (-0.38, 0.02)	0.078	-0.04 (-0.22, 0.15)	0.687	-0.11 (-0.31, 0.08)	0.249	0.11 (-0.08, 0.30)	0.255
Level of education	-0.19 (-0.39, 0.01)	0.061	-0.05 (-0.23, 0.13)	0.594	-0.13 (-0.32, 0.07)	0.201	0.12 (-0.07, 0.30)	0.227
Primary occupation	-0.18 (-0.38, 0.01)	0.069	-0.04 (-0.23, 0.14)	0.648	-0.12 (-0.31, 0.07)	0.227	0.11 (-0.08, 0.30)	0.261
Asset Index	-0.19 (-0.39, 0.01)	0.060	-0.06 (-0.24, 0.12)	0.503	-0.12 (-0.31, 0.07)	0.218	0.08 (-0.11, 0.26)	0.413
Gravidity	-0.18 (-0.38, 0.02)	0.081	-0.04 (-0.23, 0.14)	0.657	-0.12 (-0.32, 0.07)	0.214	0.12 (-0.07, 0.31)	0.202
Parity	-0.18 (-0.38, 0.02)	0.070	-0.04 (-0.23, 0.14)	0.654	-0.13 (-0.32, 0.07)	0.193	0.13 (-0.06, 0.31)	0.194
Maternal height	-0.17 (-0.36, 0.02)	0.074	-0.02 (-0.20, 0.16)	0.824	-0.06 (-0.25, 0.12)	0.518	-0.01 (-0.18, 0.17)	0.941
Maternal weight	-0.16 (-0.35, 0.04)	0.116	-0.03 (-0.21, 0.15)	0.740	-0.10 (-0.29, 0.09)	0.315	0.08 (-0.10, 0.27)	0.385
Season of enrolment	-0.18 (-0.38, 0.02)	0.071	-0.05 (-0.23, 0.14)	0.633	-0.13 (-0.32, 0.07)	0.193	0.12 (-0.07, 0.31)	0.208
Hemoglobin	-0.19 (-0.39, 0.01)	0.060	-0.04 (-0.23, 0.14)	0.668	-0.13 (-0.32, 0.07)	0.193	0.13 (-0.06, 0.32)	0.192
Baseline serum 25(OH)D concentration	-0.18 (-0.38, 0.02)	0.084	-0.04 (-0.22, 0.15)	0.681	-0.12 (-0.31, 0.08)	0.237	0.10 (-0.09, 0.29)	0.308

[a] P-value for difference between groups after adjustment for a baseline characteristic from a linear regression model.

Table S23. Maternal, venous cord and infant 25-hydroxyvitamin D concentrations, by supplementation group

	Prenatal; Postpartum Dose (IU/Week)					
	0; 0	4200; 0	16800; 0	28000; 0	28000; 28000	p[a]
**Maternal Baseline**						
N	254	258	259	258	256	–
Gestational age (days), median (IQR)	143 (133-156)	141 (131-154)	142 (131-155)	143 (131-154)	141 (131-154)	–
25(OH)D (nmol/L), mean ± SD	27.70 ± 13.80	27.41 ± 14.33	28.69 ± 14.01	26.96 ± 14.72	26.59 ± 13.24	0.498
<30 nmol/L, n (%)	154 (60.6)	161 (62.4)	157 (60.6)	171 (66.3)	176 (68.8)	0.210
≥30 – <50 nmol/L, n (%)	85 (33.5)	85 (32.9)	77 (29.7)	70 (27.1)	61 (23.8)	0.089
>125 nmol/L, n (%)	0 (0.0)	0 (0.0)	0 (0.0)	0 (0.0)	0 (0.0)	–
**Maternal Delivery[f]**						
N	132	126	138	125	135	–
Gestational age (days), median (IQR)	274 (268-280)	274 (267-280)	273 (266-279)	274 (266-281)	276 (270-282)	–
25(OH)D (nmol/L), mean ± SD	24.30 ± 15.96	69.34 ± 19.44 [b]	100.37 ± 23.80 [b,c]	111.20 ± 27.83 [b,c,d]	113.49 ± 25.49 [b,c,d]	<0.001
<30 nmol/L, n (%)	100 (75.8)	2 (1.6) [b]	0 (0.0) [b]	1 (0.8) [b]	0 (0.0) [b]	<0.001
≥30 – <50 nmol/L, n (%)	26 (19.7)	13 (10.3) [b,c]	2 (1.4) [b,c]	1 (0.8) [b,c]	0 (0.0) [b,c]	<0.001
>125 nmol/L, n (%)	0 (0.0)	2 (1.6) [b,c]	17 (12.3) [b,c]	39 (31.2) [b,c]	45 (33.3) [b,c,d]	<0.001
**Maternal 3 Months Postpartum[g]**						
N	114	112	126	111	118	–
Postnatal age (days), median (IQR)	91 (91-92)	91 (91-92)	91 (91-92)	91 (91-92)	91 (91-92)	–
25(OH)D (nmol/L), mean ± SD	27.38 ± 12.70	31.72 ± 10.57 [b]	51.47 ± 11.69 [b,c]	59.97 ± 13.60 [b,c,d]	99.60 ± 20.60 [b,c,d,e]	<0.001
<30 nmol/L, n (%)	73 (64.0)	55 (49.1)	2 (1.6) [b,c]	0 (0.0) [b,c]	0 (0.0) [b,c]	<0.001
≥30 – <50 nmol/L, n (%)	36 (31.6)	50 (44.6)	60 (47.6) [b]	28 (25.2) [c,d]	0 (0.0) [b,c,d,e]	<0.001
>125 nmol/L, n (%)	0 (0.0)	0 (0.0)	0 (0.0)	0 (0.0)	16 (13.6) [b,c,d,e]	<0.001
**Maternal 6 Months Postpartum[h]**						
N	114	118	128	111	119	–
Postnatal age (days), median (IQR)	182 (182-183)	182 (182-183)	182 (182-183)	182 (182-183)	182 (182-183)	–
25(OH)D (nmol/L), mean ± SD	29.87 ± 12.84	30.53 ± 11.26	45.01 ± 12.02 [b,c]	51.97 ± 12.83 [b,c,d]	103.83 ± 23.27 [b,c,d,e]	<0.001
<30 nmol/L, n (%)	65 (57.0)	64 (54.2)	14 (10.9) [b,c]	4 (3.6) [b,c]	0 (0.0) [b,c,d]	<0.001
≥30 – <50 nmol/L, n (%)	40 (35.1)	48 (40.7)	75 (58.6) [b,c]	49 (44.1)	1 (0.8) [b,c,d,e]	<0.001
>125 nmol/L, n (%)	0 (0.0)	0 (0.0)	0 (0.0)	0 (0.0)	23 (19.3) [b,c,d,e]	<0.001
**Venous Cord**						
N	99	102	110	97	99	–
Gestational age (days), median (IQR)	274 (268-281)	275 (267-281)	273 (266-279)	275 (266-282)	277 (272-282)	–
25(OH)D (nmol/L), mean ± SD	11.93 ± 7.37	37.25 ± 10.39 [b]	59.93 ± 12.99 [b,c]	72.30 ± 16.24 [b,c,d]	70.15 ± 16.32 [b,c,d]	<0.001
<30 nmol/L, n (%)	97 (98.0)	22 (21.6) [b]	0 (0.0) [b,c]	0 (0.0) [b,c]	0 (0.0) [b,c]	<0.001
≥30 – <50 nmol/L, n (%)	2 (2.0)	71 (69.6) [b]	32 (29.1) [b,c]	3 (3.1) [c,d]	9 (9.1) [c,d]	<0.001
>125 nmol/L, n (%)	0 (0.0)	0 (0.0)	0 (0.0)	1 (1.0)	0 (0.0)	0.191
**Infants at 3 Months[i]**						
N	73	63	65	69	75	–
Postnatal age (days), median (IQR)	91 (91-92)	91 (91-92)	91 (91-92)	91 (91-92)	91 (91-92)	–
25(OH)D (nmol/L), mean ± SD	30.22 ± 21.88	33.03 ± 20.24	37.17 ± 21.40	38.26 ± 19.52	75.20 ± 17.19 [b,c,d,e]	<0.001
<30 nmol/L, n (%)	43 (58.9)	35 (55.6)	30 (46.2)	29 (42.0)	0 (0.0) [b,c,d,e]	<0.001
≥30 – <50 nmol/L, n (%)	13 (17.8)	15 (23.8)	16 (24.6)	24 (34.8)	5 (6.7) [b,c,d,e]	0.001
>125 nmol/L, n (%)	0 (0.0)	0 (0.0)	0 (0.0)	0 (0.0)	1 (1.3)	0.999

**Infants at 6 Months**[j]						
N	51	47	54	53	49	–
Postnatal age (days), median (IQR)	182 (182-183)	182 (182-183)	182 (182-183)	182 (182-183)	182 (182-183)	–
25(OH)D (nmol/L), mean ± SD	47.81 ± 23.70	48.09 ± 22.50	49.55 ± 25.16	46.80 ± 26.43	78.96 ± 22.46 [b,c,d,e]	<0.001
<30 nmol/L, n (%)	12 (23.5)	11 (23.4)	12 (22.2)	14 (26.4)	1 (2.0) [b,c,d,e]	0.006
≥30 – <50 nmol/L, n (%)	13 (25.5)	13 (27.7)	18 (33.3)	17 (32.1)	4 (8.3) [d,e]	0.033
>125 nmol/L, n (%)	0 (0.0)	0 (0.0)	0 (0.0)	0 (0.0)	2 (4.2)	0.069
**Infants at 12 Months**[k]						
N	34	35	34	38	41	–
Postnatal age (days), median (IQR)	364 (364-365)	364 (364-365)	364 (364-367)	365 (364-368)	364 (364-365)	–
25(OH)D (nmol/L), mean ± SD	47.61 ± 19.49	52.36 ± 21.74	53.19 ± 20.52	49.36 ± 19.30	57.10 ± 20.02	0.293
<30 nmol/L, n (%)	6 (17.6)	5 (14.3)	5 (14.7)	7 (18.4)	2 (4.9)	0.351
≥30 – <50 nmol/L, n (%)	17 (50.0)	10 (28.6)	9 (26.5)	14 (36.8)	14 (34.1)	0.273
>125 nmol/L, n (%)	0 (0.0)	0 (0.0)	0 (0.0)	0 (0.0)	0 (0.0)	–

[a] P-value from ANOVA for continuous variables and Chi-square or Fischer's tests for categorical variables.

[b] Post-hoc pairwise comparisons using t-tests, Chi-square tests or Fischer's tests showed significant pairwise difference from placebo group, after adjusting for multiple comparisons using the Holm test.

[c] Post-hoc pairwise comparisons using t-tests, Chi-square tests or Fischer's tests showed significant pairwise difference from group receiving 4200; 0, after adjusting for multiple comparisons using the Holm test.

[d] Post-hoc pairwise comparisons using t-tests, Chi-square tests or Fischer's tests showed significant pairwise difference from group receiving 16800; 0, after adjusting for multiple comparisons using the Holm test.

[e] Post-hoc pairwise comparisons using t-tests, Chi-square tests or Fischer's tests showed significant pairwise difference from group receiving 28000; 0, after adjusting for multiple comparisons using the Holm test.

[f] Measurements were excluded if they were collected more than 3 weeks before or after delivery. $N_{4200; 0}$ = 1, $N_{16800; 0}$ = 1, and $N_{28000; 28000}$ = 1 measurements excluded.

[g] Measurements were excluded if they were collected >136 days (~4.5 months) postpartum. $N_{28000; 28000}$ = 2 measurements excluded.

[h] Measurements were excluded if they were collected >227 days (~7.5 months) postpartum. $N_{0; 0}$ = 2, $N_{4200; 0}$ = 1, $N_{16800; 0}$ = 1, and $N_{28000; 28000}$ = 1 measurements excluded.

[i] Measurements were excluded if they were collected at >136 days (~4.5 months) of age. $N_{4200; 0}$ = 1, $N_{16800; 0}$ = 1, and $N_{28000; 28000}$ = 2 measurements excluded.

[j] Measurements were excluded if they were collected at >227 days (~7.5 months) of age. $N_{0; 0}$ = 9, $N_{4200; 0}$ = 10, $N_{16800; 0}$= 11, $N_{28000; 0}$ = 8, and $N_{28000; 28000}$ = 8 measurements excluded.

[k] Measurements were excluded if they were collected at >410 days (~13.5 months) of age. $N_{16800; 0}$= 1 measurement excluded.

Table S24. Maternal, venous cord and infant C-3 epimer of 25-hydroxyvitamin D3 and total 25(OH)D3[a], by supplementation group

	Prenatal; Postpartum Dose (IU/Week)									
	0; 0		4200; 0		16800; 0		28000; 0		28000; 28000	
	C-3 Epimer	Total 25(OH)D	C-3 Epimer	Total 25(OH)D	C-3 Epimer	Total 25(OH)D	C-3 Epimer	Total 25(OH)D	C-3 Epimer	Total 25(OH)D
**Maternal Baseline**										
N	254	254	258	258	259	259	258	258	256	256
25(OH)D (nmol/L), mean ± SD	0.91 ± 0.71	28.63 ± 14.24	0.86 ± 0.59	28.27 ± 14.68	0.91 ± 0.60	29.60 ± 14.43	0.90 ± 0.72	27.86 ± 15.21	0.91 ± 0.70	27.50 ± 13.69
<30 nmol/L, n (%)	–	150 (59.3)	–	157 (60.9)	–	153 (59.1)	–	160 (62.0)	–	169 (66.0)
≥30 – <50 nmol/L, n (%)	–	88 (34.8)	–	84 (32.6)	–	77 (29.7)	–	80 (31.0)	–	65 (25.4)
>125 nmol/L, n (%)	–	0 (0.0)	–	0 (0.0)	–	0 (0.0)	–	0 (0.0)	–	0 (0.0)
**Maternal Delivery[b]**										
N	132	132	126	126	138	138	125	125	135	135
25(OH)D (nmol/L), mean ± SD	0.88 ± 0.91	25.18 ± 16.68	2.77 ± 1.38	72.11 ± 20.12	6.07 ± 2.50	106.44 ± 24.95	8.59 ± 4.38	119.79 ± 30.00	8.22 ± 3.87	121.71 ± 27.14
<30 nmol/L, n (%)	–	96 (72.7)	–	1 (0.8)	–	0 (0.0)	–	1 (0.8)	–	0 (0.0)
≥30 – <50 nmol/L, n (%)	–	29 (22.0)	–	12 (9.5)	–	2 (1.4)	–	1 (0.8)	–	0 (0.0)
>125 nmol/L, n (%)	–	0 (0.0)	–	2 (1.6)	–	33 (23.9)	–	50 (40.0)	–	56 (41.5)
**Maternal 3 Months Postpartum[c]**										
N	114	114	112	112	126	126	111	111	118	118
25(OH)D (nmol/L), mean ± SD	0.57 ± 0.54	27.95 ± 13.00	0.61 ± 0.47	32.33 ± 10.81	0.93 ± 0.67	52.41 ± 11.97	1.23 ± 0.77	61.20 ± 13.87	3.39 ± 1.69	102.99 ± 21.03
<30 nmol/L, n (%)	–	71 (62.3)	–	52 (46.4)	–	2 (1.6)	–	0 (0.0)	–	0 (0.0)
≥30 – <50 nmol/L, n (%)	–	37 (32.5)	–	50 (44.6)	–	58 (46.0)	–	23 (20.7)	–	0 (0.0)
>125 nmol/L, n (%)	–	0 (0.0)	–	0 (0.0)	–	0 (0.0)	–	0 (0.0)	–	20 (16.9)
**Maternal 6 Months Postpartum[d]**										
N	114	114	118	118	128	128	111	111	119	119
25(OH)D (nmol/L), mean ± SD	0.58 ± 0.60	30.44 ± 13.19	0.58 ± 0.52	31.11 ± 11.53	0.86 ± 0.60	45.87 ± 12.21	1.09 ± 0.82	53.06 ± 13.06	3.74 ± 1.92	107.57 ± 24.22
<30 nmol/L, n (%)	–	63 (55.3)	–	60 (50.8)	–	12 (9.4)	–	3 (2.7)	–	0 (0.0)
≥30 – <50 nmol/L, n (%)	–	41 (36.0)	–	52 (44.1)	–	77 (60.2)	–	43 (38.7)	–	1 (0.8)
>125 nmol/L, n (%)	–	0 (0.0)	–	0 (0.0)	–	0 (0.0)	–	0 (0.0)	–	24 (20.2)
**Venous Cord**										
N	99	99	102	102	110	110	97	97	99	99
25(OH)D (nmol/L), mean ± SD	0.92 ± 0.69	12.85 ± 7.90	3.33 ± 1.54	40.58 ± 11.40	7.43 ± 2.51	67.36 ± 14.51	10.61 ± 4.24	82.91 ± 18.62	9.99 ± 2.90	80.14 ± 17.92
<30 nmol/L, n (%)	–	95 (96.0)	–	13 (12.7)	–	0 (0.0)	–	0 (0.0)	–	0 (0.0)
≥30 – <50 nmol/L, n (%)	–	4 (4.0)	–	79 (77.5)	–	14 (12.7)	–	2 (2.1)	–	4 (4.0)
>125 nmol/L, n (%)	–	0 (0.0)	–	0 (0.0)	–	0 (0.0)	–	3 (3.1)	–	2 (2.0)
**Infants at 3 Months[e]**										
N	73	73	63	63	65	65	69	69	75	75
25(OH)D (nmol/L), mean ± SD	3.22 ± 2.66	33.44 ± 24.18	4.05 ± 3.90	37.09 ± 23.26	4.41 ± 3.00	41.59 ± 23.86	5.09 ± 3.22	43.35 ± 22.01	14.11 ± 6.38	89.31 ± 21.09
<30 nmol/L, n (%)	–	40 (54.8)	–	28 (44.4)	–	26 (40.0)	–	21 (30.4)	–	0 (0.0)
≥30 – <50 nmol/L, n (%)	–	12 (16.4)	–	17 (27.0)	–	17 (26.2)	–	25 (36.2)	–	2 (2.7)

>125 nmol/L, n (%)	–	0 (0.0)	–	0 (0.0)	–	0 (0.0)	–	0 (0.0)	–	3 (4.0)
**Infants at 6 Months[f]**										
N	51	51	47	47	54	54	53	53	49	49
25(OH)D (nmol/L), mean ± SD	3.79 ± 2.41	51.59 ± 25.56	4.30 ± 2.98	52.39 ± 24.94	3.83 ± 2.68	53.38 ± 26.98	3.51 ± 2.64	50.31 ± 28.70	8.35 ± 4.86	88.37 ± 24.57
<30 nmol/L, n (%)	–	10 (19.6)	–	8 (17.0)	–	12 (22.2)	–	13 (24.5)	–	0 (0.0)
≥30 – <50 nmol/L, n (%)	–	12 (23.5)	–	13 (27.7)	–	14 (25.9)	–	16 (30.2)	–	2 (4.2)
>125 nmol/L, n (%)	–	0 (0.0)	–	0 (0.0)	–	0 (0.0)	–	0 (0.0)	–	3 (6.3)
**Infants at 12 Months[g]**										
N	34	34	35	35	34	34	38	38	41	41
25(OH)D (nmol/L), mean ± SD	2.71 ± 1.52	50.32 ± 20.77	3.18 ± 2.18	55.55 ± 23.08	3.50 ± 2.48	56.69 ± 22.29	3.00 ± 2.26	52.36 ± 20.89	3.04 ± 1.51	60.14 ± 21.28
<30 nmol/L, n (%)	–	5 (14.7)	–	5 (14.3)	–	4 (11.8)	–	7 (18.4)	–	1 (2.4)
≥30 – <50 nmol/L, n (%)	–	18 (52.9)	–	9 (25.7)	–	9 (26.5)	–	11 (28.9)	–	13 (31.7)
>125 nmol/L, n (%)	–	0 (0.0)	–	0 (0.0)	–	0 (0.0)	–	0 (0.0)	–	1 (2.4)

[a] Total 25(OH)D3 reflects the sum of 25(OH)D3 concentrations and its C-3 epimer.

[b] Measurements were excluded if they were collected more than 3 weeks before or after delivery. $N_{4200;\,0}$ = 1, $N_{16800;\,0}$ = 1, and $N_{28000;\,28000}$ = 1 measurements excluded.

[c] Measurements were excluded if they were collected >136 days (~4.5 months) postpartum. $N_{28000;\,28000}$ = 2 measurements excluded.

[d] Measurements were excluded if they were collected >227 days (~7.5 months) postpartum. $N_{0;\,0}$ = 2, $N_{4200;\,0}$ = 1, $N_{16800;\,0}$ = 1, and $N_{28000;\,28000}$ = 1 measurements excluded.

[e] Measurements were excluded if they were collected at >136 days (~4.5 months) of age. $N_{4200;\,0}$ = 1, $N_{16800;\,0}$ = 1, and $N_{28000;\,28000}$ = 2 measurements excluded.

[f] Measurements were excluded if they were collected at >227 days (~7.5 months) of age. $N_{0;\,0}$ = 2, $N_{4200;\,0}$ = 1, $N_{16800;\,0}$= 1, and $N_{28000;\,28000}$ = 2 measurements excluded.

[g] Measurements were excluded if they were collected at >410 days (~13.5 months) of age. $N_{16800;\,0}$= 1 measurement excluded.

Table S25. Maternal and infant biochemical measures and adverse events, by supplementation group

	Prenatal; Postpartum Vitamin D Dose (IU/Week)										
	N	0; 0	N	4200; 0	N	16800; 0	N	28000; 0	N	28000; 28000	p[a]
**Biochemical measures**											
Serum calcium (mmol/L), mean ± SD											
Maternal baseline	259	2.26 ± 0.09	260	2.25 ± 0.09	259	2.26 ± 0.10	260	2.26 ± 0.09	260	2.24 ± 0.09[b]	0.024
Maternal 30 week[c]	241	2.22 ± 0.10	239	2.23 ± 0.09	238	2.25 ± 0.09[d,e]	237	2.24 ± 0.09	242	2.24 ± 0.10	0.003
Maternal delivery[f]	206	2.26 ± 0.11	209	2.27 ± 0.12	209	2.31 ± 0.12[d,e]	199	2.28 ± 0.12	201	2.29 ± 0.12	<0.001
Maternal 3 months postpartum[g]	214	2.38 ± 0.09	220	2.39 ± 0.09	217	2.40 ± 0.10	216	2.40 ± 0.10	221	2.41 ± 0.10	0.045[h]
Maternal 6 months postpartum[i]	215	2.39 ± 0.09	222	2.39 ± 0.09	216	2.40 ± 0.09	215	2.39 ± 0.09	221	2.41 ± 0.10	0.173
Cord Blood	146	2.59 ± 0.19	154	2.59 ± 0.20	159	2.64 ± 0.15[d]	145	2.63 ± 0.20	149	2.64 ± 0.15	0.015
Infant 3 months of age[j]	128	2.55 ± 0.13	119	2.58 ± 0.13	122	2.62 ± 0.09[d]	123	2.62 ± 0.11[d]	127	2.61 ± 0.12[d]	<0.001
Infant 6 months of age[k]	157	2.60 ± 0.11	156	2.61 ± 0.11	148	2.61 ± 0.11	154	2.62 ± 0.11	151	2.62 ± 0.10	0.440
Urinary calcium:creatinine ratio (mmol/mmol), median (IQR)											
Maternal delivery[l]	198	0.20 (0.09, 0.43)	204	0.23 (0.12, 0.46)	191	0.28 (0.10, 0.48)	190	0.27 (0.12, 0.47)	197	0.28 (0.14, 0.53)[d]	0.034
Infant 6 months of age[m]	150	0.25 (0.12, 0.59)	151	0.34 (0.13, 0.64)	144	0.31 (0.11, 0.61)	154	0.25 (0.11, 0.66)	151	0.29 (0.12, 0.62)	0.856
iPTH (pmol/L), median (IQR)											
Maternal baseline	120	3.38 (2.37, 5.08)	119	4.02 (2.91, 5.41)	133	3.83 (2.51, 5.10)	114	4.05 (2.59, 5.36)	122	3.84 (2.71, 5.75)	0.393
Maternal delivery[n]	110	4.96 (3.27, 7.30)	107	3.49 (2.42, 4.80)[d]	123	2.91 (1.48, 4.44)[d]	100	2.90 (1.71, 4.55)[d]	111	2.40 (1.82, 3.97)[d,e]	<0.001
Maternal 6 months postpartum[o]	114	6.44 (4.24, 9.02)	117	7.43 (4.67, 9.97)	128	5.96 (3.96, 8.45)[e]	109	5.50 (3.98, 8.41)	119	4.45 (2.79, 6.96)[p]	<0.001
**Hypercalcemia[q]**											
Prenatal, maternal[r]											
Confirmed hypercalcemia[s], n (%)	259	0 (0.0)	260	0 (0.0)	259	0 (0.0)	260	0 (0.0)	260	0 (0.0)	-
Possible hypercalcemia[t], n (%)	259	1 (0.4)	260	1 (0.4)	259	4 (1.5)	260	3 (1.2)	260	2 (0.8)	0.558
Postpartum, maternal											
Confirmed hypercalcemia[s], n (%)	224	1 (0.4)	233	0 (0.0)	226	1 (0.4)	227	1 (0.4)	231	5 (2.2)	0.089
Possible hypercalcemia[t], n (%)	224	7 (3.1)	233	10 (4.3)	226	13 (5.8)	227	17 (7.5)	231	16 (6.9)	0.221
Infants											
Confirmed hypercalcemia[u], n (%)	199	0 (0.0)	195	2 (1.0)	188	1 (0.5)	185	1 (0.5)	186	2 (1.1)	0.623
Possible hypercalcemia[v], n (%)	199	12 (6.0)	195	5 (2.6)	188	11 (5.9)	185	9 (4.9)	186	11 (5.9)	0.482
**Hypercalciuria[q]**											
Prenatal, maternal[r]											
Confirmed hypercalciuria[w], n (%)	199	1 (0.5)	208	0 (0.0)	195	0 (0.0)	197	1 (0.5)	200	0 (0.0)	0.511
Possible hypercalciuria[x], n (%)	199	4 (2.0)	208	11 (5.3)	195	13 (6.7)	197	14 (7.1)	200	18 (9.0)[d]	0.048
Infants											
Confirmed hypercalciuria[y], n (%)	151	0 (0.0)	153	1 (0.7)	146	0 (0.0)	155	0 (0.0)	153	0 (0.0)	0.796
Possible hypercalciuria[z], n (%)	151	2 (1.3)	153	4 (2.6)	146	1 (0.7)	155	2 (1.3)	153	0 (0.0)	0.304

[a] P-value from ANOVA test for serum calcium measurements, Wilcoxon rank-sum test for urinary calcium:creatinine ratio and iPTH, and Chi-square or Fisher's tests for categorical variables.

[b] Post-hoc pairwise comparisons using t-tests or Wilcoxon rank-sum tests showed significant pairwise difference from group receiving 16800; 0, after adjusting for multiple comparisons using the Holm test.

[c] Measurements were excluded if they were collected <27 weeks gestation or >33 weeks gestation. $N_{0;\,0}$ = 3, $N_{4200;\,0}$ = 3, $N_{28000;\,0}$ =1, and $N_{28000;\,28000}$ = 2 measurements excluded.
[d] Post-hoc pairwise comparisons using t-tests, Wilcoxon rank-sum tests or Chi-square tests showed significant pairwise difference from placebo group, after adjusting for multiple comparisons using the Holm test.
[e] Post-hoc pairwise comparisons using t-tests or Wilcoxon rank-sum tests showed significant pairwise difference from group receiving 4200; 0, after adjusting for multiple comparisons using the Holm test.
[f] Measurements were excluded if they were collected <3 weeks before or after delivery. $N_{4200;\,0}$ = 1, $N_{16800;\,0}$ = 3, and $N_{28000;\,28000}$ = 1 measurements excluded.
[g] Measurements were excluded if they were collected >136 days (~4.5 months) postpartum. $N_{0;\,0}$ = 1, $N_{4200;\,0}$ = 2, $N_{16800;\,0}$ = 2, $N_{28000;\,0}$ = 1 and $N_{28000;\,28000}$ = 3 measurements excluded.
[h] Post-hoc pairwise comparisons using t-tests showed no significant pairwise differences between groups after adjusting for multiple comparisons using the Holm test.
[i] Measurements were excluded if they were collected >227 days (~7.5 months) postpartum. $N_{0;\,0}$ = 2, $N_{4200;\,0}$ = 1, $N_{16800;\,0}$ = 1, $N_{28000;\,0}$ = 1 and $N_{28000;\,28000}$ = 2 measurements excluded
[j] Measurements were excluded if they were collected at >136 days (~4.5 months) of age. $N_{4200;\,0}$ = 2, $N_{16800;\,0}$ = 2, and $N_{28000;\,28000}$ = 2 measurements excluded.
[k] Measurements were excluded if they were collected at >227 days (~7.5 months) of age. $N_{0;\,0}$ = 2, $N_{4200;\,0}$ = 1, $N_{16800;\,0}$ = 1, and $N_{28000;\,28000}$ = 2 measurements excluded.
[l] Measurements were excluded if they were collected <3 weeks before or after delivery. $N_{4200;\,0}$ = 1, $N_{16800;\,0}$ = 2, and $N_{28000;\,28000}$ = 1 measurements excluded.
[m] Measurements were excluded if they were collected at >227 days (~7.5 months) of age. $N_{0;\,0}$ = 1, $N_{4200;\,0}$ = 1, $N_{16800;\,0}$ = 1, and $N_{28000;\,28000}$ = 2 measurements were excluded.
[n] Measurements were excluded if they were collected <3 weeks before or after delivery. $N_{4200;\,0}$ = 1 and $N_{16800;\,0}$ = 2 measurements excluded
[o] Measurements were excluded if they were collected >227 days (~7.5 months) postpartum. $N_{0;\,0}$ = 2, $N_{4200;\,0}$ = 1, $N_{16800;\,0}$ = 1 and $N_{28000;\,28000}$ = 1 measurements excluded.
[p] Post-hoc pairwise comparisons using Wilcoxon rank-sum tests showed significant pairwise difference from groups receiving 0; 0, 4200; 0, 16800; 0 and 28000; 0, after adjusting for multiple comparisons using the Holm test.
[q] Includes measurements collected for routine monitoring, and any measurements collected during an adverse event or for clinical follow up. Includes measurements collected up to 1 year after delivery in the postpartum period.
[r] Includes measurements taken at delivery.
[s] Serum calcium >2.6 mmol/L in two repeat samples, or >2.8 mmol/L in one sample when no repeat sample was available.
[t] Serum calcium >2.6 mmol/L in one sample and ≤2.6 mmol/L in a repeat sample; or serum calcium >2.6 mmol/L in one sample when no repeat sample was available.
[u] Serum calcium >2.8 mmol/L in two repeat samples.
[v] Serum calcium >2.8 mmol/L in one sample and ≤2.8 mmol/L in a repeat sample; or serum calcium >2.8 mmol/L in one sample when no repeat sample was available.
[w] Urinary calcium:creatinine ratio >1 mmol/mmol in two repeat samples.
[x] Urinary calcium:creatinine ratio >1 mmol/mmol in one sample and ≤1 mmol/mmol in a repeat sample; or calcium:creatinine ratio >1 mmol/mmol in one sample when no repeat sample was available.
[y] Urinary calcium:creatinine >2.42 mmol/mmol in two repeat samples.
[z] Urinary calcium:creatinine >2.42 mmol/mmol in one sample and ≤2.42 mmol/mmol in a second repeat sample; or calcium:creatinine >2.42 mmol/mmol in one sample when no repeat sample was available.

Table S26. Serum calcium adjusted for differences at baseline or in cord blood for women and infants, respectively[a]

Serum Calcium	N per group in unadjusted model	Unadjusted mean/mean difference (95 % CI)[b]	N per group in adjusted model	Adjusted mean/mean difference (95 % CI)[b]
**Women[c]**				
30 weeks				
0; 0	241	2.223 (2.211, 2.235)	241	2.221 (2.210, 2.232)
4200; 0	239	0.006 (-0.011, 0.023)	239	0.008 (-0.008, 0.024)
16800; 0	238	**0.032 (0.015, 0.048)**	238	**0.029 (0.013, 0.044)**
28000; 0	237	**0.019 (0.002, 0.035)**	237	**0.019 (0.004, 0.035)**
28000; 28000	242	0.015 (-0.020, 0.032)	242	**0.023 (0.008, 0.039)**
		-0.003 (-0.020, 0.013)[d]		0.004 (-0.012, 0.020)[d]
Delivery				
0; 0	206	2.257 (2.241, 2.273)	206	2.255 (2.240, 2.271)
4200; 0	209	0.014 (-0.008, 0.037)	209	0.016 (-0.006, 0.038)
16800; 0	219	**0.050 (0.027, 0.072)**	209	**0.046 (0.024, 0.068)**
28000; 0	199	0.022 (-0.001, 0.044)	199	0.022 (-0.000, 0.044)
28000; 28000	201	**0.030 (0.007, 0.053)**	201	**0.036 (0.014, 0.058)**
		0.008 (-0.015, 0.031)[d]		0.014 (-0.008, 0.036)[d]
3 months postpartum				
0; 0	214	2.381 (2.368, 2.394)	214	2.380 (2.368, 2.393)
4200; 0	220	0.013 (-0.005, 0.031)	220	0.013 (-0.004, 0.031)
16800; 0	217	**0.022 (0.004, 0.041)**	217	**0.019 (0.001, 0.037)**
28000; 0	216	**0.022 (0.003, 0.040)**	216	**0.021 (0.003, 0.039)**
28000; 28000	221	**0.025 (0.007, 0.043)**	221	**0.029 (0.011, 0.046)**
		0.004 (-0.015, 0.022)[d]		0.008 (-0.010, 0.025)[d]
6 months postpartum				
0; 0	215	2.385 (2.373, 2.398)	215	2.386 (2.373, 2.397)
4200; 0	222	0.007 (-0.011, 0.024)	222	0.007 (-0.010, 0.024)
16800; 0	216	0.011 (-0.007, 0.028)	216	0.008 (-0.009, 0.024)
28000; 0	215	0.001 (-0.016, 0.019)	215	0.001 (-0.016, 0.018)
28000; 28000	221	**0.020 (0.002, 0.037)**	221	**0.025 (0.008, 0.041)**
		**0.018 (0.001, 0.036)[d]**		**0.023 (0.007, 0.040)[d]**
**Infants[e]**				
3 months of age				
0; 0	128	2.554 (2.533, 2.574)	83	2.557 (2.531, 2.582)
4200; 0	119	**0.030 (0.000, 0.059)**	76	0.033 (-0.003, 0.069)
16800; 0	122	**0.062 (0.033, 0.092)**	87	**0.058 (0.023, 0.093)**
28000; 0	123	**0.068 (0.039, 0.097)**	79	**0.057 (0.022, 0.093)**
28000; 28000	127	**0.054 (0.025, 0.083)**	84	**0.054 (0.019, 0.090)**
		-0.014 (-0.043, 0.015)[d]		-0.003 (-0.038, 0.032)[d]

**Serum Calcium**	**N per group in unadjusted model**	**Unadjusted mean/mean difference (95 % CI)[b]**	**N per group in adjusted model**	**Adjusted mean/mean difference (95 % CI)[b]**
6 months of age				
0; 0	157	2.600 (2.583, 2.618)	95	2.600 (2.577, 2.622)
4200; 0	156	0.006 (-0.019, 0.003)	97	0.015 (-0.018, 0.046)
16800; 0	148	0.011 (-0.013, 0.036)	104	0.014 (-0.016, 0.045)
28000; 0	154	0.015 (-0.010, 0.039)	94	0.016 (-0.016, 0.048)
28000; 28000	151	0.022 (-0.002, 0.047)	103	0.025 (-0.007, 0.056)
		0.008 (-0.017, 0.032)[d]		0.009 (-0.023, 0.040)[d]

[a] Post-hoc analysis undertaken due to differences in maternal calcium at baseline and in cord blood.

[b] Mean is presented for placebo group; mean difference versus placebo presented for all other groups, unless otherwise indicated. Coefficients in bold reflect values that were significantly different (p<0.05) from reference group.

[c] Adjusted for serum calcium at baseline.

[d] Mean difference (95% CI) versus group receiving a prenatal; postpartum dose of 28000; 0 IU/week.

[e] Adjusted for cord blood serum calcium.

Table S27. Clinical encounters, hospitalizations and other clinical adverse events among women and infants, by supplementation group

	Prenatal; Postpartum Vitamin D Dose (IU/Week)					
	**0; 0**<br>**N = 259**	**4200; 0**<br>**N = 260**	**16800; 0**<br>**N = 259**	**28000; 0**<br>**N = 260**	**28000; 28000**<br>**N = 260**	**p[a]**
**Women[b,c]**						
Any clinical encounter[d]						
Women with at least one encounter, n (%)	64 (24.7)	68 (26.2)	67 (25.9)	84 (32.3)	86 (33.1)	0.094
Encounter rate (95% CI), per 1,000 completed weekly visits	8.5 (6.4, 10.5)	8.9 (6.8, 10.9)	8.0 (6.2, 9.9)	11.4 (9.1, 13.8)	10.8 (8.7, 13.0)	0.215
Total number of encounters, n	80	85	77	108	105	
Urolithiasis/nephrolithiasis						
Women with at least one event, n (%)	0 (0.0)	0 (0.0)	0 (0.0)	0 (0.0)	0 (0.0)	-
Encounter rate (95% CI), per 1,000 completed weekly visits	0	0	0	0	0	-
Total number of events, n	0	0	0	0	0	
Hospitalization						
Women with at least one event, n (%)	23 (8.9)	20 (7.7)	26 (10.0)	29 (11.2)	21 (8.1)	0.637
Hospitalization rate (95% CI), per 1,000 completed weekly visits	2.8 (1.6, 4.1)	2.2 (1.2, 3.1)	2.8 (1.7, 3.9)	3.8 (2.3, 5.3)	2.5 (1.3, 3.6)	0.291
Total number of events, n	26	21	27	34	23	
At least one referral to hospital or specialist up to one month postpartum, n (%)	23 (8.9)	20 (7.7)	22 (8.5)	25 (9.6)	20 (7.7)	0.924
Gestational hypertension[e], n (%)	9 (3.5)	13 (5.0)	14 (5.4)	14 (5.4)	18 (6.9)	0.531
Death[f], n (%)	1 (0.4)	1 (0.4)	0 (0.0)	0 (0.0)	0 (0.0)	0.759
**Infants[g]**						
Any clinical encounter[d]						
During neonatal (≤ 28 days of age) period						
Infants with at least one encounter, n (%)	82 (33.2)	83 (32.7)	81 (32.1)	71 (28.2)	75 (30.1)	0.731
Encounter rate (95% CI), per 1,000 completed weekly visits	101.0 (80.1, 121.9)	102.5 (81.8, 123.2)	99.8 (79.0, 120.5)	91.1 (71.3, 111.0)	90.9 (71.4, 110.5)	0.883
Total number of encounters, n	90	94	89	81	83	
During 29 days to <6 months of age						
Infants with at least one encounter, n (%)	188 (77.4)	211 (83.7)	201 (81.4)	195 (78.0)	197 (79.8)	0.382
Encounter rate (95% CI), per 1,000 completed weekly visits	99.2 (88.8, 109.6)	110.5 (99.9, 121.1)	109.6 (99.1, 120.1)	105.8 (95.1, 116.4)	99.5 (89.6, 109.4)	0.574
Total number of encounters, n	461	508	509	485	462	
During 6 to 12 months of age						
Infants with at least one encounter, n (%)	118 (48.8)	130 (51.8)	122 (49.4)	128 (51.6)	125 (50.6)	0.951
Total number of encounters, n	198	217	177	195	187	
Hospitalization						
During neonatal (≤ 28 days of age) period						

	Prenatal; Postpartum Vitamin D Dose (IU/Week)					p[a]
	0; 0<br>N = 259	4200; 0<br>N = 260	16800; 0<br>N = 259	28000; 0<br>N = 260	28000; 28000<br>N = 260	
Infants with at least one event, n (%)	40 (16.2)	38 (15.0)	41 (16.3)	35 (13.9)	40 (16.1)	0.936
Hospitalization rate (95% CI), per 1,000 completed weekly visits	46.0 (31.9, 60.1)	41.4 (28.3, 54.6)	47.1 (32.8, 61.3)	39.4 (26.3, 52.4)	46.0 (32.1, 59.9)	0.921
Total number of events, n	41	38	42	35	42	
During 29 days to <6 months of age						
Infants with at least one event, n (%)	25 (10.3)	22 (8.7)	31 (12.6)	31 (12.4)	20 (8.1)	0.348
Hospitalization rate (95% CI), per 1,000 completed weekly visits	5.8 (3.6, 8.0)	5.9 (3.3, 8.5)	7.3 (4.8, 9.9)	7.2 (4.7, 9.7)	4.5 (2.6, 6.5)	0.133
Total number of events, n	27	27	34	33	21	
During 6 to 12 months of age						
Infants with at least one event, n (%)	18 (7.4)	29 (11.6)	16 (6.5)	20 (8.1)	20 (8.1)	0.307
Total number of events, n	20	34	17	21	21	
X-ray confirmed rickets[h], n (%)	3 (1.8)	1 (0.6)	0 (0.0)	0 (0.0)	0 (0.0)	0.135
Neurological disability, n (%)	1 (0.4)	1 (0.4)	1 (0.4)	0 (0.0)	0 (0.0)	0.805
Death, n (%)						
Ever	6 (2.4)	3 (1.2)	5 (2.0)	5 (2.0)	2 (0.8)	0.603
During neonatal (≤ 28 days of age) period	4 (1.6)	2 (0.8)	5 (2.0)	2 (0.8)	2 (0.8)	0.653
During 29 days to <6 months of age	1 (0.4)	1 (0.4)	0 (0.0)	2 (0.8)	0 (0.0)	0.592
During 6 to 12 months of age	1 (0.4)	0 (0.0)	0 (0.0)	1 (0.4)	0 (0.0)	0.436

[a] P-value from Chi-square or Fischer's tests for categorical variables and zero-inflated negative binomial regression models for rates.
[b] Only clinical encounters and hospitalizations during the intervention phase (prenatal period and up to 6 months postpartum) are reported.
[c] Proportions are presented using number of mother-infant pairs assigned to the treatment group as the denominator.
[d] Includes outpatient and emergency room visits and inpatient admissions (i.e., hospitalizations).
[e] Women were classified as having gestational hypertension if at least one of the following two criteria were met: (1) Elevated blood pressure ever detected in routine prenatal monitoring: blood pressure was measured in duplicate (and measured a third time if the difference in paired systolic or diastolic measurements was ≥10 mmHg) at routine prenatal visits (see Table S2 for schedule). Hypertension was defined as ≥2 systolic blood pressure readings ≥140 mmHg, and/or ≥2 diastolic blood pressure readings ≥90 mmHg at the same visit; if only one set of measurements was available, a single systolic reading ≥140 mmHg or a single diastolic reading ≥90 mmHg was used to define gestational hypertension; (2) Prenatal clinical encounter or serious adverse event (hospitalization or death) with documentation of hypertension and/or management of hypertension.
[f] Both deaths were considered to be maternal deaths (death while pregnant or within 42 days termination of pregnancy, irrespective of the duration and site of the pregnancy, from any cause related to or aggregated by the pregnancy or its management but not from accidental or incidental causes). For the participant in the placebo group, cause of death was cardiac respiratory arrest at 35 weeks gestation. For the participant in the 4200;0 group, cause of death at 34 weeks gestation was unconfirmed, but with clinical signs of sepsis/meningitis.
[g] Proportions are presented using number of live births in the treatment group as the denominator, unless otherwise indicated. $N_{0;\,0}$ = 247, $N_{4200;\,0}$ = 254, $N_{16800;\,0}$ = 252, $N_{28000;\,0}$ = 252, $N_{28000;\,28000}$ = 249
[h] Proportions are presented using number of infants screened for rickets as the denominator. $N_{0;\,0}$ = 165, $N_{4200;\,0}$ = 161, $N_{16800;\,0}$ = 154, $N_{28000;\,0}$ = 155, $N_{28000;\,28000}$ = 152

Table S28. Additional delivery characteristics and pregnancy outcomes, by supplementation group

	Prenatal; Postpartum Vitamin D Dose (IU/Week)					
	0; 0	4200; 0	16800; 0	28000; 0	28000; 28000	p[b]
**Characteristic/Outcome**[a]	**N = 259**	**N = 260**	**N = 259**	**N = 260**	**N = 260**	
Delivery outcome, n (%)						0.41
Live birth	247 (95.4)	254 (97.7)	252 (97.3)	252 (96.9)	249 (95.8)	
Intrauterine death/stillbirth	8 (3.1)	4 (1.5)	2 (0.8)	7 (2.7)	8 (3.1)	
Maternal death prior to delivery	1 (0.4)	1 (0.4)	0 (0.0)	0 (0.0)	0 (0.0)	
Lost to follow-up during pregnancy	3 (1.2)	1 (0.4)	5 (1.9)	1 (0.4)	3 (1.2)	
Gestational age at birth (weeks)						
Early preterm (<32 weeks), n (%)	0 (0.0)	0 (0.0)	1 (0.4)	1 (0.4)	1 (0.4)	0.89
Moderate to late preterm (≥32 weeks to <37 weeks), n (%)	24 (9.7)	21 (8.3)	30 (11.9)	25 (9.9)	21 (8.4)	
Term (≥37 weeks to <42 weeks), n (%)	219 (88.7)	226 (89.0)	217 (86.1)	222 (88.1)	224 (90.0)	
Postterm (≥42 weeks), n (%)	4 (1.6)	7 (2.8)	4 (1.6)	4 (1.6)	3 (1.2)	
Early preterm (<34 weeks)[c], n (%)	3 (1.2)	0 (0.0)	3 (1.2)	2 (0.8)	3 (1.2)	0.46
Location of delivery, n (%)						0.91
Hospital or clinic	211 (85.4)	216 (85.0)	216 (85.7)	212 (84.1)	207 (83.1)	
Home	35 (14.2)	38 (15.0)	35 (13.9)	40 (15.9)	42 (16.9)	
Other	1 (0.4)	0 (0.0)	1 (0.4)	0 (0.0)	0 (0.0)	
Congenital anomaly, n (%)	16 (6.5)	6 (2.4)	5 (2.0)	8 (3.2)	7 (2.8)	0.04[d]
Month of birth, n (%)						0.16
March-May	33 (13.4)	39 (15.4)	42 (16.7)	26 (10.3)	43 (17.3)	
June-August	73 (29.6)	66 (26.0)	69 (27.4)	80 (31.7)	72 (28.9)	
September-November	87 (35.2)	76 (29.9)	91 (36.1)	85 (33.7)	68 (27.3)	
December-February	54 (21.9)	73 (28.7)	50 (19.8)	61 (24.2)	66 (26.5)	
Term low birth weight[e, f], n (%)[g]	30 (20.1)	46 (28.8)	35 (22.4)	43 (29.1)	32 (20.3)	0.16
Length-for-age z-score <-2[e], n (%)[h]	25 (15.2)	24 (14.2)	27 (16.3)	21 (13.1)	14 (8.5)	0.27
Head circumference-for-age z-score <-2[e], n (%)[i]	14 (8.4)	16 (9.5)	10 (5.9)	19 (11.9)	11 (6.6)	0.30
Weight-for-age z-score <-2[e], n (%)[j]	24 (14.5)	35 (20.5)	28 (16.7)	35 (21.7)	20 (11.8)	0.09
Placenta weight (g), mean (SD)[k]	370 ± 66	362 ± 72	376 ± 69	369 ± 69	375 ± 70	0.38

[a] Except for delivery outcome and maternal serum 25(OH)D concentration at/near delivery, all characteristics and outcomes presented are among live births only.

[b] P-values from ANOVA or Kruskal-Wallis tests for continuous variables, and Chi-square or Fischer's tests for categorical variables.

[c] Definition of early preterm specified in the protocol.

[d] Post-hoc pairwise comparisons using Chi-square tests showed no significant differences between groups after adjusting for multiple comparisons using the Holm test.

[e] Limited to measurements obtained within 48 hours of birth.

[f] Weight <2500 g and born at ≥37 weeks gestational age.

[g] $N_{0;\,0}$ = 149, $N_{4200;\,0}$ = 160, $N_{16800;\,0}$ = 156, $N_{28000;\,0}$ = 148, $N_{28000;\,28000}$ = 158

[h] $N_{0;\,0}$ = 164, $N_{4200;\,0}$ = 169, $N_{16800;\,0}$ = 166, $N_{28000;\,0}$ = 160, $N_{28000;\,28000}$ = 165

[i] $N_{0;\,0}$ = 167, $N_{4200;\,0}$ = 168, $N_{16800;\,0}$ = 169, $N_{28000;\,0}$ = 159, $N_{28000;\,28000}$ = 167

[j] $N_{0;\,0}$ = 166, $N_{4200;\,0}$ = 171, $N_{16800;\,0}$ = 168, $N_{28000;\,0}$ = 161, $N_{28000;\,28000}$ = 169

[k] $N_{0;\,0}$ = 168, $N_{4200;\,0}$ = 175, $N_{16800;\,0}$ = 175, $N_{28000;\,0}$ =163, $N_{28000;\,28000}$ = 172

Table S29. Primary diagnoses at clinical encounters and primary discharge diagnoses for hospitalizations among women up to 6 months postpartum[a]

Prenatal; Postpartum Vitamin D Dose (IU/Week)	Participants who ever received the diagnosis, n (%)					Clinical encounters or hospitalizations at which the diagnosis was assigned, n[b]				
	0; 0	4200; 0	16800; 0	28000; 0	28000; 28000	0; 0	4200; 0	16800; 0	28000; 0	28000; 28000
**Primary diagnosis[c]**	**N=259**	**N=260**	**N=259**	**N=260**	**N=260**					
Acute respiratory infection	10 (3.8)	8 (3.1)	8 (3.1)	12 (4.6)	16 (6.2)	10	9	8	13	16
Diarrhea	2 (0.8)	7 (2.7)	9 (3.5)	7 (2.7)	9 (3.5)	2	7	9	7	10
Abdominal pain, not otherwise specified	5 (1.9)	3 (1.2)	9 (3.5)	6 (2.3)	3 (1.2)	5	3	9	6	3
Other gastrointestinal illness	1 (0.4)	7 (2.7)	3 (1.2)	9 (3.5)	7 (2.7)	1	7	3	10	7
Hypertensive disease of pregnancy	4 (1.5)	1 (0.4)	4 (1.5)	3 (1.2)	7 (2.7)	4	1	4	3	8
Postpartum wound infection	5 (1.9)	6 (2.3)	3 (1.2)	3 (1.2)	1 (0.4)	5	6	3	3	1
Back/limb pain	6 (2.3)	5 (1.9)	4 (1.5)	7 (2.7)	4 (1.5)	6	5	4	7	5
Vaginal bleeding	4 (1.5)	5 (1.9)	1 (0.4)	7 (2.7)	2 (0.8)	5	5	1	8	2
Anemia	3 (1.2)	4 (1.5)	1 (0.4)	2 (0.8)	4 (1.5)	5	4	1	3	4
Other Infection	7 (2.7)	3 (1.2)	5 (1.9)	5 (1.9)	4 (1.5)	7	3	5	5	4
Gestational diabetes	3 (1.2)	2 (0.8)	0 (0.0)	1 (0.4)	2 (0.8)	3	2	0	2	2
Trauma	1 (0.4)	0 (0.0)	1 (0.4)	1 (0.4)	2 (0.8)	1	0	1	1	2
Other	8 (3.1)	10 (3.8)	10 (3.8)	11 (4.2)	14 (5.4)	9	10	12	12	15
Organ failure and/or death	7 (2.7)	3 (1.2)	2 (0.8)	7 (2.7)	6 (2.3)	7	3	2	7	6
**Primary discharge diagnosis[d]**										
Acute respiratory infection	0 (0)	0 (0)	0 (0)	0 (0)	0 (0)	0	0	0	0	0
Diarrhea	2 (0.8)	6 (2.3)	7 (2.7)	3 (1.2)	9 (3.5)	2	6	7	3	9
Abdominal pain, not otherwise specified	3 (1.2)	2 (0.8)	3 (1.2)	2 (0.8)	0 (0.0)	3	2	3	2	0
Other gastrointestinal illness	0 (0.0)	3 (1.2)	0 (0.0)	5 (1.9)	0 (0.0)	0	3	0	6	0
Hypertensive disease of pregnancy	0 (0.0)	0 (0.0)	3 (1.2)	2 (0.8)	1 (0.4)	0	0	3	2	2
Postpartum wound infection	3 (1.2)	6 (2.3)	3 (1.2)	3 (1.2)	1 (0.4)	3	6	3	3	1
Back/limb pain	0 (0)	0 (0)	0 (0)	0 (0)	0 (0)	0	0	0	0	0
Vaginal bleeding	1 (0.4)	0 (0.0)	0 (0.0)	5 (1.9)	0 (0.0)	2	0	0	6	0
Anemia	0 (0.0)	0 (0.0)	0 (0.0)	1 (0.4)	0 (0.0)	0	0	0	1	0
Other Infection	2 (0.8)	1 (0.4)	2 (0.8)	2 (0.8)	1 (0.4)	2	1	2	2	1
Gestational diabetes	0 (0)	0 (0)	0 (0)	0 (0)	0 (0)	0	0	0	0	0
Trauma	1 (0.4)	0 (0.0)	1 (0.4)	1 (0.4)	1 (0.4)	1	0	1	1	1
Other	4 (1.5)	1 (0.4)	6 (2.3)	4 (1.5)	3 (1.2)	4	1	7	4	3
Organ failure and/or death	9 (3.5)	2 (0.8)	1 (0.4)	4 (1.5)	6 (2.3)	9	2	1	4	6

[a] Post-hoc categorization of encounters and hospitalizations was based on physician-assigned diagnoses assigned at the time of the event (or as soon as possible after the event) without standardized definitions or criteria.

[b] A participant may contribute multiple events per primary diagnosis or primary discharge diagnosis.

[c] Includes outpatient and emergency room visits and inpatient admissions (i.e., hospitalizations).

[d] Hospitalizations only.

Table S30. Primary diagnoses at clinical encounters and primary discharge diagnoses for hospitalizations among infants up to 6 months of age[a]

Prenatal; Postpartum Vitamin D Dose (IU/Week)	Participants who ever received the diagnosis, n (%)					Clinical encounters or hospitalizations at which the diagnosis was assigned, n[b]				
	0; 0 N=247	4200; 0 N=254	16800; 0 N=252	28000; 0 N=252	28000; 28000 N=249	0; 0	4200; 0	16800; 0	28000; 0	28000; 28000
**Primary diagnosis[c]**										
Congenital anomaly/disorder	7 (2.8)	1 (0.4)	5 (2.0)	3 (1.2)	2 (0.8)	7	1	6	4	2
Neonatal respiratory distress	6 (2.4)	11 (4.3)	7 (2.8)	7 (2.8)	6 (2.4)	6	11	7	7	6
Perinatal asphyxia	8 (3.2)	3 (1.2)	4 (1.6)	5 (2.0)	3 (1.2)	8	3	4	5	3
Jaundice	13 (5.3)	10 (3.9)	12 (4.8)	8 (3.2)	13 (5.2)	13	10	12	8	13
Microcephaly	0 (0)	0 (0)	0 (0)	0 (0)	0 (0)	0	0	0	0	0
Prematurity	1 (0.4)	3 (1.2)	2 (0.8)	1 (0.4)	1 (0.4)	1	3	2	1	1
Acute respiratory infection	159 (64.4)	172 (67.7)	165 (65.5)	166 (65.9)	161 (64.7)	337	389	400	376	332
Diarrhea	23 (9.3)	30 (11.8)	20 (7.9)	23 (9.1)	24 (9.6)	26	36	23	29	28
Sepsis/meningitis	7 (2.8)	2 (0.8)	7 (2.8)	10 (4.0)	11 (4.4)	7	2	7	10	11
Other infection	12 (4.9)	15 (5.9)	10 (4.0)	10 (4.0)	13 (5.2)	12	16	11	11	15
Nutritional/feeding concern	5 (2.0)	3 (1.2)	3 (1.2)	4 (1.6)	4 (1.6)	5	3	3	4	4
Seizure	0 (0.0)	2 (0.8)	1 (0.4)	0 (0.0)	0 (0.0)	0	2	1	0	0
Suspected rickets, vitamin D deficiency[d]	0 (0.0)	0 (0.0)	0 (0.0)	2 (0.8)	0 (0.0)	0	0	0	2	0
Skin rash	6 (2.4)	7 (2.8)	5 (2.0)	6 (2.4)	6 (2.4)	7	7	5	6	6
Anemia	91 (36.8)	100 (39.4)	95 (37.7)	84 (33.3)	98 (39.4)	91	100	95	85	98
Other	10 (4.0)	9 (3.5)	10 (4.0)	5 (2.0)	6 (2.4)	10	9	10	5	6
Organ failure and/or death	1 (0.4)	0 (0.0)	0 (0.0)	0 (0.0)	1 (0.4)	1	0	0	0	1
**Primary discharge diagnosis[e]**										
Congenital anomaly/disorder	2 (0.8)	0 (0.0)	1 (0.4)	2 (0.8)	1 (0.4)	2	0	1	2	1
Neonatal respiratory distress	6 (2.4)	10 (3.9)	5 (2.0)	9 (3.6)	7 (2.8)	6	10	5	9	7
Perinatal asphyxia	7 (2.8)	4 (1.6)	7 (2.8)	3 (1.2)	2 (0.8)	7	4	7	3	2
Jaundice	9 (3.6)	5 (2.0)	10 (4.0)	6 (2.4)	12 (4.8)	9	5	10	6	12
Microcephaly	0 (0)	0 (0)	0 (0)	0 (0)	0 (0)	0	0	0	0	0
Prematurity	1 (0.4)	2 (0.8)	0 (0.0)	2 (0.8)	2 (0.8)	1	2	0	2	2
Acute respiratory infection	15 (6.1)	14 (5.5)	25 (9.9)	18 (7.1)	9 (3.6)	16	14	27	19	9
Diarrhea	11 (4.5)	11 (4.3)	10 (4.0)	12 (4.8)	10 (4.0)	11	14	10	14	11
Sepsis/meningitis	5 (2.0)	5 (2.0)	5 (2.0)	6 (2.4)	13 (5.2)	5	5	5	6	13
Other infection	3 (1.2)	5 (2.0)	2 (0.8)	1 (0.4)	4 (1.6)	3	5	2	1	4
Nutritional/feeding concern	5 (2.0)	3 (1.2)	3 (1.2)	2 (0.8)	2 (0.8)	5	3	3	2	2
Seizure	0 (0.0)	2 (0.8)	0 (0.0)	0 (0.0)	0 (0.0)	0	2	0	0	0
Suspected rickets, vitamin D deficiency[d]	0 (0)	0 (0)	0 (0)	0 (0)	0 (0)	0	0	0	0	0
Skin rash	0 (0)	0 (0)	0 (0)	0 (0)	0 (0)	0	0	0	0	0
Anemia	0 (0.0)	0 (0.0)	0 (0.0)	1 (0.4)	0 (0.0)	0	0	0	1	0
Other	3 (1.2)	1 (0.4)	4 (1.6)	2 (0.8)	0 (0.0)	3	1	4	2	0

Prenatal; Postpartum Vitamin D Dose (IU/Week)	Participants who ever received the diagnosis, n (%)					Clinical encounters or hospitalizations at which the diagnosis was assigned, n[b]				
	0; 0<br>N=247	4200; 0<br>N=254	16800; 0<br>N=252	28000; 0<br>N=252	28000; 28000<br>N=249	0; 0	4200; 0	16800; 0	28000; 0	28000; 28000
Organ failure and/or death	0 (0.0)	0 (0.0)	2 (0.8)	1 (0.4)	0 (0.0)	0	0	2	1	0

[a] Post-hoc categorization of encounters and hospitalizations was based on physician-assigned diagnoses assigned at the time of the event (or as soon as possible after the event) without standardized definitions or criteria.

[b] A participant may contribute multiple events per primary diagnosis or primary discharge diagnosis.

[c] Includes outpatient and emergency room visits and inpatient admissions (i.e., hospitalizations).

[d] Encounters/hospitalizations for suspected rickets does not align with number of x-ray confirmed rickets cases reported in Table S27 because of different diagnostic criteria.

[e] Hospitalizations only.

Table S31. Primary diagnoses at clinical encounters and primary discharge diagnoses for hospitalizations among infants from 6-12 months of age[a]

Prenatal; Postpartum Vitamin D Dose (IU/Week)	Participants who ever received the diagnosis, n (%)					Clinical encounters or hospitalizations at which the diagnosis was assigned, n[b]				
	0; 0 N=242	4200; 0 N=251	16800; 0 N=247	28000; 0 N=248	28000; 28000 N=247	0; 0	4200; 0	16800; 0	28000; 0	28000; 28000
**Primary diagnosis[c]**										
Congenital anomaly/disorder	7 (2.9)	4 (1.6)	3 (1.2)	2 (0.8)	1 (0.4)	7	4	2	2	1
Neonatal respiratory distress	0 (0)	0 (0)	0 (0)	0 (0)	0 (0)	0	0	0	0	0
Perinatal asphyxia	0 (0)	0 (0)	0 (0)	0 (0)	0 (0)	0	0	0	0	0
Jaundice	0 (0)	0 (0)	0 (0)	0 (0)	0 (0)	0	0	0	0	0
Microcephaly	3 (1.2)	1 (0.4)	0 (0.0)	0 (0.0)	1 (0.4)	3	1	0	0	1
Prematurity	0 (0)	0 (0)	0 (0)	0 (0)	0 (0)	0	0	0	0	0
Acute respiratory infection	34 (14.0)	35 (13.9)	34 (13.8)	35 (14.1)	30 (12.1)	39	43	37	40	34
Diarrhea	7 (2.9)	14 (5.6)	9 (3.6)	14 (5.6)	16 (6.5)	7	15	10	14	17
Sepsis/meningitis	1 (0.4)	0 (0.0)	0 (0.0)	1 (0.4)	0 (0.0)	1	0	0	1	0
Other infection	4 (1.7)	2 (0.8)	2 (0.8)	4 (1.6)	3 (1.2)	4	2	2	4	3
Nutritional/feeding concern	7 (2.9)	3 (1.2)	2 (0.8)	3 (1.2)	2 (0.8)	8	3	4	5	2
Seizure	1 (0.4)	0 (0.0)	0 (0.0)	0 (0.0)	1 (0.4)	1	0	0	0	1
Suspected rickets, vitamin D deficiency[d]	3 (1.2)	0 (0.0)	2 (0.8)	0 (0.0)	2 (0.8)	3	0	2	0	2
Skin rash	1 (0.4)	0 (0.0)	2 (0.8)	2 (0.8)	0 (0.0)	1	0	2	2	0
Anemia	82 (33.9)	91 (36.3)	79 (32.0)	84 (33.9)	85 (34.4)	118	137	105	117	118
Other	1 (0.4)	2 (0.8)	5 (2.0)	3 (1.2)	2 (0.8)	1	2	5	3	2
Organ failure and/or death	1 (0.4)	0 (0.0)	0 (0.0)	0 (0.0)	0 (0.0)	1	0	0	0	0
**Primary discharge diagnosis[e]**										
Congenital anomaly/disorder	2 (0.8)	0 (0.0)	0 (0.0)	0 (0.0)	0 (0.0)	2	0	0	0	0
Neonatal respiratory distress	0 (0)	0 (0)	0 (0)	0 (0)	0 (0)	0	0	0	0	0
Perinatal asphyxia	0 (0)	0 (0)	0 (0)	0 (0)	0 (0)	0	0	0	0	0
Jaundice	0 (0)	0 (0)	0 (0)	0 (0)	0 (0)	0	0	0	0	0
Microcephaly	0 (0)	0 (0)	0 (0)	0 (0)	0 (0)	0	0	0	0	0
Prematurity	0 (0)	0 (0)	0 (0)	0 (0)	0 (0)	0	0	0	0	0
Acute respiratory infection	7 (2.9)	16 (6.4)	5 (2.0)	7 (2.8)	4 (1.6)	7	17	5	7	4
Diarrhea	5 (2.1)	14 (5.6)	8 (3.2)	10 (4.0)	13 (5.3)	5	15	9	10	14
Sepsis/meningitis	1 (0.4)	0 (0.0)	0 (0.0)	1 (0.4)	0 (0.0)	1	0	0	1	0
Other infection	0 (0.0)	1 (0.4)	0 (0.0)	3 (1.2)	1 (0.4)	0	1	0	3	1
Nutritional/feeding concern	2 (0.8)	1 (0.4)	1 (0.4)	0 (0.0)	1 (0.4)	2	1	1	0	1
Seizure	1 (0.4)	0 (0.0)	0 (0.0)	0 (0.0)	1 (0.4)	1	0	0	0	1
Suspected rickets, vitamin D deficiency[d]	0 (0)	0 (0)	0 (0)	0 (0)	0 (0)	0	0	0	0	0
Skin rash	0 (0)	0 (0)	0 (0)	0 (0)	0 (0)	0	0	0	0	0
Anemia	0 (0)	0 (0)	0 (0)	0 (0)	0 (0)	0	0	0	0	0
Other	1 (0.4)	0 (0.0)	1 (0.4)	0 (0.0)	0 (0.0)	1	0	1	0	0

Prenatal; Postpartum Vitamin D Dose (IU/Week)	Participants who ever received the diagnosis, n (%)					Clinical encounters or hospitalizations at which the diagnosis was assigned, n[b]				
	0; 0<br>N=242	4200; 0<br>N=251	16800; 0<br>N=247	28000; 0<br>N=248	28000; 28000<br>N=247	0; 0	4200; 0	16800; 0	28000; 0	28000; 28000
Organ failure and/or death	1 (0.4)	0 (0.0)	0 (0.0)	0 (0.0)	0 (0.0)	1	0	0	0	0

[a] Post-hoc categorization of encounters and hospitalizations was based on physician-assigned diagnoses assigned at the time of the event (or as soon as possible after the event) without standardized definitions or criteria.

[b] A participant may contribute multiple events per primary diagnosis or primary discharge diagnosis.

[c] Includes outpatient and emergency room visits and inpatient admissions (i.e., hospitalizations).

[d] Encounters/hospitalizations for suspected rickets does not align with number of x-ray confirmed rickets cases reported in Table S27 because of different diagnostic criteria.

[e] Hospitalizations only.

Table S32. Frequencies of reported symptoms among women based on weekly clinical monitoring during the prenatal and 6 month postpartum periods[a]

Prenatal; Postpartum Vitamin D Dose (IU/Week)	Number of women ever having reported the symptom, n (%)						Number of times the symptom was reported, n (%)[b]					
	0; 0 N=259	4200; 0 N=260	16800; 0 N=259	28000; 0 N=260	28000; 28000 N=260	Δ_{max}[c]	0; 0	4200; 0	16800; 0	28000; 0	28000; 28000	Δ_{max}[d]
Number of completed visits												
During second trimester pregnancy	-	-	-	-	-	-	1590	1636	1625	1640	1655	-
During third trimester pregnancy	-	-	-	-	-	-	2716	2756	2704	2667	2763	-
During postpartum follow-up	-	-	-	-	-	-	5468	5458	5459	5415	5485	-
Burning sensation or pain in genital tract												
At baseline	24 (9.3)	42 (16.2)	37 (14.3)	18 (6.9)	26 (10.0)	6.9	-	-	-	-	-	-
During second trimester pregnancy	28 (10.9)	41 (15.8)	44 (17.3)	31 (12.2)	42 (16.3)	6.4	46 (2.9)	70 (4.3)	74 (4.6)	52 (3.2)	63 (3.8)	1.7
During third trimester pregnancy	62 (24.5)	75 (29.8)	72 (28.6)	64 (25.1)	77 (30.3)	5.8	106 (3.9)	145 (5.3)	140 (5.2)	111 (4.2)	159 (5.8)	1.9
During postpartum follow-up	36 (15.1)	42 (17.2)	55 (23.1)	35 (14.6)	60 (24.9)	9.8	64 (1.2)	63 (1.2)	99 (1.8)	45 (0.8)	88 (1.6)	0.6
Back pains or cramps												
At baseline	55 (21.2)	75 (28.8)	68 (26.3)	74 (28.5)	69 (26.5)	7.6	-	-	-	-	-	-
During second trimester pregnancy	101 (39.5)	91 (35.1)	112 (44.1)	111 (43.5)	108 (42.0)	4.6	210 (13.2)	221 (13.5)	267 (16.4)	256 (15.6)	269 (16.3)	3.2
During third trimester pregnancy	161 (63.6)	165 (65.5)	166 (65.9)	168 (65.9)	175 (68.9)	5.3	507 (18.7)	529 (19.2)	547 (20.2)	497 (18.6)	550 (19.9)	1.6
During postpartum follow-up	139 (58.2)	145 (59.4)	141 (59.2)	149 (62.1)	142 (58.9)	3.9	459 (8.4)	533 (9.8)	529 (9.7)	533 (9.8)	536 (9.8)	1.4
Clear vaginal fluid												
At baseline	108 (41.7)	111 (42.7)	114 (44.0)	108 (41.5)	114 (43.8)	2.3	-	-	-	-	-	-
During second trimester pregnancy	111 (43.4)	99 (38.2)	113 (44.5)	100 (39.2)	130 (50.6)	7.2	278 (17.5)	297 (18.2)	358 (22.0)	237 (14.5)	331 (20.0)	4.5
During third trimester pregnancy	192 (75.9)	209 (82.9)	209 (82.9)	186 (72.9)	202 (79.5)	7	694 (25.6)	761 (27.6)	778 (28.8)	617 (23.1)	702 (25.4)	3.2
During postpartum follow-up	67 (28.0)	69 (28.3)	64 (26.9)	62 (25.8)	61 (25.3)	2.7	169 (3.1)	149 (2.7)	144 (2.6)	132 (2.4)	147 (2.7)	0.7
Cough												
At baseline	32 (12.4)	36 (13.8)	32 (12.4)	31 (11.9)	37 (14.2)	1.9	-	-	-	-	-	-
During second trimester pregnancy	56 (21.9)	71 (27.4)	62 (24.4)	61 (23.9)	61 (23.7)	5.5	81 (5.1)	124 (7.6)	104 (6.4)	109 (6.6)	90 (5.4)	2.5
During third trimester pregnancy	98 (38.7)	97 (38.5)	78 (31.0)	85 (33.3)	79 (31.1)	7.8	184 (6.8)	173 (6.3)	164 (6.1)	162 (6.1)	141 (5.1)	1.7
During postpartum follow-up	102 (42.7)	107 (43.9)	118 (49.6)	125 (52.1)	107 (44.4)	9.4	244 (4.5)	219 (4.0)	256 (4.7)	243 (4.5)	244 (4.4)	0.4
Abdominal pain												
At baseline	35 (13.5)	47 (18.1)	40 (15.4)	48 (18.5)	47 (18.1)	4.9	-	-	-	-	-	-
During second trimester pregnancy	101 (39.5)	97 (37.5)	89 (35.0)	110 (43.1)	102 (39.7)	4.4	163 (10.3)	185 (11.3)	160 (9.8)	214 (13.0)	192 (11.6)	2.8
During third trimester pregnancy	185 (73.1)	188 (74.6)	184 (73.0)	176 (69.0)	187 (73.6)	4.1	477 (17.6)	524 (19.0)	460 (17.0)	498 (18.7)	521 (18.9)	1.5
During postpartum follow-up	131 (54.8)	146 (59.8)	156 (65.5)	142 (59.2)	145 (60.2)	10.7	279 (5.1)	316 (5.8)	357 (6.5)	308 (5.7)	345 (6.3)	1.4
Constipation												
At baseline	62 (23.9)	81 (31.2)	67 (25.9)	70 (26.9)	66 (25.4)	7.2	-	-	-	-	-	-
During second trimester pregnancy	84 (32.8)	86 (33.2)	76 (29.9)	72 (28.2)	77 (30.0)	4.6	152 (9.6)	171 (10.5)	138 (8.5)	139 (8.5)	152 (9.2)	1.1

Prenatal; Postpartum Vitamin D Dose (IU/Week)	Number of women ever having reported the symptom, n (%)						Number of times the symptom was reported, n (%)[b]					
	0; 0	4200; 0	16800; 0	28000; 0	28000; 28000	Δ_{max}[c]	0; 0	4200; 0	16800; 0	28000; 0	28000; 28000	Δ_{max}[d]
	N=259	N=260	N=259	N=260	N=260							
During third trimester pregnancy	83 (32.8)	84 (33.3)	90 (35.7)	70 (27.5)	89 (35.0)	5.4	180 (6.6)	188 (6.8)	194 (7.2)	163 (6.1)	201 (7.3)	0.6
During postpartum follow-up	117 (49.0)	128 (52.5)	123 (51.7)	134 (55.8)	110 (45.6)	6.9	317 (5.8)	349 (6.4)	312 (5.7)	380 (7.0)	315 (5.7)	1.2
Swelling of hands or feet												
At baseline	17 (6.6)	19 (7.3)	12 (4.6)	14 (5.4)	14 (5.4)	1.9	-	-	-	-	-	-
During second trimester pregnancy	42 (16.4)	29 (11.2)	35 (13.8)	40 (15.7)	36 (14.0)	5.2	76 (4.8)	50 (3.1)	73 (4.5)	85 (5.2)	79 (4.8)	1.7
During third trimester pregnancy	123 (48.6)	119 (47.2)	137 (54.4)	117 (45.9)	120 (47.2)	5.7	478 (17.6)	365 (13.2)	482 (17.8)	472 (17.7)	457 (16.5)	4.4
During postpartum follow-up	35 (14.6)	22 (9.0)	34 (14.3)	26 (10.8)	31 (12.9)	5.6	39 (0.7)	26 (0.5)	39 (0.7)	33 (0.6)	38 (0.7)	0.2
Leg pain or cramps												
At baseline	27 (10.4)	34 (13.1)	32 (12.4)	25 (9.6)	36 (13.8)	3.4	-	-	-	-	-	-
During second trimester pregnancy	70 (27.3)	61 (23.6)	82 (32.3)	79 (31.0)	75 (29.2)	4.9	120 (7.5)	104 (6.4)	144 (8.9)	145 (8.8)	132 (8.0)	1.3
During third trimester pregnancy	115 (45.5)	122 (48.4)	123 (48.8)	120 (47.1)	115 (45.3)	3.4	233 (8.6)	274 (9.9)	318 (11.8)	279 (10.5)	287 (10.4)	3.2
During postpartum follow-up	72 (30.1)	73 (29.9)	71 (29.8)	85 (35.4)	72 (29.9)	5.3	162 (3.0)	174 (3.2)	163 (3.0)	184 (3.4)	167 (3.0)	0.4
Vomiting												
At baseline	67 (25.9)	66 (25.4)	75 (29.0)	58 (22.3)	62 (23.8)	3.6	-	-	-	-	-	-
During second trimester pregnancy	114 (44.5)	116 (44.8)	127 (50.0)	123 (48.2)	113 (44.0)	5.5	211 (13.3)	236 (14.4)	252 (15.5)	243 (14.8)	246 (14.9)	2.2
During third trimester pregnancy	125 (49.4)	131 (52.0)	120 (47.6)	114 (44.7)	126 (49.6)	4.7	295 (10.9)	306 (11.1)	311 (11.5)	283 (10.6)	300 (10.9)	0.6
During postpartum follow-up	52 (21.8)	54 (22.1)	58 (24.4)	42 (17.5)	40 (16.6)	5.2	73 (1.3)	75 (1.4)	71 (1.3)	70 (1.3)	53 (1.0)	0.4
Decreased appetite												
At baseline	77 (29.7)	73 (28.1)	79 (30.5)	82 (31.5)	60 (23.1)	6.7	-	-	-	-	-	-
During second trimester pregnancy	51 (19.9)	49 (18.9)	49 (19.3)	53 (20.8)	55 (21.4)	1.5	83 (5.2)	82 (5.0)	93 (5.7)	84 (5.1)	84 (5.1)	0.5
During third trimester pregnancy	90 (35.6)	94 (37.3)	98 (38.9)	98 (38.4)	79 (31.1)	4.5	158 (5.8)	157 (5.7)	184 (6.8)	200 (7.5)	139 (5.0)	1.7
During postpartum follow-up	58 (24.3)	66 (27.0)	60 (25.2)	49 (20.4)	47 (19.5)	4.8	106 (1.9)	116 (2.1)	111 (2.0)	78 (1.4)	77 (1.4)	0.5
Muscle weakness												
At baseline	32 (12.4)	39 (15.0)	49 (18.9)	50 (19.2)	40 (15.4)	6.9	-	-	-	-	-	-
During second trimester pregnancy	16 (6.3)	21 (8.1)	23 (9.1)	25 (9.8)	30 (11.7)	5.4	18 (1.1)	24 (1.5)	29 (1.8)	34 (2.1)	39 (2.4)	1.2
During third trimester pregnancy	52 (20.6)	43 (17.1)	49 (19.4)	47 (18.4)	40 (15.7)	4.8	70 (2.6)	50 (1.8)	64 (2.4)	55 (2.1)	43 (1.6)	1
During postpartum follow-up	21 (8.8)	28 (11.5)	22 (9.2)	24 (10.0)	25 (10.4)	2.7	40 (0.7)	35 (0.6)	41 (0.8)	41 (0.8)	35 (0.6)	0.1
Severe headache												
At baseline	28 (10.8)	30 (11.5)	27 (10.4)	31 (11.9)	40 (15.4)	4.6	-	-	-	-	-	-
During second trimester pregnancy	35 (13.7)	32 (12.4)	37 (14.6)	43 (16.9)	49 (19.1)	5.4	55 (3.5)	57 (3.5)	60 (3.7)	56 (3.4)	64 (3.9)	0.4
During third trimester pregnancy	39 (15.4)	50 (19.8)	48 (19.0)	50 (19.6)	49 (19.3)	4.4	54 (2.0)	68 (2.5)	83 (3.1)	73 (2.7)	66 (2.4)	1.1
During postpartum follow-up	98 (41.0)	105 (43.0)	108 (45.4)	88 (36.7)	98 (40.7)	4.4	186 (3.4)	207 (3.8)	193 (3.5)	186 (3.4)	196 (3.6)	0.4
Blurry vision during the day												
At baseline	15 (5.8)	15 (5.8)	9 (3.5)	12 (4.6)	13 (5.0)	2.3	-	-	-	-	-	-

Prenatal; Postpartum Vitamin D Dose (IU/Week)	Number of women ever having reported the symptom, n (%)						Number of times the symptom was reported, n (%)[b]					
	0; 0	4200; 0	16800; 0	28000; 0	28000; 28000	Δ_{max}[c]	0; 0	4200; 0	16800; 0	28000; 0	28000; 28000	Δ_{max}[d]
	N=259	N=260	N=259	N=260	N=260							
During second trimester pregnancy	6 (2.3)	11 (4.2)	10 (3.9)	20 (7.8)	10 (3.9)	5.5	8 (0.5)	15 (0.9)	16 (1.0)	29 (1.8)	12 (0.7)	1.3
During third trimester pregnancy	12 (4.7)	16 (6.3)	24 (9.5)	16 (6.3)	16 (6.3)	4.8	15 (0.6)	25 (0.9)	37 (1.4)	31 (1.2)	20 (0.7)	0.8
During postpartum follow-up	16 (6.7)	23 (9.4)	17 (7.1)	21 (8.8)	16 (6.6)	2.7	31 (0.6)	34 (0.6)	25 (0.5)	24 (0.4)	20 (0.4)	0.2
Diarrhea												
At baseline	5 (1.9)	7 (2.7)	5 (1.9)	2 (0.8)	7 (2.7)	1.2	-	-	-	-	-	-
During second trimester pregnancy	27 (10.5)	21 (8.1)	27 (10.6)	27 (10.6)	21 (8.2)	2.4	34 (2.1)	25 (1.5)	28 (1.7)	32 (2.0)	26 (1.6)	0.6
During third trimester pregnancy	48 (19.0)	44 (17.5)	32 (12.7)	38 (14.9)	52 (20.5)	6.3	60 (2.2)	55 (2.0)	41 (1.5)	49 (1.8)	67 (2.4)	0.7
During postpartum follow-up	62 (25.9)	55 (22.5)	59 (24.8)	57 (23.8)	54 (22.4)	3.5	87 (1.6)	74 (1.4)	80 (1.5)	78 (1.4)	64 (1.2)	0.4
Fevers or chills/rigors												
At baseline	6 (2.3)	7 (2.7)	6 (2.3)	10 (3.8)	13 (5.0)	2.7	-	-	-	-	-	-
During second trimester pregnancy	32 (12.5)	31 (12.0)	34 (13.4)	33 (12.9)	34 (13.2)	0.9	36 (2.3)	39 (2.4)	40 (2.5)	41 (2.5)	45 (2.7)	0.5
During third trimester pregnancy	53 (20.9)	39 (15.5)	46 (18.3)	54 (21.2)	49 (19.3)	5.5	68 (2.5)	52 (1.9)	59 (2.2)	69 (2.6)	65 (2.4)	0.6
During postpartum follow-up	106 (44.4)	116 (47.5)	123 (51.7)	102 (42.5)	114 (47.3)	7.3	193 (3.5)	205 (3.8)	220 (4.0)	190 (3.5)	184 (3.4)	0.5
Frequent urination												
At baseline	113 (43.6)	107 (41.2)	112 (43.2)	115 (44.2)	115 (44.2)	2.5	-	-	-	-	-	-
During second trimester pregnancy	54 (21.1)	44 (17.0)	57 (22.4)	57 (22.4)	55 (21.4)	4.1	89 (5.6)	79 (4.8)	117 (7.2)	101 (6.2)	106 (6.4)	1.6
During third trimester pregnancy	143 (56.5)	142 (56.3)	152 (60.3)	149 (58.4)	150 (59.1)	3.8	243 (8.9)	277 (10.1)	293 (10.8)	259 (9.7)	291 (10.5)	1.9
During postpartum follow-up	13 (5.4)	14 (5.7)	14 (5.9)	8 (3.3)	19 (7.9)	2.4	17 (0.3)	19 (0.3)	22 (0.4)	9 (0.2)	27 (0.5)	0.2
Arm pain or cramps												
At baseline	3 (1.2)	5 (1.9)	6 (2.3)	9 (3.5)	4 (1.5)	2.3	-	-	-	-	-	-
During second trimester pregnancy	15 (5.9)	10 (3.9)	12 (4.7)	14 (5.5)	15 (5.8)	2	18 (1.1)	12 (0.7)	14 (0.9)	17 (1.0)	19 (1.1)	0.4
During third trimester pregnancy	39 (15.4)	28 (11.1)	34 (13.5)	27 (10.6)	36 (14.2)	4.8	58 (2.1)	34 (1.2)	51 (1.9)	38 (1.4)	43 (1.6)	0.9
During postpartum follow-up	31 (13.0)	31 (12.7)	37 (15.5)	37 (15.4)	38 (15.8)	2.8	57 (1.0)	47 (0.9)	57 (1.0)	56 (1.0)	74 (1.3)	0.3
Excessive thirst												
At baseline	77 (29.7)	72 (27.7)	81 (31.3)	69 (26.5)	79 (30.4)	3.2	-	-	-	-	-	-
During second trimester pregnancy	37 (14.5)	31 (12.0)	40 (15.7)	45 (17.6)	37 (14.4)	3.2	58 (3.6)	46 (2.8)	69 (4.2)	73 (4.5)	52 (3.1)	0.8
During third trimester pregnancy	75 (29.6)	80 (31.7)	81 (32.1)	77 (30.2)	69 (27.2)	2.5	105 (3.9)	127 (4.6)	132 (4.9)	105 (3.9)	119 (4.3)	1
During postpartum follow-up	23 (9.6)	22 (9.0)	27 (11.3)	22 (9.2)	27 (11.2)	1.7	38 (0.7)	29 (0.5)	40 (0.7)	29 (0.5)	55 (1.0)	0.3
Malodorous or colored vaginal discharge												
At baseline	14 (5.4)	11 (4.2)	16 (6.2)	6 (2.3)	8 (3.1)	3.1	-	-	-	-	-	-
During second trimester pregnancy	7 (2.7)	9 (3.5)	9 (3.5)	6 (2.4)	4 (1.6)	1.2	17 (1.1)	12 (0.7)	12 (0.7)	9 (0.5)	5 (0.3)	0.8
During third trimester pregnancy	17 (6.7)	22 (8.7)	24 (9.5)	15 (5.9)	26 (10.2)	3.5	24 (0.9)	29 (1.1)	31 (1.1)	18 (0.7)	27 (1.0)	0.3
During postpartum follow-up	8 (3.3)	10 (4.1)	15 (6.3)	6 (2.5)	5 (2.1)	3	9 (0.2)	14 (0.3)	41 (0.8)	7 (0.1)	5 (0.1)	0.6
Bleeding from mouth, rectum or in urine												

Prenatal; Postpartum Vitamin D Dose (IU/Week)	Number of women ever having reported the symptom, n (%)						Number of times the symptom was reported, n (%)[b]					
	0; 0	4200; 0	16800; 0	28000; 0	28000; 28000	Δ_{max}[c]	0; 0	4200; 0	16800; 0	28000; 0	28000; 28000	Δ_{max}[d]
	N=259	N=260	N=259	N=260	N=260							
At baseline	3 (1.2)	5 (1.9)	3 (1.2)	4 (1.5)	0 (0.0)	1.2	-	-	-	-	-	-
During second trimester pregnancy	11 (4.3)	4 (1.5)	7 (2.8)	8 (3.1)	3 (1.2)	3.1	16 (1.0)	6 (0.4)	7 (0.4)	13 (0.8)	3 (0.2)	0.8
During third trimester pregnancy	14 (5.5)	11 (4.4)	7 (2.8)	4 (1.6)	10 (3.9)	4	15 (0.6)	12 (0.4)	8 (0.3)	6 (0.2)	13 (0.5)	0.3
During postpartum follow-up	19 (7.9)	18 (7.4)	11 (4.6)	20 (8.3)	10 (4.1)	3.8	25 (0.5)	28 (0.5)	19 (0.3)	40 (0.7)	15 (0.3)	0.3
Vaginal bleeding												
At baseline	0 (0.0)	1 (0.4)	0 (0.0)	3 (1.2)	0 (0.0)	1.2	-	-	-	-	-	-
During second trimester pregnancy	3 (1.2)	0 (0.0)	1 (0.4)	4 (1.6)	1 (0.4)	1.2	4 (0.3)	0 (0.0)	1 (0.1)	9 (0.5)	1 (0.1)	0.3
During third trimester pregnancy	25 (9.9)	26 (10.3)	15 (6.0)	25 (9.8)	30 (11.8)	3.9	25 (0.9)	27 (1.0)	17 (0.6)	31 (1.2)	33 (1.2)	0.3
During postpartum follow-up	66 (27.6)	56 (23.0)	59 (24.8)	72 (30.0)	69 (28.6)	4.7	86 (1.6)	73 (1.3)	90 (1.6)	109 (2.0)	96 (1.8)	0.4
Depressed mood												
At baseline	2 (0.8)	12 (4.6)	10 (3.9)	11 (4.2)	6 (2.3)	3.8	-	-	-	-	-	-
During second trimester pregnancy	12 (4.7)	14 (5.4)	9 (3.5)	7 (2.7)	9 (3.5)	1.9	13 (0.8)	21 (1.3)	13 (0.8)	8 (0.5)	12 (0.7)	0.5
During third trimester pregnancy	16 (6.3)	12 (4.8)	11 (4.4)	9 (3.5)	10 (3.9)	2.8	22 (0.8)	14 (0.5)	11 (0.4)	9 (0.3)	14 (0.5)	0.5
During postpartum follow-up	4 (1.7)	1 (0.4)	6 (2.5)	2 (0.8)	0 (0.0)	1.7	4 (0.1)	1 (0.0)	6 (0.1)	2 (0.0)	0 (0.0)	0.1
Fetal movement (normal)												
At baseline	198 (76.4)	196 (75.4)	188 (72.6)	197 (75.8)	201 (77.3)	3.9	-	-	-	-	-	-
During second trimester pregnancy	255 (99.6)	258 (99.6)	254 (100.0)	255 (100.0)	257 (100.0)	0.4	1560 (98.1)	1599 (97.7)	1579 (97.2)	1597 (97.4)	1619 (97.8)	0.9
During third trimester pregnancy	253 (100.0)	252 (100.0)	252 (100.0)	254 (99.6)	254 (100.0)	0.4	2669 (98.3)	2704 (98.1)	2650 (98.0)	2606 (97.7)	2709 (98.0)	0.6
Labor pains or contractions												
At baseline	0 (0.0)	0 (0.0)	0 (0.0)	0 (0.0)	2 (0.8)	0.8	-	-	-	-	-	-
During second trimester pregnancy	1 (0.4)	1 (0.4)	2 (0.8)	0 (0.0)	2 (0.8)	0.4	1 (0.1)	1 (0.1)	2 (0.1)	0 (0.0)	2 (0.1)	0.1
During third trimester pregnancy	122 (48.2)	123 (48.8)	137 (54.4)	127 (49.8)	133 (52.4)	6.1	127 (4.7)	128 (4.6)	144 (5.3)	137 (5.1)	141 (5.1)	0.6
Difficulty seeing at night												
At baseline	5 (1.9)	1 (0.4)	0 (0.0)	0 (0.0)	3 (1.2)	1.9	-	-	-	-	-	-
During second trimester pregnancy	7 (2.7)	5 (1.9)	2 (0.8)	3 (1.2)	2 (0.8)	2	9 (0.6)	9 (0.6)	2 (0.1)	5 (0.3)	2 (0.1)	0.4
During third trimester pregnancy	5 (2.0)	6 (2.4)	4 (1.6)	4 (1.6)	5 (2.0)	0.4	5 (0.2)	9 (0.3)	4 (0.1)	8 (0.3)	5 (0.2)	0.1
During postpartum follow-up	4 (1.7)	5 (2.0)	3 (1.3)	7 (2.9)	2 (0.8)	1.2	4 (0.1)	8 (0.1)	4 (0.1)	9 (0.2)	2 (0.0)	0.1
Fainting or loss of consciousness												
At baseline	1 (0.4)	1 (0.4)	4 (1.5)	1 (0.4)	0 (0.0)	1.2	-	-	-	-	-	-
During second trimester pregnancy	3 (1.2)	2 (0.8)	1 (0.4)	4 (1.6)	6 (2.3)	1.2	4 (0.3)	2 (0.1)	1 (0.1)	4 (0.2)	7 (0.4)	0.2
During third trimester pregnancy	1 (0.4)	4 (1.6)	4 (1.6)	3 (1.2)	3 (1.2)	1.2	1 (0.0)	5 (0.2)	4 (0.1)	3 (0.1)	3 (0.1)	0.1
During postpartum follow-up	8 (3.3)	4 (1.6)	2 (0.8)	5 (2.1)	1 (0.4)	2.9	9 (0.2)	4 (0.1)	2 (0.0)	5 (0.1)	1 (0.0)	0.1
Bruising												

Prenatal; Postpartum Vitamin D Dose (IU/Week)	Number of women ever having reported the symptom, n (%)						Number of times the symptom was reported, n (%)[b]					
	0; 0	4200; 0	16800; 0	28000; 0	28000; 28000	Δ_{max}[c]	0; 0	4200; 0	16800; 0	28000; 0	28000; 28000	Δ_{max}[d]
	N=259	N=260	N=259	N=260	N=260							
At baseline	1 (0.4)	0 (0.0)	3 (1.2)	2 (0.8)	1 (0.4)	0.8	-	-	-	-	-	-
During second trimester pregnancy	5 (2.0)	6 (2.3)	2 (0.8)	8 (3.1)	7 (2.7)	1.2	7 (0.4)	7 (0.4)	5 (0.3)	10 (0.6)	10 (0.6)	0.2
During third trimester pregnancy	43 (17.0)	52 (20.6)	43 (17.1)	44 (17.3)	47 (18.5)	3.6	49 (1.8)	62 (2.2)	46 (1.7)	53 (2.0)	56 (2.0)	0.4
During postpartum follow-up	2 (0.8)	3 (1.2)	1 (0.4)	5 (2.1)	2 (0.8)	1.2	2 (0.0)	4 (0.1)	1 (0.0)	5 (0.1)	2 (0.0)	0.1
Any fall, injury or trauma												
At baseline	5 (1.9)	3 (1.2)	5 (1.9)	8 (3.1)	3 (1.2)	1.1	-	-	-	-	-	-
During second trimester pregnancy	18 (7.0)	19 (7.3)	19 (7.5)	17 (6.7)	20 (7.8)	0.8	19 (1.2)	20 (1.2)	20 (1.2)	17 (1.0)	21 (1.3)	0.2
During third trimester pregnancy	23 (9.1)	19 (7.5)	14 (5.6)	20 (7.8)	26 (10.2)	3.5	25 (0.9)	20 (0.7)	14 (0.5)	24 (0.9)	27 (1.0)	0.4
During postpartum follow-up	10 (4.2)	12 (4.9)	13 (5.5)	11 (4.6)	9 (3.7)	1.3	10 (0.2)	14 (0.3)	13 (0.2)	11 (0.2)	11 (0.2)	0.1
Mental confusion												
At baseline	1 (0.4)	3 (1.2)	4 (1.5)	0 (0.0)	3 (1.2)	1.2	-	-	-	-	-	-
During second trimester pregnancy	4 (1.6)	0 (0.0)	1 (0.4)	2 (0.8)	2 (0.8)	1.6	5 (0.3)	0 (0.0)	1 (0.1)	2 (0.1)	2 (0.1)	0.3
During third trimester pregnancy	3 (1.2)	4 (1.6)	5 (2.0)	6 (2.4)	8 (3.1)	2	3 (0.1)	4 (0.1)	5 (0.2)	6 (0.2)	8 (0.3)	0.2
During postpartum follow-up	2 (0.8)	0 (0.0)	1 (0.4)	1 (0.4)	0 (0.0)	0.8	2 (0.0)	0 (0.0)	1 (0.0)	3 (0.1)	0 (0.0)	0
Difficult or fast breathing												
At baseline	0 (0.0)	1 (0.4)	0 (0.0)	0 (0.0)	0 (0.0)	0.4	-	-	-	-	-	-
During second trimester pregnancy	7 (2.7)	10 (3.9)	9 (3.5)	9 (3.5)	9 (3.5)	1.1	9 (0.6)	12 (0.7)	12 (0.7)	9 (0.5)	10 (0.6)	0.2
During third trimester pregnancy	18 (7.1)	15 (6.0)	18 (7.1)	17 (6.7)	15 (5.9)	1.2	26 (1.0)	27 (1.0)	24 (0.9)	19 (0.7)	18 (0.7)	0.3
During postpartum follow-up	6 (2.5)	8 (3.3)	9 (3.8)	6 (2.5)	7 (2.9)	1.3	10 (0.2)	10 (0.2)	10 (0.2)	14 (0.3)	8 (0.1)	0.1
Yellow coloration of skin or eyes												
At baseline	0 (0.0)	0 (0.0)	0 (0.0)	0 (0.0)	0 (0.0)	0	-	-	-	-	-	-
During second trimester pregnancy	1 (0.4)	2 (0.8)	1 (0.4)	3 (1.2)	3 (1.2)	0.8	1 (0.1)	2 (0.1)	1 (0.1)	5 (0.3)	4 (0.2)	0.2
During third trimester pregnancy	2 (0.8)	4 (1.6)	9 (3.6)	3 (1.2)	2 (0.8)	2.8	2 (0.1)	4 (0.1)	9 (0.3)	3 (0.1)	2 (0.1)	0.3
During postpartum follow-up	0 (0.0)	0 (0.0)	0 (0.0)	0 (0.0)	0 (0.0)	0	0 (0.0)	0 (0.0)	0 (0.0)	0 (0.0)	0 (0.0)	0
Convulsions												
At baseline	1 (0.4)	0 (0.0)	0 (0.0)	0 (0.0)	0 (0.0)	0.4	-	-	-	-	-	-
During second trimester pregnancy	0 (0.0)	0 (0.0)	1 (0.4)	1 (0.4)	0 (0.0)	0.4	0 (0.0)	0 (0.0)	2 (0.1)	1 (0.1)	0 (0.0)	0.1
During third trimester pregnancy	4 (1.6)	1 (0.4)	1 (0.4)	1 (0.4)	3 (1.2)	1.2	4 (0.1)	1 (0.0)	1 (0.0)	1 (0.0)	3 (0.1)	0.1
During postpartum follow-up	1 (0.4)	2 (0.8)	3 (1.3)	4 (1.7)	4 (1.7)	1.2	1 (0.0)	2 (0.0)	3 (0.1)	5 (0.1)	4 (0.1)	0.1

[a] The second and third trimester of pregnancy refer to >13 to <27 weeks gestation and ≥27 weeks gestation, respectively.

[b] Proportion of number of times the symptom was reported was calculated using the total number of completed weekly visits within each treatment arms in the specified time period as the denominator.

[c] Greatest absolute difference in proportion of women ever having reported the symptom between placebo and any treatment group supplemented with vitamin D.

[d] Greatest absolute difference in proportion of number of times the symptom was reported between placebo and any treatment group supplemented with vitamin D.

Table S33. Frequencies of reported symptoms among infants during the neonatal (≤ 4 weeks of age) and postneonatal (>4 weeks of age) periods based on weekly clinical monitoring during the first 6 months of age

Prenatal; Postpartum Vitamin D Dose (IU/Week)	Number of infants ever with the symptom, n (%)[a]						Number of times the symptom was reported, n (%)[b]					
	0; 0	4200; 0	16800; 0	28000; 0	28000; 28000	Δ_{max}[c]	0; 0	4200; 0	16800; 0	28000; 0	28000; 28000	Δ_{max}[d]
	N = 247	N = 254	N = 252	N = 252	N = 249							
Number of complete visits												
During neonatal period	-	-	-	-	-	-	845	869	851	851	870	-
During postneonatal period	-	-	-	-	-	-	4624	4588	4607	4566	4615	-
Fever or 'too hot' to the touch												
During neonatal period	67 (29.3)	45 (19.1)	47 (20.2)	62 (27.2)	59 (25.0)	10.2	79 (9.3)	55 (6.3)	62 (7.3)	85 (10.0)	83 (9.5)	3
During postneonatal period	198 (83.9)	193 (79.4)	202 (86.0)	217 (91.6)	202 (84.9)	7.7	597 (12.9)	611 (13.3)	621 (13.5)	718 (15.7)	635 (13.8)	2.8
Vomiting												
During neonatal period	75 (32.8)	81 (34.3)	95 (40.8)	90 (39.5)	79 (33.5)	8	130 (15.4)	140 (16.1)	165 (19.4)	141 (16.6)	143 (16.4)	4
During postneonatal period	157 (66.5)	152 (62.6)	165 (70.2)	168 (70.9)	146 (61.3)	5.2	486 (10.5)	542 (11.8)	619 (13.4)	618 (13.5)	477 (10.3)	3
Not gaining enough weight												
During neonatal period	48 (21.0)	53 (22.5)	64 (27.5)	62 (27.2)	57 (24.2)	6.5	73 (8.6)	71 (8.2)	86 (10.1)	89 (10.5)	80 (9.2)	1.8
During postneonatal period	112 (47.5)	96 (39.5)	112 (47.7)	108 (45.6)	103 (43.3)	8	352 (7.6)	302 (6.6)	347 (7.5)	359 (7.9)	272 (5.9)	1.7
Cough												
During neonatal period	51 (22.3)	54 (22.9)	57 (24.5)	64 (28.1)	60 (25.4)	5.8	65 (7.7)	73 (8.4)	74 (8.7)	97 (11.4)	81 (9.3)	3.7
During postneonatal period	209 (88.6)	220 (90.5)	212 (90.2)	219 (92.4)	214 (89.9)	3.8	1004 (21.7)	1036 (22.6)	1108 (24.1)	1151 (25.2)	1035 (22.4)	3.5
Red, oozing and/or swollen eyes												
During neonatal period	50 (21.8)	37 (15.7)	43 (18.5)	35 (15.4)	41 (17.4)	6.5	77 (9.1)	57 (6.6)	64 (7.5)	59 (6.9)	68 (7.8)	2.6
During postneonatal period	62 (26.3)	49 (20.2)	54 (23.0)	52 (21.9)	53 (22.3)	6.1	176 (3.8)	116 (2.5)	116 (2.5)	137 (3.0)	114 (2.5)	1.3
Nasal congestion												
During neonatal period	48 (21.0)	49 (20.8)	42 (18.0)	41 (18.0)	48 (20.3)	3	64 (7.6)	71 (8.2)	63 (7.4)	65 (7.6)	71 (8.2)	0.6
During postneonatal period	201 (85.2)	205 (84.4)	197 (83.8)	201 (84.8)	186 (78.2)	7	1013 (21.9)	916 (20.0)	994 (21.6)	1014 (22.2)	849 (18.4)	3.5
Wheeze or whistling in the chest												
During neonatal period	39 (17.0)	49 (20.8)	40 (17.2)	41 (18.0)	40 (16.9)	3.7	50 (5.9)	71 (8.2)	60 (7.1)	65 (7.6)	63 (7.2)	2.3
During postneonatal period	164 (69.5)	161 (66.3)	170 (72.3)	168 (70.9)	155 (65.1)	4.4	652 (14.1)	612 (13.3)	698 (15.2)	689 (15.1)	641 (13.9)	1.1
Runny nose												
During neonatal period	23 (10.0)	31 (13.1)	20 (8.6)	30 (13.2)	21 (8.9)	3.1	31 (3.7)	41 (4.7)	22 (2.6)	43 (5.1)	27 (3.1)	1.4
During postneonatal period	199 (84.3)	212 (87.2)	209 (88.9)	214 (90.3)	195 (81.9)	6	972 (21.0)	1003 (21.9)	1014 (22.0)	1030 (22.6)	902 (19.5)	1.5
Skin rash												
During neonatal period	54 (23.6)	45 (19.1)	49 (21.0)	62 (27.2)	57 (24.2)	4.5	84 (9.9)	67 (7.7)	73 (8.6)	90 (10.6)	86 (9.9)	2.2
During postneonatal period	32 (13.6)	25 (10.3)	32 (13.6)	27 (11.4)	38 (16.0)	3.3	57 (1.2)	62 (1.4)	47 (1.0)	42 (0.9)	67 (1.5)	0.3
Diarrhea												
During neonatal period	20 (8.7)	20 (8.5)	16 (6.9)	18 (7.9)	12 (5.1)	3.6	27 (3.2)	27 (3.1)	22 (2.6)	21 (2.5)	14 (1.6)	1.6

Prenatal; Postpartum Vitamin D Dose (IU/Week)	Number of infants ever with the symptom, n (%)[a]						Number of times the symptom was reported, n (%)[b]					
	0; 0	4200; 0	16800; 0	28000; 0	28000; 28000	Δ_{max}[c]	0; 0	4200; 0	16800; 0	28000; 0	28000; 28000	Δ_{max}[d]
	N = 247	N = 254	N = 252	N = 252	N = 249							
During postneonatal period	105 (44.5)	112 (46.1)	100 (42.6)	98 (41.4)	100 (42.0)	3.1	181 (3.9)	227 (4.9)	184 (4.0)	192 (4.2)	196 (4.2)	1
Too tired or sleepy												
During neonatal period	10 (4.4)	2 (0.8)	13 (5.6)	13 (5.7)	12 (5.1)	3.5	13 (1.5)	3 (0.3)	13 (1.5)	17 (2.0)	14 (1.6)	1.2
During postneonatal period	8 (3.4)	17 (7.0)	16 (6.8)	19 (8.0)	14 (5.9)	4.6	11 (0.2)	23 (0.5)	19 (0.4)	22 (0.5)	17 (0.4)	0.3
Unusual cry or sounds												
During neonatal period	13 (5.7)	10 (4.2)	14 (6.0)	10 (4.4)	13 (5.5)	1.4	15 (1.8)	11 (1.3)	16 (1.9)	10 (1.2)	14 (1.6)	0.6
During postneonatal period	45 (19.1)	50 (20.6)	61 (26.0)	60 (25.3)	59 (24.8)	6.9	84 (1.8)	91 (2.0)	115 (2.5)	101 (2.2)	106 (2.3)	0.7
Fast breathing												
During neonatal period	2 (0.9)	5 (2.1)	5 (2.1)	5 (2.2)	3 (1.3)	1.3	2 (0.2)	5 (0.6)	6 (0.7)	6 (0.7)	3 (0.3)	0.5
During postneonatal period	23 (9.7)	25 (10.3)	26 (11.1)	16 (6.8)	13 (5.5)	4.3	34 (0.7)	36 (0.8)	38 (0.8)	24 (0.5)	13 (0.3)	0.5
Red or oozing umbilicus												
During neonatal period	19 (8.3)	13 (5.5)	21 (9.0)	13 (5.7)	15 (6.4)	2.8	20 (2.4)	15 (1.7)	26 (3.1)	13 (1.5)	17 (2.0)	0.8
During postneonatal period	6 (2.5)	2 (0.8)	9 (3.8)	3 (1.3)	5 (2.1)	1.7	7 (0.2)	4 (0.1)	16 (0.3)	3 (0.1)	8 (0.2)	0.2
Difficulty breathing												
During neonatal period	5 (2.2)	5 (2.1)	2 (0.9)	8 (3.5)	3 (1.3)	1.3	5 (0.6)	5 (0.6)	2 (0.2)	9 (1.1)	3 (0.3)	0.5
During postneonatal period	13 (5.5)	10 (4.1)	21 (8.9)	12 (5.1)	9 (3.8)	3.4	14 (0.3)	11 (0.2)	28 (0.6)	20 (0.4)	10 (0.2)	0.3
Poor feeding												
During neonatal period	7 (3.1)	4 (1.7)	7 (3.0)	6 (2.6)	6 (2.5)	1.4	8 (0.9)	4 (0.5)	7 (0.8)	7 (0.8)	6 (0.7)	0.5
During postneonatal period	17 (7.2)	23 (9.5)	24 (10.2)	21 (8.9)	11 (4.6)	3	22 (0.5)	27 (0.6)	33 (0.7)	30 (0.7)	17 (0.4)	0.2
'Too cold' to the touch												
During neonatal period	7 (3.1)	3 (1.3)	2 (0.9)	2 (0.9)	2 (0.8)	2.2	7 (0.8)	3 (0.3)	2 (0.2)	2 (0.2)	2 (0.2)	0.6
During postneonatal period	4 (1.7)	2 (0.8)	5 (2.1)	5 (2.1)	4 (1.7)	0.9	4 (0.1)	2 (0.0)	5 (0.1)	5 (0.1)	4 (0.1)	0
Not passed any urine in the past 6 hours												
During neonatal period	1 (0.4)	2 (0.8)	5 (2.1)	2 (0.9)	4 (1.7)	1.7	1 (0.1)	2 (0.2)	5 (0.6)	2 (0.2)	4 (0.5)	0.5
During postneonatal period	5 (2.1)	2 (0.8)	1 (0.4)	7 (3.0)	1 (0.4)	1.7	6 (0.1)	2 (0.0)	1 (0.0)	8 (0.2)	1 (0.0)	0.1
Injury or trauma												
During neonatal period	2 (0.9)	3 (1.3)	3 (1.3)	1 (0.4)	1 (0.4)	0.4	3 (0.4)	3 (0.3)	3 (0.4)	1 (0.1)	1 (0.1)	0.2
During postneonatal period	7 (3.0)	11 (4.5)	12 (5.1)	7 (3.0)	12 (5.0)	2.1	7 (0.2)	11 (0.2)	14 (0.3)	7 (0.2)	13 (0.3)	0.2
Blood in the stool												
During neonatal period	0 (0.0)	0 (0.0)	1 (0.4)	2 (0.9)	0 (0.0)	0.9	0 (0.0)	0 (0.0)	1 (0.1)	2 (0.2)	0 (0.0)	0.2
During postneonatal period	7 (3.0)	6 (2.5)	10 (4.3)	10 (4.2)	9 (3.8)	1.3	9 (0.2)	6 (0.1)	14 (0.3)	15 (0.3)	10 (0.2)	0.1
Convulsion or unresponsive												
During neonatal period	1 (0.4)	1 (0.4)	4 (1.7)	2 (0.9)	1 (0.4)	1.3	1 (0.1)	1 (0.1)	4 (0.5)	2 (0.2)	1 (0.1)	0.4
During postneonatal period	1 (0.4)	0 (0.0)	2 (0.9)	0 (0.0)	1 (0.4)	0.4	1 (0.0)	0 (0.0)	2 (0.0)	0 (0.0)	1 (0.0)	0

Prenatal; Postpartum Vitamin D Dose (IU/Week)	Number of infants ever with the symptom, n (%)[a]						Number of times the symptom was reported, n (%)[b]					
	0; 0	4200; 0	16800; 0	28000; 0	28000; 28000	Δ_{max}[c]	0; 0	4200; 0	16800; 0	28000; 0	28000; 28000	Δ_{max}[d]
	N = 247	N = 254	N = 252	N = 252	N = 249							
Not feeding at all in past 6 hours												
During neonatal period	2 (0.9)	1 (0.4)	2 (0.9)	1 (0.4)	3 (1.3)	0.4	2 (0.2)	1 (0.1)	2 (0.2)	1 (0.1)	3 (0.3)	0.1
During postneonatal period	2 (0.8)	3 (1.2)	0 (0.0)	1 (0.4)	0 (0.0)	0.8	3 (0.1)	3 (0.1)	0 (0.0)	1 (0.0)	0 (0.0)	0.1

[a] Proportion of infants ever having been reported as having the symptom by the caregiver was calculated using the number of live births as the denominator.

[b] Proportion of number of times the symptom was reported was calculated using the total number of times the symptom was reported across all treatment arms as the denominator.

[c] Greatest absolute difference in proportion of women ever having reported the symptom between placebo and any treatment group supplemented with vitamin D.

[d] Greatest absolute difference in proportion of number of times the symptom was reported between placebo and any treatment group supplemented with vitamin D.

References

1. Shi J, Korsiak J, Roth DE. New approach for the identification of implausible values and outliers in longitudinal childhood anthropometric data. *Annals of Epidemiology* 2018: 28(3):204-211.e3.
2. Schleicher RK, Encisco SE, Chaudhary-Webb M, Paliakov E, McCoy LF, Pfeiffer CM. Isotope dilution ultra performance liquid chromatography-tandem mass spectrometry method for simultaneous measurement of 25-hydroxyvitamin D2, 25-hydroxyvitamin D3 and 3-epi-25-hydroxyvitamin D3 in human serum. *Clinica Chimica Acta; International Journal of Clinical Chemistry* 2011; **412**: 1594-1599.
3. Yazdanpanah M, Bailey D, Walsh W, Wan B, Adeli K. Analytical measurement of serum 25-OH-vitamin D-3, 25-OH-vitamin D-2 and their C3-epimers by LC-MS/MS in infant and pediatric specimens. *Clinical Biochemistry* 2013; **46**: 1264-1271.
4. Dunteman GH. Principle Component Analysis. Newbury Park, California: Sage Publications; 1989.
5. Filmer D, Pritchett L. Estimating Wealth Effects Without Expenditure Data-Or Tears: An Application to Educational Enrollments in States of India. *Demography* 2001; **38**(1): 115–32.
6. Roth DE, Gernand AD, Morris SK, Pezzack B, Islam MM, Dimitris MC, et al. Maternal vitamin D supplementation during pregnancy and lactation to promote infant growth in Dhaka, Bangladesh (MDIG trial): study protocol for a randomized controlled trial. *Trials* 2015; **16**: 300.
